# Endothelial cannabinoid CB1 receptor deficiency reduces shear stress-induced arterial inflammation and lipid uptake

**DOI:** 10.1038/s41467-026-75214-2

**Published:** 2026-07-07

**Authors:** Bingni Chen, Aishvaryaa Prabhu, Guo Li, Anna Kaltenbach, Yong Wang, George Shakir, Lucia Natarelli, Remco Megens, Yvonne Jansen, Srishti Ramanathan, Martina Geiger, Alexander Faussner, Michael Hristov, Daniel Richter, Xinyu Di, Mario van der Stelt, Vasiliki Triantafyllidou, Zhaolong Li, Nadja Sachs, Valentina Paloschi, Lars Maegdefessel, Susanna M. Hofmann, Martina Schifferer, Mikael Simons, Christian Weber, Donato Santovito, Stephan Herzig, Raquel Guillamat Prats, Sabine Steffens

**Affiliations:** 1 Institute for Cardiovascular Prevention, LMU University Hospital, LMU Medizin, Ludwig-Maximilians-Universität Munich, Munich, Germany; 2 DZHK (German Center for Cardiovascular Research), partner site Munich Heart Alliance, Munich, Germany; 3 Department of Biomedical Engineering, Cardiovascular Research Institute Maastricht (CARIM), Maastricht University Medical Centre, Maastricht, Netherlands; 4 Anthropology and Human Genomics, Faculty of Biology, Ludwig-Maximilians-Universität Munich, Planegg-Martinsried, Germany; 5 Department of Molecular Physiology, Leiden University and Oncode Institute, Leiden, Netherlands; 6 Department of Vascular and Endovascular Surgery, Klinikum rechts der Isar, Technical University Munich (TUM), Munich, Germany; 7 Institute for Diabetes and Regeneration, Helmholtz Diabetes Center, Helmholtz Zentrum Munich, Neuherberg, Germany; 8 German Center for Diabetes Research (DZD), Neuherberg, Germany; 9 Department of Medicine IV, LMU University Hospital, LMU Medizin, Ludwig-Maximilians-Universität Munich, Munich, Germany; 10 German Center for Neurodegenerative Diseases, Munich, Germany; 11 Munich Cluster of Systems Neurology (SyNergy), Munich, Germany; 12 Institute of Neuronal Cell Biology, Technical University Munich, Munich, Germany; 13 Institute for Stroke and Dementia Research, LMU University Hospital, LMU Medizin, Ludwig-Maximilians-Universität Munich, Munich, Germany; 14 Department of Biochemistry, Cardiovascular Research Institute Maastricht (CARIM), Maastricht University Medical Centre, Maastricht, The Netherlands; 15 Institute for Diabetes and Cancer, Helmholtz Zentrum Munich, Neuherberg, Germany; 16 Chair Molecular Metabolic Control, Technical University Munich (TUM), Munich, Germany; 17 Joint Heidelberg-IDC Translational Diabetes Program, Heidelberg University Hospital, Heidelberg, Germany

**Keywords:** Atherosclerosis, Experimental models of disease, Molecular medicine

## Abstract

Peripheral cannabinoid CB1 receptor antagonists that lack central nervous system effects are emerging as promising therapies for metabolic disease, yet the role of endothelial CB1 signaling in atherosclerosis remains unclear. Here, we show that endothelial CB1 is expressed in human atherosclerotic plaques, is induced by oscillatory shear stress in atheroprone flow regions, and promotes vascular inflammation, permeability and lipid uptake. Endothelial-specific *Cnr1* deletion or peripheral CB1 antagonism in mice attenuates atherosclerosis, reduces endothelial caveolae–dependent low-density lipoprotein uptake by downregulating caveolin-1 and ALK1 expression, and improves metabolic parameters in brown and white adipose tissue and the liver. The anti-atherogenic and metabolic effects are more pronounced in females, which is possibly linked to estrogen signaling. These findings identify endothelial CB1 as a proatherogenic, sex-biased regulator of vascular lipid transport and plaque development and associated metabolic dysfunction.

## Abstract (web summary)

Previous studies identified CB1 as a target for metabolic disease. We identified endothelial CB1 as a regulator of vascular lipid transport, atherosclerosis, and metabolic dysfunction, with effects more pronounced in females.

## Introduction

Atherosclerosis is a lipid-driven, pro-inflammatory autoimmune disease characterized by the progressive accumulation of lipids, inflammatory cells, and fibrous material in the subendothelial space of large arteries, leading to life-threatening complications, including myocardial infarction, stroke, and peripheral artery disease^1^. Accumulation of low-density lipoprotein (LDL) in the arterial wall and endothelial dysfunction are thought to be key mechanisms that initiate the disease^2^. Atherosclerotic lesions develop preferentially at arterial branches or curvatures of blood vessels, where blood flow is disturbed, further highlighting the importance of biomechanical factors in the initiation of the disease^3^. Shear stress, the frictional force generated by the blood flow on the endothelial surface, is sensed by mechanoreceptors and induces an intracellular signaling response. In atheroprone regions with disturbed flow, the endothelium is exposed to low, oscillatory shear stress, which induces inflammatory signaling through activation of the nuclear factor-kappa-B (NF-κB) pathway. In contrast, atheroprotective shear stress induces several negative regulators of inflammatory pathways, including the transcription factor Kruppel-like family 2 (KLF2)^4^. As a result, in regions of atheroprotective shear stress, the endothelium has a quiescent phenotype with a preserved barrier function, whereas low, oscillatory shear stress leads to an activated phenotype with a relocalization of intercellular junctional complexes and increased permeability^2,3^.

A key regulator linking biomechanical and inflammatory pathways to LDL transport into the arterial wall is endothelial caveolin 1 (CAV1), the major component of caveolae^5^. Caveolae are bulb-shaped, shear stress-sensitive signaling domains in the plasma membrane of endothelial cells (ECs)^5^. Genetic loss of *Cav-1* reduced LDL infiltration into the arterial wall, promoted nitric oxide production, and reduced the expression of adhesion molecules, while all these effects were reversed by endothelial-specific *Cav1* transgene expression^6^. CAV1 is required for caveolae formation and caveolae-mediated LDL uptake and transcytosis across the endothelium^7^. LDL transcytosis pathways involve scavenger receptor B1 (SR-B1) and activin-like kinase 1 (ALK1), which are localized within caveolae and directly bind LDL to facilitate the transport of intact LDL across ECs^8,9^. This is independent of the canonical LDLR-dependent LDL endocytosis pathway with lysosomal degradation and instead mediates LDL translocation across the endothelial layer to become trapped in the vessel wall^5^.

Endocannabinoids, a group of arachidonic acid-derived lipid mediators that bind to the G protein-coupled receptor CB1, play an important role in energy homeostasis and metabolic health^10^. In individuals with obesity, elevated endocannabinoid levels have been positively correlated with coronary endothelial dysfunction^11^. CB1 expression is increased in the hearts of individuals with obesity and mice across all key cell types, including the endothelium^12^. Pharmacologic blockade of CB1 signaling with the antagonist rimonabant inhibited plaque formation in a mouse model of atherosclerosis^13^. Other investigators did not find an effect on plaque size, but observed an improved aortic endothelium-dependent vasodilation and decreased aortic ROS production and NADPH oxidase activity in mice treated with a CB1 antagonist^14^. However, the beneficial use of global CB1 antagonists has been hampered by serious side effects, particularly depression and anxiety, due to the inhibition of CB1 signaling in the brain.

Peripherally restricted CB1 antagonists have been developed and shown to ameliorate metabolic dysfunction in mouse models^15,16^. Furthermore, adipocyte-specific deletion of the CB1-encoding gene *Cnr1* was sufficient to protect mice from diet-induced obesity and associated metabolic alterations^17^. We have recently shown that myeloid-specific *Cnr1* deficiency limits atherosclerosis by inhibiting the recruitment and proliferation of arterial macrophages^18^. Sugamura and colleagues previously reported that the systemically acting CB1 antagonist rimonabant exerted anti-inflammatory effects in macrophages^19^, which was corroborated by another study^13^. However, the specific role of endothelial CB1 in atherogenesis and related metabolic alterations remains poorly defined. Here, we show that endothelial-specific *Cnr1* knockout mitigates the endothelial shear stress response, plaque development, and caveolae-dependent LDL uptake, which can be recapitulated using a peripheral CB1 antagonist.

## Results

### Endothelial CB1 expression is induced by atheroprone shear stress

**Fig. 1:**
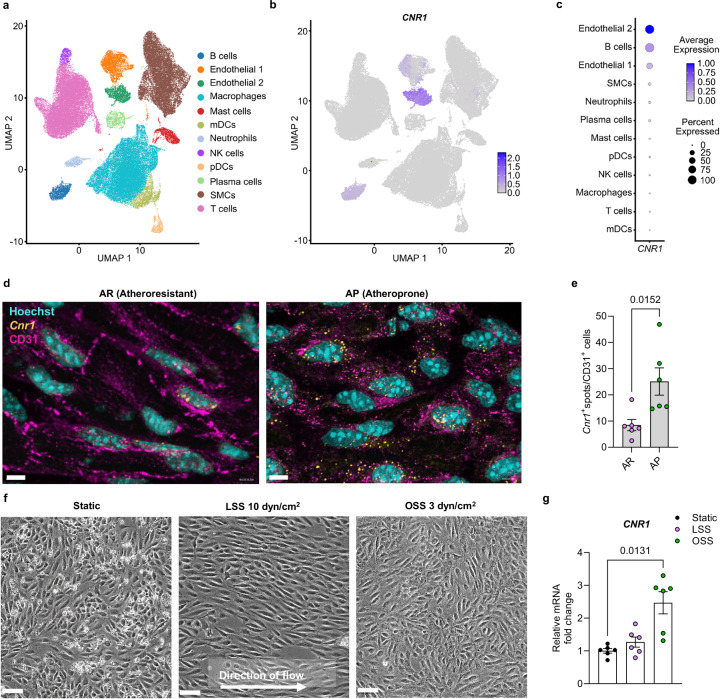
*CNR1* expression in human atherosclerotic carotid plaques and impact of shear stress on endothelial *CNR1* expression. **a–c** Single-cell gene expression data were retrieved from a published data set of carotid atherosclerotic plaques from 18 patients who underwent endarterectomy (GSE253904). **a** Clusters of major immune cell types (including myeloid dendritic cells [mDCs] and plasmacytoid dendritic cells [pDCs]), endothelial cells, and smooth muscle cells (SMCs) visualized using the Uniform Manifold Approximation and Projection (UMAP). **b** Expression pattern of *CNR1* in individual clusters. **c** Dot plot depicting the relative expression of *CNR1* in each cluster. **d** En face in situ hybridization for *Cnr1* within the descending thoracic aorta (atheroresistant, AR) and aortic arch (atheroprone, AP) of the same female *Apoe*^*-/-*^ mouse. Scale bar, 5 μm. **e** Quantification of *Cnr1*+ spot per CD31+ cell from panel d. 10-15 images per mouse were acquired for quantification (*n* = 6). **f** Representative images of HAoECs (*n* = 6) from female donors after 24 hours exposure to LSS (10 dyn/cm^2^) or OSS (3 dyn/cm^2^) compared to the static condition. Scale bar, 50 μm. **g** *CNR1* gene expression levels in HAoECs from female donors after exposure to different shear stresses (*n* = 6). Data are shown as mean ± s.e.m.; two- tailed Mann-Whitney *U* (**e**) or one-way ANOVA with Tukey *post hoc* tests (**g**) were applied. Each data point represents one individual mouse or human sample (biological replicate), collected in at least 2 independent experiments.

While CB1 expression has been reported in human plaques^19^, the specific cell types contributing to CB1 signaling within the plaque have not been well characterized. Therefore, we analyzed publicly available single-cell gene expression data (GSE253904)^20^ of carotid atherosclerosis lesions from 18 patients who underwent endarterectomy (total 73,833 cells) and found that the CB1-encoding gene *CNR1* was detectable in an endothelial cell cluster (endothelial 2; Fig. 1a–c) co-expressing gene markers typical of arterial endothelium, such as *BMX*, *MECOM*, and *GJA5* (Supplementary Fig. 1a), whereas *CNR1* expression was very low in the pro-angiogenic endothelial cluster (endothelial 1) expressing venular markers (*ACKR1*, *RGCC*, *NR2F2*)^21^. Lower levels were detected in B cells, while other immune cell types exhibited a significantly reduced *CNR1* expression, although the expression has previously been demonstrated in a variety of immune cells^22^ and more recently in murine plaque macrophages by in situ hybridization^18^. The prominent expression of *CNR1* in ECs compared with other cell types was confirmed in an independent single-cell RNA sequencing data set of human atherosclerotic plaques (GSE247238; Supplementary Fig. 1b, c)^23^.

ECs exhibit distinct phenotypes instructed by their specific location within the artery. In the descending thoracic aorta, the endothelium is exposed to a uniform and laminar blood flow, whereas within the inner curvature of the aortic arch or at branch points, the blood flow is disturbed^24^. We investigated whether the expression of endothelial CB1 is affected by shear stress. To this end, we first performed an *en face* in situ hybridization to detect the mRNA expression of endothelial *Cnr1* in different shear stress regions within the aorta of 8-week-old *Apoe*^*-/-*^ mice. A more intense endothelial *Cnr1* expression signal was detected in the atheroprone region of the aortic arch, compared to the atheroresistant region of the descending thoracic aorta (Fig. 1d, e, Supplementary Fig. 2a, b). This observation suggests that the expression of endothelial *Cnr1* is regulated by shear stress.

To further demonstrate this regulation, we cultured HAoECs in different shear stress conditions. After 24 h of exposure to laminar shear stress (LSS), HAoECs had a more elongated shape and were aligned in flow direction as opposed to cells cultured in static conditions, whereas OSS resulted in a cobblestone-like morphology (Fig. 1f). In line with the in vivo findings in *Apoe*^*-/-*^ aortas, 2-fold higher *CNR1* mRNA levels were observed in HAoECs exposed to OSS compared to static culture or LSS, while LSS did not affect *CNR1* expression (Fig. 1g). In agreement with increased CB1 activation upon OSS, a significant increase in the mRNA levels of the endocannabinoid 2-AG synthesis enzyme *DAGL* was also observed in this condition, while no changes in the degradation enzyme *MGLL* were detectable in response to different shear stress conditions (Supplementary Fig. 2c). Collectively, these data indicate that endothelial 2-AG/CB1 signaling is induced by OSS in atheroprone regions of the aorta.

### Endothelial *Cnr1* deficiency affects vascular and cardiac function

Next, we generated endothelial cell-specific *Cnr1*-deficient mice by crossing *Cnr1*^*flox/flox*^ mice^25^ with *Bmx*^*CreERT*^ mice^26^ on an *Apoe*^*-/-*^ background (*Apoe*^*-/-*^*Bmx*^*Cre(+/-)*^*Cnr1*^*flox/flox*^), hereafter referred to as *Cnr1*^*EC-KO*^ mice. Tamoxifen-induced recombination at 8 weeks of age resulted in arterial endothelial cell-specific deletion of *Cnr1* (Supplementary Fig. 2d)^26^. Tamoxifen was also administered to the control group (*Apoe*^*-/-*^*Bmx*^*Cre(+/-)*^ mice, designated *Cnr1*^*EC-WT*^). *Cnr1* deletion was confirmed by in situ hybridization in *Cnr1*^*EC-KO*^ aortic roots, and the endothelial CB1 expression in *Cnr1*^*EC-WT*^ mice was confirmed by colocalization of the *Cnr1* probe with the endothelial marker CD31 (Supplementary Fig. 2e).

To perform a basic cardiovascular phenotyping of the new mouse line, we measured cardiac function and blood flow dynamics in *Cnr1*^*EC-KO*^ and *Cnr1*^*EC-WT*^ mice by echocardiography. While *Cnr1*^*EC-WT*^ mice showed impaired cardiac function after 4 weeks of Western diet (WD), characterized by significantly reduced fractional shortening and a tendency for reduced ejection fraction (*p* = 0.06). These parameters were preserved in *Cnr1*^*EC-KO*^ mice, which did not show an impairment of cardiac function parameters after WD (Supplementary Fig. 3a-d). In baseline conditions, we observed a higher peak flow velocity in atheroprone aortic regions of *Cnr1*^*EC-KO*^ mice compared to *Cnr1*^*EC-WT*^ mice (Supplementary Fig. 3e–g). After 4 weeks of WD feeding, the peak flow velocity dropped in atheroprone regions of the aortic arch and aortic roots of *Cnr1*^*EC-KO*^ mice under WD, whereas short-term WD feeding did not affect peak flow velocity in *Cnr1*^*EC-WT*^. No diet- or genotype-dependent effects were observed in atheroresistant regions of the aortic arch. Together, these findings suggest that endothelial *Cnr1* deficiency affects the regulation of blood flow dynamics.

### Endothelial *Cnr1* deficiency affects morphology and permeability

**Fig. 2:**
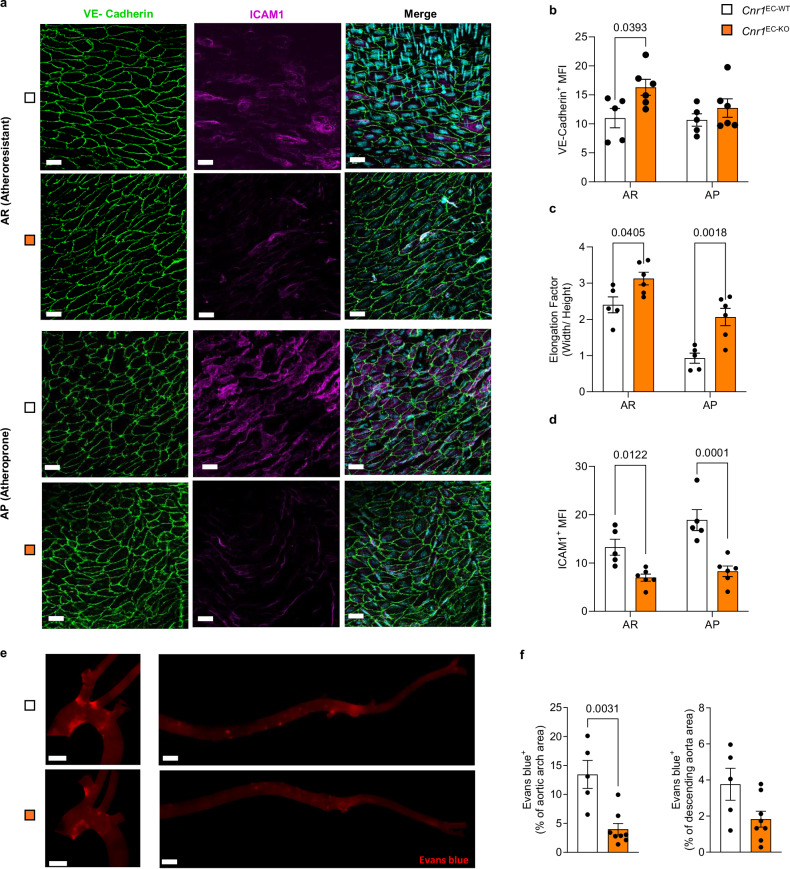
Impact of endothelial *Cnr1* deficiency on endothelial morphology and integrity. **a** The aortic arch (atheroprone, AP) and descending thoracic aorta (atheroresistant, AR) were isolated from female *Cnr1*^*EC-WT*^ mice (*n* = 5) and *Cnr1*^*EC-KO*^ mice (*n* = 6) and prepared for *en face* staining. Staining for VE-cadherin (green), ICAM1 (magenta), and DAPI (cyan) to visualize the intercellular junctions, adhesion molecules, and nuclei. The images were captured by confocal microscopy. Scale bar, 20 μm. **b**–**d** The mean fluorescence intensity (MFI) of VE-Cadherin and ICAM1 signals was quantified from maximum projections of images generated from Z-stack (10-15 images per region per mouse were captured) with Leica Application Suite X (LAS X) software. EC elongation factor (of 20 randomly selected cells per image) was quantified from female *Cnr1*^*EC-WT*^ mice (*n* = 5) and *Cnr1*^*EC-KO*^ mice (*n* = 6). **e** Representative images of the endothelial permeability assay, based on Evan´s blue extravasation into aortas 30 min after i.v. injection. Scale bar, 1 mm. **f** Quantification of Evan´s blue-positive area normalized to aortic arch or descending aorta area of female *Cnr1*^*EC-WT*^ (*n* = 5) and *Cnr1*^*EC-KO*^ mice (*n* = 8). Images were taken with a Leica DM6000B microscope and quantified by LAS V4.3 software. All data are shown as mean ± s.e.m; Two-way ANOVA with Sidak *post hoc* (**b**–**d**) or two- tailed Mann-Whitney *U* (**f**) tests were applied. Each data point represents one individual mouse sample (biological replicate), collected in at least 2 independent experiments.

Given that OSS induces endothelial CB1 expression, we asked whether the absence of endothelial CB1 would in turn affect the endothelial phenotype in atheroprone aortic regions. Therefore, we performed *en face* staining for the EC junctional protein vascular endothelial (VE)-cadherin and the adhesion molecule ICAM1 using aortic arches and thoracic aortas isolated from *Cnr1*^*EC-KO*^ and *Cnr1*^*EC-WT*^ mice. Increased VE-cadherin expression was observed in the atheroresistant descending thoracic aorta of *Cnr1*^*EC-KO*^ and *Cnr1*^*EC-WT*^ mice (Fig. 2a, b). ECs in *Cnr1*^*EC-KO*^ aortas were more elongated than those in *Cnr1*^*EC-WT*^ aortas (Fig. 2c). Furthermore, less endothelial ICAM1 expression was found in aortas of *Cnr1*^*EC-KO*^ mice, suggesting an anti-inflammatory phenotype when endothelial *Cnr1* is depleted (Fig. 2d). Because vascular inflammation impairs endothelial barrier function, we subsequently monitored vascular leakage by injecting Evan´s Blue into *Cnr1*^*EC-KO*^ and *Cnr1*^*EC-WT*^ mice after 4 weeks of WD. Significantly less vascular leakage was observed in the aortic arch of *Cnr1*^*EC-KO*^ compared to *Cnr1*^*EC-WT*^ mice, indicating preserved endothelial integrity in *Cnr1*^*EC-KO*^ mice (Fig. 2e, f).

### *Cnr1*^*EC-KO*^ endothelial cells show profound transcriptomic changes

**Fig. 3:**
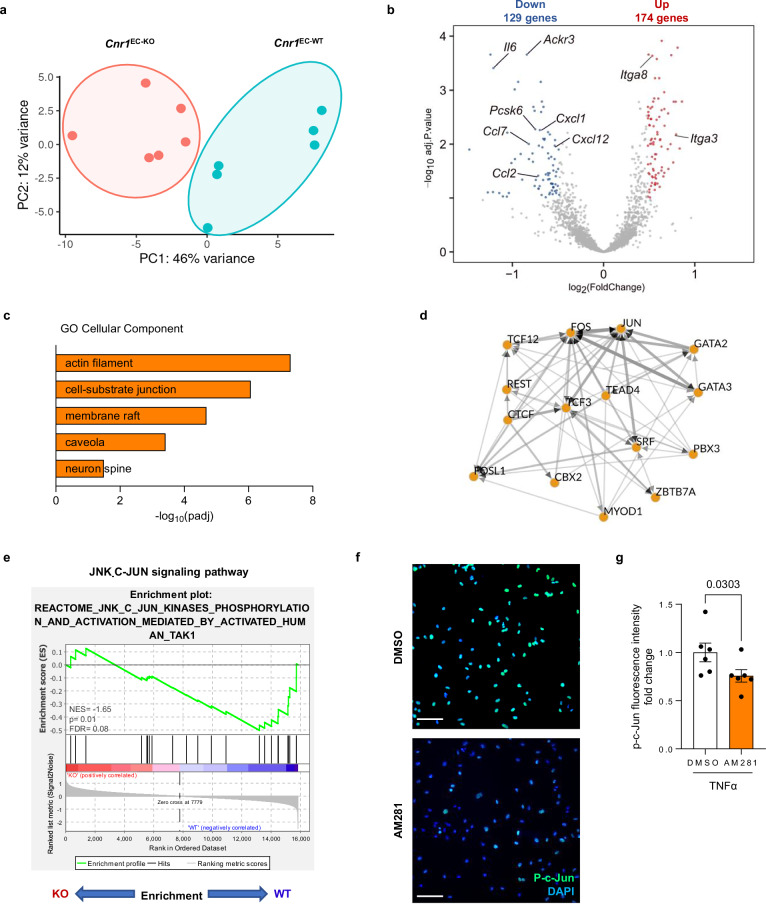
Transcriptomic profile of aortic endothelial *Cnr1* signaling. **a**–**e** Aortic ECs (sorted as CD45^-^CD107a^low^CD31^high^) were isolated from female *Cnr1*^*EC-WT*^ and *Cnr1*^*EC-KO*^ mouse aortas (*n* = 6) after 4 weeks of WD for RNA sequencing. **a** Principal component analysis (PCA) of aortic ECs from female *Cnr1*^*EC-WT*^ and *Cnr1*^*EC-KO*^ mice. **b** Volcano plot showing differentially expressed genes (DEGs) with 174 significantly up- and 129 down-regulated genes (FDR 1%) in female *Cnr1*^*EC-WT*^ and *Cnr1*^*EC-KO*^ mice. Statistical significance was assessed using a two-sided Wald test, and *P* values were adjusted for multiple testing using the Benjamini–Hochberg FDR method. Genes with FDR < 0.10 were considered significant. **c** GO enrichment analysis was performed using clusterProfiler based on DEGs identified by DESeq2. Enrichment was assessed using a one-sided hypergeometric test, and *P* values were adjusted for multiple comparisons using the Benjamini–Hochberg FDR method. **d** Prediction of the top 15 transcription factor (TF) -co-regulatory networks modulated by endothelial *Cnr1* deficiency using CHEA3. **e** Pathway **e**nrichment (GSEA) with normalized enrichment score (NES) and FDR q-value. *P* values were adjusted for multiple testing using the Benjamini–Hochberg FDR method, and pathways with NES <−1 or > 1 and FDR *q*-value < 0.15 were considered significant. **f** Phospho-c-Jun immunostaining (green) of HAoECs from female donors pretreated with vehicle or 1 μM AM281 for 5 min prior to 30 min TNFα stimulation. Nuclei were stained with DAPI (blue); scale bar, 20 μm. **g** Quantification of **f** (*n* = 6). Data are shown as mean ± s.e.m. and a two-tailed Mann-Whitney *U* test was applied. Each data point represents one individual mouse or human sample (biological replicate), collected in at least 2 independent experiments.

To globally assess the transcriptomic profile regulated by endothelial CB1 signaling, we sorted aortic ECs from *Cnr1*^*EC-KO*^ and *Cnr1*^*EC-WT*^ mice after 4 weeks of WD and performed RNA sequencing. Principal component analysis showed distinct clustering of *Cnr1*^*EC-KO*^ and *Cnr1*^*EC-WT*^ samples (Fig. 3a). We found 303 differentially expressed genes (DEGs) between the two groups, of which 129 were downregulated and 174 were upregulated in *Cnr1*^*EC-KO*^ compared to *Cnr1*^*EC-WT*^ ECs. Notably, the expression of several pro-inflammatory cytokines and chemokines, such as *Il6*, *Ackr3*, *Cxcl12*, and *Ccl2*, was significantly lower in *Cnr1*^*EC-KO*^ ECs, consistent with a less inflammatory phenotype (Fig. 3b). Conversely, genes associated with the cellular matrix, including *Itga8* and *Itga3*, and the transcription factor *Prdm16*, which plays a critical role in maintaining endothelial function and supporting arterial flow recovery^27^, were upregulated in *Cnr1*^*EC-KO*^ ECs. Taken together, these findings suggest a complex network of transcriptomic changes that may underlie the CB1-dependent regulation of EC homeostatic function.

Further gene ontology (GO) analysis revealed that the transcriptomic signature regulated by endothelial *Cnr1* deficiency affects key cellular components, including membrane raft (GO:0045121; padj = 2.05e^-05^) and caveola (GO:0005901; padj = 0.0003), suggesting that CB1 may affect endothelial lipid raft and caveolae-dependent signaling (Fig. 3c). To identify putative upstream transcription factors (TFs) of the transcriptomic signature in *Cnr1*-deficient ECs, ChIP-X Enrichment Analysis 3 (CHEA3) was performed (Fig. 3d). *Fos* and *Jun* emerged as the primary TFs regulating the DEGs network. These TFs are known as activator protein 1 (AP-1) regulators and mediate various cellular processes such as cytokine and chemokine expression, as well as cell migration and differentiation. In addition, GSEA revealed enrichment of DEGs in the JNK_c-JUN, Il6_JAK_Stat3, inflammatory, and NFκB signaling pathways (Fig. 3e, Supplementary Fig. 4a). The regulation of c-JUN activation by CB1 was further investigated in HAoECs treated with the CB1 antagonist AM281, which significantly inhibited TNFα-induced nuclear translocation of phosphorylated c-JUN (Fig. 3f, g and Supplementary Fig. 4b).

To validate our findings, we investigated the regulation of key pro-inflammatory markers among the identified DEGs in HAoECs after transfection with *CNR1* or scrambled siRNA. A knockdown efficiency of approximately 80 % was achieved with *CNR1* siRNA (Supplementary Fig. 4c). In *CNR1*-silenced HAoECs, the expression of the pro-inflammatory genes *CXCL8*, *CCL2* and *ICAM1* was significantly attenuated. (Supplementary Fig. 4d), confirming a reduced pro-inflammatory phenotype in the absence of endothelial CB1 signaling. Because increased vascular inflammation disrupts vascular homeostasis and induces endothelial oxidative stress^28^, we tested whether CB1 signaling affects ROS production. Flow cytometry analysis revealed a reduction in TNF-α-stimulated ROS production in HAoECs after *CNR1* knockdown (Supplementary Fig. 4e). To further confirm that CB1 signaling promotes endothelial inflammation, we treated HAoECs with the synthetic CB1 agonist ACEA in LSS culture conditions and found that CB1 stimulation prevented the atheroprotective effects of LSS exposure. While LSS downregulated the expression of *ICAM1*, vascular cell adhesion molecule-1 (*VCAM1*), and glycolytic enzyme *PFKFB3* compared to the static condition, this effect was attenuated by ACEA (Supplementary Fig. 4f). In addition, ACEA prevented LSS-induced upregulation of *KLF2* and *NOS3* (Supplementary Fig. 4f). Comparable expression levels were detected in vehicle- and ACEA-treated ECs under static conditions, suggesting that CB1 activation under shear stress inhibited the LSS-mediated anti-inflammatory phenotype. To support these findings, an adhesion assay was performed with HAoECs perfused with labeled THP-1 monocytes in the presence or absence of the CB1 agonist ACEA under LSS. A significantly increased number of adherent monocytes was observed when stimulating HAoECs with ACEA compared to the vehicle control (Supplementary Fig. 4g, h). Overall, our results demonstrated that impaired CB1 signaling exhibits an anti-inflammatory phenotype in both human and murine ECs.

### Endothelial *Cnr1* deficiency reduces atherosclerotic plaque formation

**Fig. 4:**
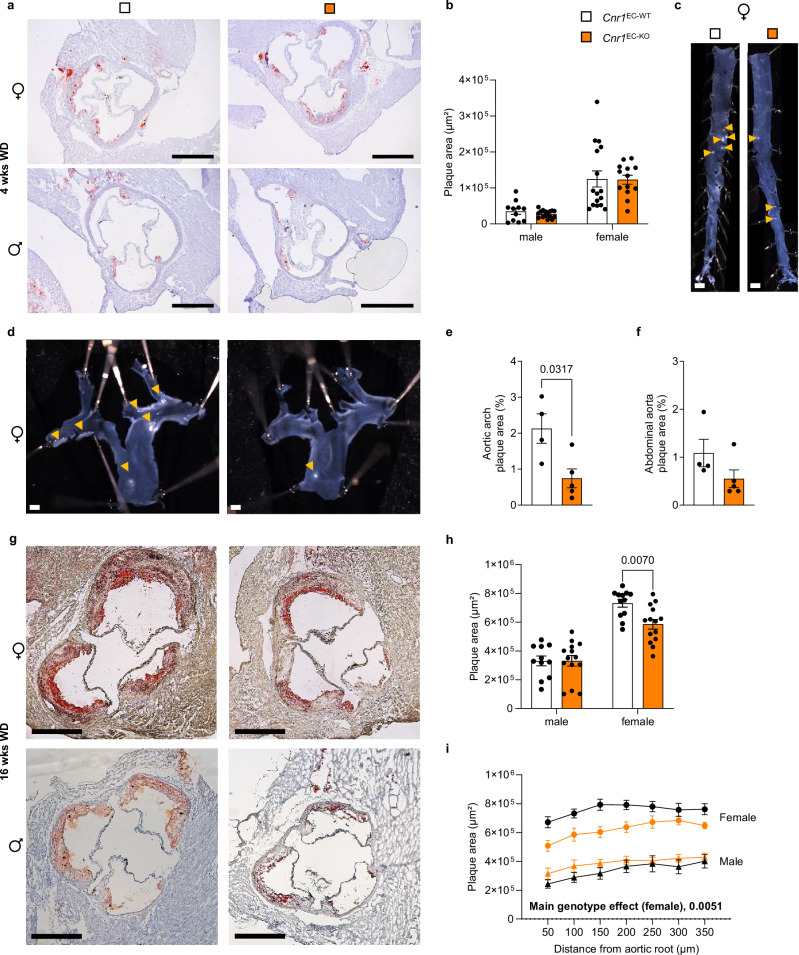
Effect of endothelial *Cnr1* deficiency on atherosclerotic plaque development. **a** Representative Oil-Red-O (ORO) stainings (scale bar, 500 μm) and **b** quantification of absolute lesion area in aortic roots from *Cnr1*^*EC-WT*^ (male: *n* = 11, female: *n* = 16) and *Cnr1*^*EC-KO*^ (male: *n* = 16, female: *n* = 13) mice after 4 weeks of WD. **c**–**f** Representative images and analysis of arch and descending aorta lesion area normalized to vessel area from female *Cnr1*^*EC-WT*^ (*n* = 4) and *Cnr1*^*EC-KO*^ (*n* = 5) after 4 weeks of WD. Scale bar, 1 mm. Mann-Whitney U was performed. **g** Representative ORO staining and analysis of aortic roots of male and female *Cnr1*^*EC-WT*^ and *Cnr1*^*EC-KO*^ after 16 weeks of WD; Scale bar, 500 μm. **h** Quantification of absolute lesion area from *Cnr1*^*EC-WT*^ (male: *n* = 11, female: *n* = 12) and *Cnr1*^*EC-KO*^ (both sexes *n* = 14) mice shown in (**g**). **i** Plaque area per aortic root section from and *Cnr1*^*EC-KO*^ (male: *n* = 10, female: *n* = 8) shown in (**g**) (7 sections per root at 50 μm distance from each other were analyzed). All data are shown as mean ± s.e.m.; Two-way ANOVA with Sidak post hoc tests (**b**, **h**), two-tailed Mann-Whitney *U (***d**–**f**) or mixed-effect model with a separate analysis of the main fixed effect (genotype) for males and females (**i**) were applied. Each data point represents one individual mouse sample (biological replicate), collected in at least 2 independent experiments.

To address whether the reduced inflammatory phenotype in the absence of endothelial CB1 translates into reduced atherosclerotic lesion development, we subjected *Cnr1*^*EC-KO*^ and *Cnr1*^*EC-WT*^ mice to a WD for 4 weeks or 16 weeks to induce early and advanced stages of atherosclerosis, respectively. At the 4-week time point, male and female *Cnr1*^*EC-KO*^ mice exhibited plaque sizes comparable to those of *Cnr1*^*EC-WT*^ mice in the aortic roots, whereas significantly smaller plaques were found in the aortic arch of female *Cnr1*^*EC-KO*^ mice and descending aortas of male *Cnr1*^*EC-KO*^ mice compared to corresponding sex-matched *Cnr1*^*EC-WT*^ mice (Fig. 4a–f and Supplementary Fig. 5). After 16 weeks of atherogenic diet, female *Cnr1*^*EC-KO*^ mice exhibited smaller atherosclerotic plaques in the aortic roots compared to corresponding *Cnr1*^*EC-WT*^ controls (Fig. 4g–i), whereas no difference was observed in male mice. This suggests that loss of endothelial *Cnr1* ameliorates atherosclerosis in a stage- and site-specific manner, and that the phenotype is more evident in female mice. To exclude any effects of the *loxP* insertion in *Cnr1*^*flox/flox*^ mice on the atherosclerotic phenotype*, Apoe*^*-/-*^*Cnr1*^*flox/flox*^ mice were used as an additional control group (Supplementary Fig. 6a). Notably, measurement of plasma endocannabinoid levels in male and female *Apoe*^*-/-*^ mice after 4 weeks of WD revealed comparable levels of 2-arachidonoylglycerol, anandamide, oleoylethanolamide, and palmitoylethanolamide (Supplementary Fig. 6b). Thus, the more pronounced anti-atherosclerotic effect of endothelial *Cnr1* deficiency in females cannot be explained by sex-specific differences in systemic endocannabinoid levels. However, endocannabinoids are locally produced on demand and act in an autocrine and paracrine manner^29^, which means that systemic plasma levels do not necessarily reflect the local production and CB1 activation within the vessel wall.

Surprisingly, male and female *Cnr1*^*EC-KO*^ mice had significantly higher circulating plasma cholesterol levels compared to the *Cnr1*^*EC-WT*^ control group (Supplementary Fig. 6c), especially in the LDL fraction (Supplementary Fig. 6d). Because hypercholesterolemia promotes arterial inflammation and leukocytosis through enhanced hematopoietic progenitor proliferation in a mechanism that involves cholesterol sensing pathways^1,30^, this finding was unexpected in light of the reduced plaque size observed in female mice. However, no significant differences in circulating leukocyte counts were observed between the *Cnr1*^*EC-KO*^ and *Cnr1*^*EC-WT*^ groups (Supplementary Fig. 6e), ruling out that endothelial *Cnr1* deficiency promotes leukocytosis. We hypothesized that the increase in circulating plasma cholesterol may be a consequence of reduced tissue lipid uptake. Reduced aortic lipid uptake may explain reduced plaque development despite elevated plasma cholesterol levels. To characterize advanced aortic root lesions after 16 weeks of WD, we assessed intracellular lipid droplets, necrotic core size, collagen and macrophage content. Significant differences were found in the plaque composition of female *Cnr1*^*EC-KO*^ mice, in particular a significant reduction in relative lipid plaque content and an increase in collagen content (Supplementary Fig. 7a–d). These findings suggest a shift towards a more stable plaque phenotype, possibly due to a reduced aortic lipid accumulation.

### Endothelial *Cnr1* deficiency improves metabolic parameters

**Fig. 5:**
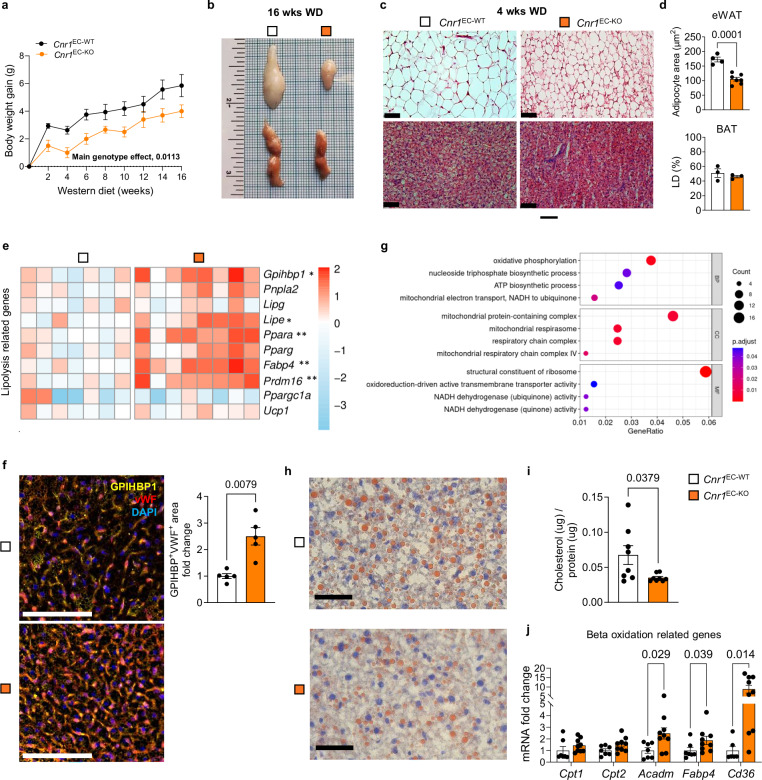
Impact of endothelial *Cnr1* deficiency on lipid metabolism. **a** Body weight gain over 16 weeks WD in female *Cnr1*^*EC-WT*^ (*n* = 8) and *Cnr1*^*EC-KO*^ (*n* = 7) mice. **b** Representative images of epididymal white (eWAT, upper) and brown adipose tissue (BAT, lower) from female mice after 16 weeks WD. **c** H&E staining of eWAT and BAT from female *Cnr1*^*EC-WT*^ and *Cnr1*^*EC-KO*^ after 4 weeks of WD. Scale bar, 500 μm. **d** Quantification of adipocyte size in eWAT: *Cnr1*^*EC-WT*^ (*n* = 4) and *Cnr1*^*EC-KO*^ (*n* = 7) and relative lipid droplet (LD) content in BAT: *Cnr1*^*EC-WT*^ and *Cnr1*^*EC-KO*^ (*n* = 3 for both) from **c**. **e** Gene expression analysis (RT-qPCR) in BAT of female *Cnr1*^*EC-WT*^ (*n* = 7) and *Cnr1*^*EC-KO*^ (*n* = 8) mice after 4 weeks WD. Log_2_-transformed values visualized in a heatmap plotted by using pheatmap in R. **f** Co-staining of GPIHBP1 (yellow) and von Willebrand factor (vWF, red for capillary vessels in BAT of female *Cnr1*^*EC-WT*^ and *Cnr1*^*EC-KO*^ (*n* = 5) mice after 4 weeks of WD. Scale bar, 100 μm. Quantification of GPIHBP1^+^VWF^+^ area and calculated as fold change to WT. **g** RNA sequencing and GO analysis of regulated pathways in sorted BAT ECs from female *Cnr1*^*EC-KO*^ (*n* = 7) mice after 4 weeks of WD. DEGs were identified using DESeq2 with a two-sided Wald test followed by Benjamini–Hochberg FDR correction. GO enrichment analysis was performed using clusterProfiler with a one-sided hypergeometric test and Benjamini–Hochberg FDR adjustment. **h** Representative hepatic ORO staining and **i** liver cholesterol levels in female *Cnr1*^*EC-WT*^ and *Cnr1*^*EC-KO*^ (*n* = 8 for both sexes) mice after 4 weeks of WD Scale bar, 50 μm. **j** Gene expression analysis (RT-qPCR) in livers of female *Cnr1*^*EC-KO*^ (*n* = 7) and *Cnr1*^*EC-WT*^ (*n* = 9) mice after 4 weeks WD. All data are shown as mean ± s.e.m.; mixed-effect model with analysis of the main fixed effect-genotype (**a**), two-tailed Mann-Whitney *U* (**d**, **f**, **i**) and multiple Mann-Whitney U with FDR-Hochberg, Krieger, Yekutielli (**j**) or T test with FDR-Benjamini Hochberg (**e**) were applied. Each data point represents one individual mouse sample (biological replicate), collected in at least 2 independent experiments.

We found that the depletion of *Cnr1* in ECs was sufficient to attenuate the body weight gain over 16 weeks WD in female and male mice (Fig. 5a and Supplementary Fig. 8a), as previously reported in global *Cnr1-*deficient mice subjected to a high fat diet. *Cnr1*^*EC-KO*^ mice had less white and brown fat mass (Fig. 5b and Supplementary Fig. 8b). Histological analysis of female epididymal white adipose tissue at 4 weeks WD revealed a significant reduction in adipocyte size of *Cnr1*^*EC-KO*^ mice, whereas the relative lipid droplet content in brown adipose tissue (BAT) was not different (Fig. 5c, d). At the same time point, we detected higher mRNA expression of *Gpihbp1*, a capillary endothelial cell protein that facilitates lipolysis of triglyceride-rich lipoproteins, as well as other lipolysis-related genes in the BAT of *Cnr1*^*EC-KO*^ female mice, which did not reach significance in male mice (Fig. 5e and Supplementary Fig. 8c). There was no significant difference in the ATGL encoding gene (*Pnpla2*) and several other markers of lipogenesis or lipolysis (*Lpl*, *Angptl4*, *Angptl8*, *Fas*, *Fat1*; data not shown).

Increased capillary endothelial GPIHBP1 expression in the BAT of female *Cnr1*^*EC-KO*^ mice was confirmed by immunostaining (Fig. 5f). The transcription factor *Prdm16*, which is involved in adipose tissue browning and thermogenesis^31,32^, as well as *Fabp4*, which promotes thermogenesis^33^, were also significantly upregulated in the BAT of *Cnr1*^*EC-KO*^ female mice (Fig. 5e and Supplementary Fig. 8c). This prompted us to further investigate the underlying EC-specific transcriptional changes. To this end, we sorted ECs from the BAT of female C*nr1*^*EC-KO*^ and C*nr1*^*EC-WT*^ mice at 4 weeks WD for RNA sequencing. The GO analysis revealed that the pathways regulated by endothelial CB1 in the BAT were related to mitochondrial respiration and ATP biosynthesis, suggesting increased mitochondrial activity in the BAT of C*nr1*^*EC-KO*^ mice (Fig. 5g). Examination of the liver phenotype in female C*nr1*^*EC-KO*^ mice revealed reduced hepatic accumulation of lipid droplets (Fig. 5h) and decreased hepatic cholesterol levels at 4 weeks WD (Fig. 5i), while plasma bile acid levels were not affected by endothelial *Cnr1* deficiency when analyzed in both males and females (Supplementary Fig. 8d). Liver qPCR analysis at the same time point showed a significant increase in the expression of the fatty acid transporter *Cd36* and the marker *Acadm* (acyl-CoA dehydrogenease medium chain) linked to hepatic β-oxidation in female C*nr1*^*EC-KO*^ mice (Fig. 5j). In addition, an intraperitoneal glucose tolerance test (ipGTT) revealed improved glucose disposal rates in C*nr1*^*EC-KO*^ male and female mice compared to the age-matched C*nr1*^*EC-WT*^ mice (Supplementary Fig. 8e, f).

### Endothelial *Cnr1* deficiency reduces caveolae-mediated LDL uptake

**Fig. 6:**
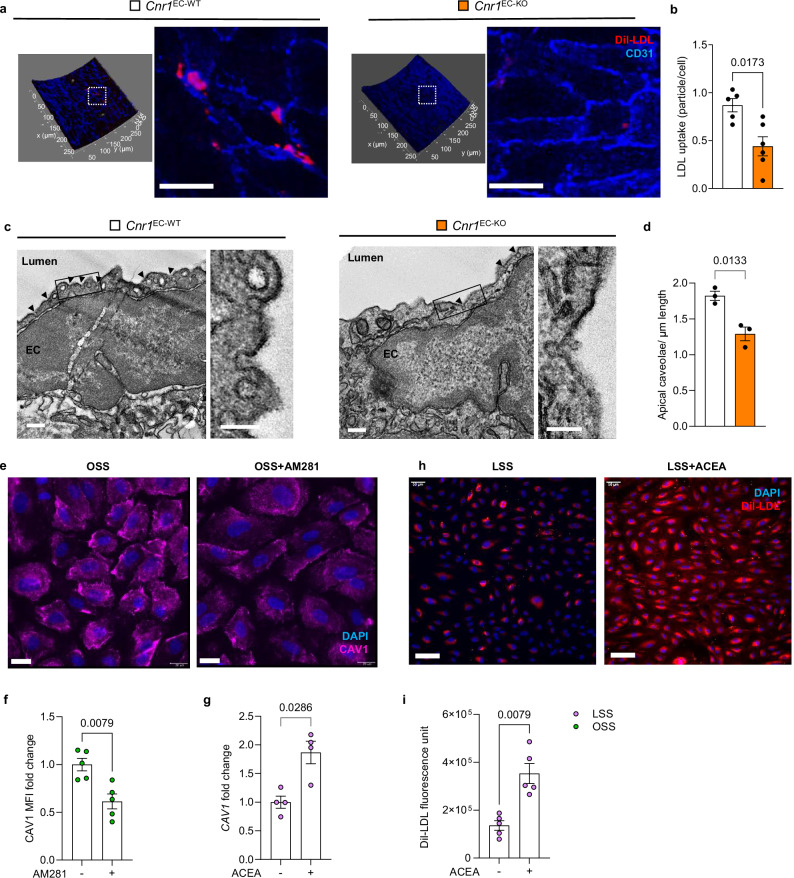
Role of CB1 in endothelial LDL transport. **a** Representative TPLSM 3D images of Dil-LDL particles in ex vivo perfused carotid arteries from female *Cnr1*^*EC-WT*^ and *Cnr1*^*EC-KO*^ mice after 4 weeks WD; CD31 was used to mark ECs. Scale bar, 20 μm. **b** Quantification of Dil-LDL normalized to EC number (*Cnr1*^*EC-WT*^ *n* = 5 and *Cnr1*^*EC-KO*^ mice *n* = 6). **c** TEM images visualizing endothelial caveolae from aortic arches of female *Cnr1*^*EC-WT*^ and *Cnr1*^*EC-KO*^ mice after 4 weeks WD (scale bar: overview- 200 nm, zoom in- 100 nm) EC- endothelial cell. **d** Quantification of apical caveolae from **c** (*n* = 3 mice per group collected in a single experiment; each dot represents an average of 20 analyzed images) **e**, **f** Representative images of HAoECs from female donors and quantification of CAV1 mean fluorescence intensity (MFI) (*n* = 5) treated with 1 μM AM281 or vehicle under OSS for 24 h followed by 1ug/ml Dil-LDL treatment for 30 min. Scale bar, 20 μm. **g** *CAV1* mRNA expression (RT-qPCR) in HAoECs from female donors (*n* = 4) treated with 1 μM ACEA or vehicle under LSS for 24 h. **h**, **i** Representative images of HAoECs from female donors and quantification of Dil-LDL uptake (by absolute fluorescence unit) (*n* = 5) preincubated with 1 µM ACEA or vehicle under LSS for 24 hours, followed by 1 µg/mL Dil-LDL treatment for 90 min. Scale bar, 50 μm. Data are shown as mean ± s.e.m; two-tailed Mann-Whitney *U* test (**b**, **f**, **g**, **i**) or Welch T- test (**d**) was applied. Each data point represents one individual mouse or human sample (biological replicate), collected in at least 1 (c) or 2 (**a**, **e**, **h**) independent experiments.

To confirm our hypothesis that the increase in circulating plasma cholesterol and reduced plaque lipid content in *Cnr1*^EC-KO^ mice may be a consequence of reduced tissue lipid uptake, we perfused carotid arteries from female C*nr1*^*EC-KO*^ and C*nr1*^*EC-WT*^ mice isolated at 4 weeks WD with fluorescently labeled native LDL (Dil-LDL)^34^. Two-photon laser scanning microscopy (TPLSM) revealed that lack of *Cnr1* in ECs resulted in significantly reduced retention of LDL particles across the endothelial layer (Fig. 6a, b). To further investigate the underlying CB1-dependent regulation of endothelial LDL uptake, we screened for candidate receptor expression levels in our RNA sequencing data of sorted C*nr1*^*EC-KO*^ and C*nr1*^*EC-WT*^ aortic ECs and found a significantly lower expression level of *Acvrl1*, which encodes the protein activing A receptor like type 1, also termed activing-like kinase receptor (ALK1, Supplementary Fig. 9a). We did not find any transcriptional regulation of other endothelial LDL receptors including the canonical LDLR (Supplementary Fig. 9a). In agreement with the transcriptomic data, no differences in the surface expression levels of LOX-1, SRB1, SRA1, and CD36 were found between C*nr1*^*EC-KO*^ and C*nr1*^*EC-WT*^ aortic eECs (Supplementary Fig. 9b, c).

In support of CB1-dependent regulation of LDL uptake, the GO analysis of the aortic EC transcriptomic data indicated a regulation of membrane rafts and caveola in *Cnr1*-deficient ECs (Fig. 3c). CAV1, a major component of caveolae, co-localizes with ALK1 within caveolae-enriched domains of ECs^5^. Genetic deficiency of *Cav1* has been shown to inhibit aortic DiI-LDL uptake in isolated aortas and atherosclerosis despite elevated total cholesterol levels^35,36^. To address a possible regulation of cavelolae, we conducted transmission electron microscopy of C*nr1*^*EC-KO*^ and C*nr1*^*EC-WT*^ aortic arches collected after 4 weeks WD. The quantification of apical caveolae in atheroprone aortic arch regions confirmed that deficiency of *Cnr1* reduces their number on the endothelial luminal site (Fig. 6c, d). We additionally performed co-immunostaining of ECs and CAV1 in aortic root sections from C*nr1*^*EC-KO*^ and C*nr1*^*EC-WT*^ mice at the 4 week WD time point. CAV1 expression in C*nr1*^*EC-KO*^ ECs was significantly reduced in female mice (Supplementary Fig. 9d, e), providing a possible mechanism for reduced endothelial LDL uptake in C*nr1*^*EC-KO*^ mice. Notably, no significant difference was observed in male mice (Supplementary Fig. 9d, e).

To further investigate the regulation of CAV1 and LDL uptake by CB1, HAoECs were treated with the CB1 antagonist AM281 under OSS, which resulted in significantly reduced CAV1 expression (Fig. 6e, f). Conversely, when HAoECs were subjected to LSS, activation of CB1 with the agonist ACEA resulted in increased mRNA expression of *CAV1* (Fig. 6g) and increased DiI-LDL uptake (Fig. 6h, i). Furthermore, we found that siRNA-mediated silencing of the ALK1-encoding gene *ACVRL1* in HAoECs blunted the effect of the CB1 agonist ACEA in inducing DiI-LDL uptake (Supplementary Fig. 9f, g). Taken together, these results support a direct regulation of endothelial caveolae-mediated LDL uptake by CB1 through modulation of *CAV1* gene expression.

### Endothelial CB1 induces caveolae-mediated LDL uptake via cAMP-PKA signaling

CB1 has been described in various cell lines as a G_i_ protein-coupled receptor that decreases intracellular cyclic adenosine monophosphate (cAMP) upon agonist binding^37–39^. We first validated the CB1-dependent regulation of intracellular cAMP levels in response to CB1 activation or antagonism, respectively, in a HEK293 reporter cell line stably transfected with a cAMP-luciferase reporter plasmid and the human *CNR1* cDNA. The CB1 antagonist AM281 induced a significant increase in intracellular cAMP levels, whereas the CB1 agonist ACEA decreased intracellular cAMP levels, which was most evident when the adenylate cyclase activator forskolin was added shortly after agonist or antagonist administration (Supplementary Fig. 10a). In HAoECs, endogenous cAMP levels were measured by ELISA, which showed that AM281 dose-dependently increased intracellular cAMP levels under static conditions, while ACEA showed only modestly decreased endogenous cAMP levels, possibly due to intrinsic activation of CB1 by endocannabinoids under basal conditions (Supplementary Fig. 10b). These data confirm that pharmacological inhibition of endothelial CB1 signaling leads to an increase in intracellular levels of cAMP.

**Fig. 7:**
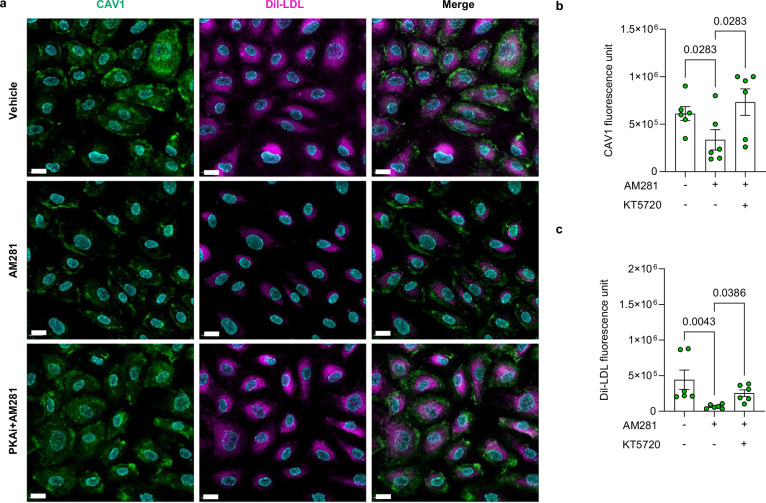
Role of PKA in CB1-dependent regulation of endothelial CAV1 expression and LDL transport. **a** Representative immunofluorescence analysis of CAV1 expression and Dil-LDL uptake in HAoECs of female donors treated with 1 μM AM281 alone or in the presence of 1 μM PKA inhibitor (KT5720) or vehicle (DMSO) under OSS for 24 hours. Scale bar, 20 μm. Quantification of **b** CAV1 fluorescence intensity and **c** LDL uptake fold change (*n* = 6). Data are shown as mean ± s.e.m.; Kruskal-Wallis *H* test with Dunn’s *post hoc* (**b**, **c**) test was applied. Each data point represents one individual human sample (biological replicate), collected in at least 2 independent experiments.

It has been previously reported that the activation of protein kinase A (PKA) leads to a reduction in CAV1 expression in Chinese hamster ovary cells^40^. PKA is activated upon binding of cAMP to its regulatory subunits^41^. To clarify whether endothelial CAV1 expression is regulated by CB1 via cAMP-PKA signaling, HAoECs cells were treated with KT5720, a PKA inhibitor, before the addition of AM281 under OSS conditions. The reduction in endothelial CAV1 expression by AM281 treatment was prevented by the addition of KT5720 (Fig. 7a, b). Similarly, the reduction in LDL uptake by AM281 treatment was prevented by pretreatment with KT5720 (Fig. 7c), suggesting that the reduction in CAV1 requires PKA activation in ECs. Notably, all the above-described in vitro experiments were performed with primary human cells from female donors. When the same experiments were repeated with HAoECs from an age-matched male donor, no significant effects on CAV1 expression were observed, only reduced LDL uptake (Supplementary Fig. S11a, b).

### Chronic peripheral CB1 antagonism reduces atheroprogression, endothelial CAV1 and ICAM1 expression in female *Ldlr*^*-/-*^ mice

**Fig. 8:**
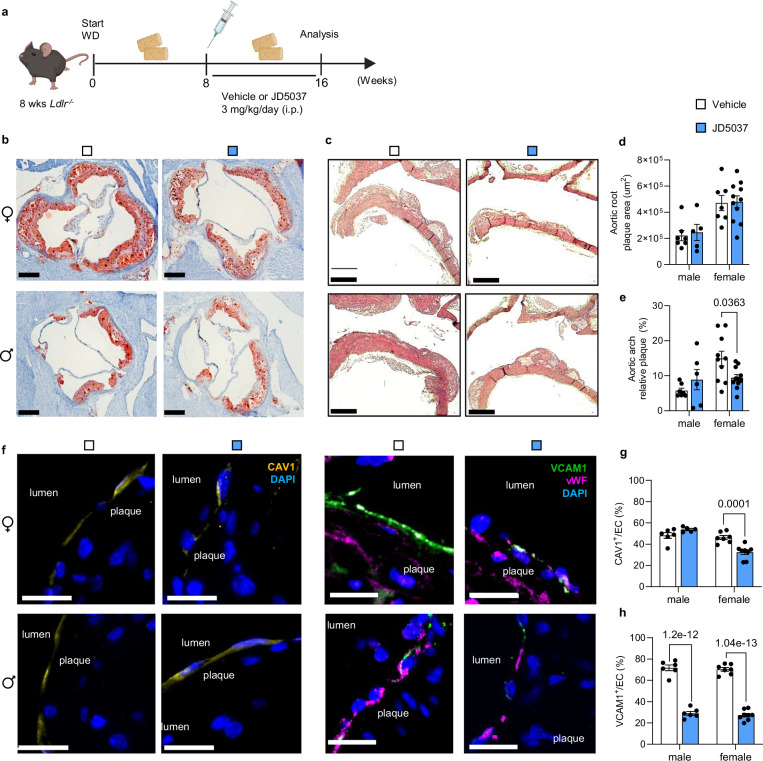
Effect of chronic peripheral CB1 antagonist administration on atherosclerosis and CAV1 expression. **a** Schematic experimental design scheme (Created in BioRender. Prabhu, A. (2026) https://BioRender.com/esv7wyx). Male and female *Ldlr*^*−/−*^ mice were fed with WD for 16 weeks and received daily intraperitoneal injected JD5037 (3 mg/kg) or vehicle for the last 8 weeks. **b** Representative ORO-stained images of aortic root cross-sections (scale bar, 500 μm) and **c** H&E-stained aortic arch longitudinal sections (scale bar, 500 μm). **d** Quantification of absolute lesion areas in aortic roots (vehicle: *n* = 7 both sexes; JD5037: male *n* = 5, female *n* = 11) and **e** aortic arches (vehicle: male *n* = 7, female *n* = 10; JD5037: male *n* = 6, female *n* = 11) **f** Representative images of CAV1 (yellow), VCAM (green) and vWF (magenta) in aortic arch plaques; nuclei were stained with DAPI (scale bar, 20 μm). **g**, **h** Quantification of CAV1 (vehicle: male *n* = 6, female *n* = 7; JD5037: male *n* = 5, female *n* = 8) and VCAM1 (vehicle: male *n* = 6, female *n* = 7; JD5037: male *n* = 6, female *n* = 8) positive ECs. All data are shown as mean ± s.e.m.; Two-way ANOVA with Sidak *post hoc* test (**d**, **e**, **g**, **h**) was applied. Each data point represents one individual mouse sample (biological replicate), collected in at least 2 independent experiments.

To clarify whether the peripheral CB1 antagonist JD5037 would reproduce an atheroprotective phenotype as observed with endothelial *Cnr1* deficiency, we first subjected *Ldlr*^*-/-*^ mice to a WD for 8 weeks to induce atherosclerotic lesions, followed by an additional 8-weeks of JD5037 treatment in parallel with continuous WD (Fig. 8a). We used the *Ldlr*^*-/*-^ atherosclerotic mouse model for this therapeutic approach because it more closely mirrors the lipid profile in humans compared to the *Apoe*^*-/-*^ model^42^. Consistent with our previous study focusing on atheroprotective effects of peripheral CB1 antagonism on myeloid cells at an earlier time point, chronic administration of the peripheral CB1 antagonist JD5037 resulted in significantly less body weight gain^18^, but plasma total cholesterol and triglyceride levels were not different after 16 weeks of WD (Supplementary Fig. 12a, d).

No significant effects of JD5037 treatment on plaque size progression were observed in the aortic root compared to the corresponding sex-matched vehicle group. However, a reduction in plaque progression in the aortic arch was observed in female *Ldlr*^*-/*-^ mice treated with JD5037; this effect was not seen in male mice (Fig. 8b–e). Consistent with the observations in C*nr1*^*EC-KO*^ mice, reduced plaque size in female JD5037-treated mice was accompanied by decreased levels of CAV1, ICAM1, and VCAM1 in aortic ECs (Fig. 8f–h and Supplementary Fig. 12e). A significant reduction in endothelial VCAM1 expression was also observed in male JD5037-treated mice compared to the vehicle group (Fig. 8h). These results suggest that peripheral CB1 antagonism blocks the proatherogenic effects of CB1 in arterial ECs, with more pronounced effects in female mice.

### Estrogen reduces endothelial *CAV1* expression and LDL uptake, which is not observed after *CNR1* silencing

To clarify a possible influence of female sex hormones on CB1 signaling, we sorted aortic ECs from female and male C*nr1*^*EC-WT*^ mice and observed higher *Cnr1* expression levels in females (Supplementary Fig. S13a). In vitro studies have shown that estrogen induces CB1 and CAV1 expression in cancer cells or smooth muscle cells, respectively, and that the estrogen receptor ESR1 may interact with CAV1, which potentiates ESR1 signaling^43–46^. At the mRNA level, we observed a dose-dependent increase of *CNR1* and *CAV1* expression in HAECs treated with the estrogen receptor ligand estradiol (E2, Supplementary Fig. 13b, c). We subsequently assessed the impact of E2 on CAV1 protein expression and LDL uptake in HAECs transfected with scrambled (*siScr*) or *CNR1* silencing RNA (*siCNR1*), respectively. In *siScr*-treated HAECs with preserved CB1 expression, E2 significantly reduced CAV1 and LDL uptake, which was also observed when silencing *CNR1*, compared to the *siScr*-vehicle group. However, no further decrease in CAV1 expression and LDL uptake was observed in response to E2 silencing *CNR1* (Supplementary Fig. 13d–f), suggesting that estrogen and endothelial CB1 signaling interfere with the same cellular pathway in endothelial cells.

## Discussion

In this study, we provide evidence that CB1 is expressed by human plaque ECs and is upregulated by atherogenic shear stress. We demonstrated that the loss of endothelial CB1 affects vascular tone and reverses endothelial dysfunction after atherogenic diet feeding. Notably, we found that the absence of endothelial CB1 reduces arterial LDL infiltration and improves metabolic parameters in brown and white adipose tissue and liver. In addition, the peripheral CB1 antagonist JD5037 provided therapeutic benefit by limiting plaque progression and endothelial inflammation. These findings reveal a pivotal role for endothelial CB1 as a central regulator linking biomechanical and inflammatory pathways with LDL uptake by the endothelial layer. Thus, antagonizing peripheral CB1 signaling may reduce cholesterol uptake in the arterial wall, thereby contributing to pleiotropic beneficial effects of CB1 blocking in atherosclerosis and related metabolic alterations. In particular, previous experimental studies using peripheral CB1 antagonists have shown reductions in body weight, improved glucose tolerance, decreased hepatic steatosis, and enhanced thermogenesis^15–17,47,48^. In this context, a recent study has further highlighted the relevance of CB1 signaling in gut sensory neurons in regulating energy homeostasis^49^.

We determined the expression of CB1 in human and mouse atherosclerotic vessels at the mRNA level due to the lack of specific antibodies to detect CB1 at the protein level^12^. In human plaque single-cell RNA sequencing data from patients who underwent endarterectomy^20,23^, we detected *CNR1* primarily in ECs and B cells, probably because the sensitivity of the analysis was insufficient to detect *CNR1* expressed by other plaque cell types. Remarkably, we found that endothelial CB1 expression is regulated by shear stress, revealing increased receptor expression in atheroprone regions of mouse aortas and in HAoECs subjected to OSS. Whether endothelial CB1 itself may serve as a mechanosensitive receptor on the cell surface, responding to changes in shear stress patterns, remains unknown. It is also conceivable that *CNR1* is a downstream target of mechanosensitive transcription factors, which requires further investigation. Interestingly, the GO analysis of the transcriptomic profiling of murine aortic ECs indicates that CB1 signaling is associated with the regulation of endothelial membrane raft and actin filament cellular components, which are involved in mechanosensing^3^. It is possible that endothelial mechanosensing is affected in the absence of endothelial CB1 due to transcriptional regulation of mechanosensors. This may explain the observed differences in endothelial cell morphology in C*nr1*^*EC-KO*^ aortas. Because shear stress response and changes in EC alignment depend on both the extracellular fibronectin matrix and the intracellular cytoskeletal F-actin^50^, it is conceivable that endothelial CB1 affects endothelial morphology in response to blood flow through regulation of actin filament signaling. Furthermore, dynamic changes of actin filaments are closely linked to endothelial junctional VE-cadherin remodeling^51^. Therefore, we can speculate that the changes in aortic permeability in the absence of endothelial CB1 may be due to alterations in actin filament signaling. The exact molecular mechanisms underlying this regulation remain to be elucidated.

CB1 signaling has been previously linked to the regulation of vascular tone, in particular in hypertensive conditions^52^. More recently, enhanced vasorelaxant responses were shown in *Cnr1*^*-/-*^ mice compared to corresponding WT controls^53^. In hypercholesterolemic mice subjected to WD, vascular dysfunction was attenuated in *Ldlr*^*-/-*^*Cnr1*^*-/-*^ mice compared to *Ldlr*^*-/-*^ controls^54^. *Ldlr*^*-/-*^*Cnr1*^*-/-*^ mice had a reduced systolic and diastolic blood pressure and were protected against WD-induced blood pressure increases and reductions in endothelium-dependent vasorelaxation.

Here, we found that the absence of endothelial CB1 increases peak flow velocity in atheroprone sites of the aorta in baseline conditions, thereby sharing a similar phenotype as previously reported in *Cav1*-deficient mice^35^. Under WD, the peak flow velocity dropped in atheroprone regions of the aortic arch and aortic roots of *Cnr1*^*EC-KO*^ mice compared to baseline. This was paralleled by a preserved ejection fraction and fractional shortening in *Cnr1*^*EC-KO*^ mice after atherogenic diet feeding, while *Cnr1*^*EC-WT*^ mice developed early signs of heart failure. Together, these findings suggest that endothelial *Cnr1* deficiency affects the regulation of blood flow dynamics, which is likely related to the downregulation of caveolae, known to play an important role in sensing and transducing hemodynamic changes into biochemical signals to regulate vascular function^55^.

In atherogenic conditions, endothelial *Cnr1* deficiency resulted in a transcriptional downregulation of several proinflammatory markers, including *Il6*, *Ccl2*, *Cxcr12*, and *Ackr3*. The proinflammatory role of endothelial CB1 signaling was functionally confirmed by endothelial monocyte adhesion assays under flow conditions. Deficiency of ACKR3 in arterial ECs has been shown to reduce atherosclerotic lesions by limiting arterial leukocyte recruitment, which was linked to decreased NF-κB activity^56^. GSEA and prediction of the top transcription factors modulated by endothelial C*nr1* deficiency revealed a regulation of the JNK/c-JUN and NF-κB pathways. JNK and NF-κB are key regulators of flow-dependent inflammatory gene expression in ECs in atherosclerosis^57–59^. These findings support that endothelial CB1 serves as an upstream regulator of JNK and NF-κB-dependent inflammatory gene expression, which is supported by previously published in vitro data^60,61^.

Furthermore, we provide evidence that endothelial CB1 regulates caveolae-mediated LDL uptake, which is supported by our GSEA findings linking CB1 to regulation of membrane raft and caveolae. While classical lipid receptors such as LDLR, SRB1, and LOX-1 were unaffected by endothelial C*nr1* deficiency, CAV1 and ALK-1, which are associated with caveolae-mediated LDL transcytosis pathways that are independent of the LDLR, were downregulated at the transcriptional level in C*nr1*^*EC-KO*^ mice. This was associated with a reduced number of apical caveolae on the endothelium. Depletion of endothelial CAV1 or ALK-1 leads to impaired arterial LDL uptake^6,9^, which is in agreement with the reduced plaque lipid content and functional LDL uptake in perfused carotids of *Cnr1*^EC-KO^ mice. The changes in endothelial CAV1 expression and LDL uptake by CB1 agonism or antagonism under flow culture conditions support a direct CB1-dependent regulation. In support of this, silencing of the ALK1-encoding gene *ACVRL1* blunted the CB1 agonist-induced upregulation of LDL uptake. It should be acknowledged that only the initial step of subendothelial lipid deposition was assessed in our study, while we did not directly measure lipid transcytosis. Thus, the observed changes in caveolae-dependent LDL uptake and plaque lipid content are merely correlational, even though LDL transcytosis is thought to be rate-limiting in atherosclerosis development^7^. Multiple steps are involved in transcellular transport, and cumulative evidence suggests that caveolar transcytosis across endothelial cells is the dominant pathway, which includes caveolae formation, undocking, trafficking, and docking. Activation of CB1, a G protein-coupled receptor, has been reported to cause G_i_ protein-dependent inhibition of adenylate cyclase, thereby reducing intracellular cyclic AMP production. In smooth muscle cells, stimulation of adenylate cyclase with forskolin has been reported to decrease *Cav1* mRNA expression^62^. In addition, activation of PKA, which occurs when cAMP binds to its regulatory subunits^41^, reduced CAV1 expression in Chinese hamster ovary cells^40^. In agreement with a CB1-cAMP-PKA regulatory pathway of CAV1 expression in ECs, we show that antagonism of CB1 increases cAMP levels and decreases CAV1 in HAoECs, whereas inhibiting PKA restored CAV1 expression (Supplementary Fig. 14). Notably, c-JUN/JNK appears among the top transcription factors with binding sites in the *CAV1* gene promoter according to genecards.org.

Remarkably, endothelial *Cnr1* deficiency resulted in a significant improvement of metabolic parameters in mice subjected to WD. The reduced adipose tissue mass and weight gain in *Cnr1*^*EC-KO*^ mice were associated with an upregulation of *Gpihbp1* expression in WAT and BAT, indicating an increased lipoprotein lipase-mediated capillary lipid uptake and lipolysis. Moreover, *Cnr1*^EC-KO^ mice showed upregulated *Prdm16* expression, which is an important transcriptional regulator mediating brown fat differentiation. Other factors may also contribute to the striking metabolic phenotype observed in *Cnr1*^*EC-KO*^ mice, including reduced lipid accumulation, improved glucose metabolism, and hepatic β-oxidation. The precise mechanisms involved in endothelial CB1-dependent regulation of metabolic processes in WAT, BAT and liver deserve to be investigated in more detail in future studies.

Surprisingly, despite improved adipose tissue and liver metabolism, *Cnr1*^*EC-KO*^ mice had more elevated plasma cholesterol levels, as previously reported in *Cav1*-deficient mice^63^. A common phenotype of endothelial *Cav1* and *Cnr1* deficiency is the reduced aortic LDL infiltration and reduced lesion progression^6,36^. The increased plasma cholesterol levels were only observed in *Apoe*^*-/-*^ mice with endothelial *Cnr1* deficiency, but not in *Ldlr*^*-/-*^ mice chronically treated with the peripheral CB1 antagonist, indicating a potential clinical benefit without undesired side effects on plasma cholesterol levels. Nevertheless, the differential effects on cholesterol levels in the two atherosclerosis knockout strains hint at a model- and intervention-dependent effect. A previous study in *Apoe*^*-/-*^ mice treated with the CB1 antagonist rimonabant also reported comparable serum cholesterol levels in vehicle- and antagonist-treated mice^14^, which suggests that the increase in plasma cholesterol might indeed be an endothelial-specific effect of *Cnr1* deficiency. Other studies even reported lower plasma cholesterol levels in *Ldlr*^*-/-*^ mice or APOE*3-Leiden.CETP mice receiving higher doses of rimonabant^13,64^. At a lower dose, rimonabant reduced atherosclerosis development in *Ldlr*^*-/-*^ mice without affecting cholesterol levels^13^. Nevertheless, systemic cholesterol levels should be carefully monitored in future studies further exploiting the therapeutic benefits of peripheral CB1 antagonism.

In the present study, the effects of endothelial *Cnr1* deficiency on atherosclerotic plaque size and endothelial CAV1 expression were more pronounced in female mice than in males, which might be at least partially explained by the stimulatory effects of estrogens on *CNR1* expression. However, this local effect on the endothelium must be viewed in a systemic context, as estrogen has an overall atheroprotective effect^65^. In support of this, a previous in vitro study reported higher LDL transcytosis in human coronary artery ECs from males and postmenopausal females compared to ECs from younger females^66^. Adding estrogen reduced transcytosis, which was due to G-protein-coupled estrogen receptor-dependent downregulation of SR-BI. In agreement with this, our in vitro experiments showed reduced endothelial LDL uptake upon estrogen treatment, associated with decreased CAV1 protein expression. The finding that no further decrease in CAV1 expression and LDL uptake was observed in response to E2 after silencing *CNR1* suggests that estrogen and endothelial CB1 signaling interfere with the same cellular pathway in endothelial cells, but with opposing effects. Literature data further support an interaction between estrogen receptors ERα and ERβ and caveolin in various cell types^67^, suggesting a complex regulatory interplay between CB1-dependent regulation of caveolae and estrogen signaling.

Of note, a recent study reported increased vasorelaxant responses to acetylcholine and estradiol in aortic ring segments of *Cnr1*^*-/-*^ mice compared to corresponding WT mice^53^, which supports a vasculoprotective effect in the absence of CB1 signaling.

Remarkably, our recent findings revealed a sex-specific effect of myeloid *Cnr1* deficiency, with more pronounced atheroprotective effects and reduced macrophage proliferation observed only in male mice^18^. Furthermore, treatment of *Ldlr*^*-/-*^ mice with a peripheral antagonist during early atherogenesis conferred atheroprotection only in male mice, whereas no difference was seen in females, which is opposite to the protective effects of peripheral CB1 antagonism in females at an advanced stage of plaque development. Our previous study focused on the effects of myeloid *Cnr1* deficiency, which were already evident at early time points in male mice. For this reason, we tested the effect of 4 weeks of treatment with the peripheral antagonist JD5037 in the previous study. Effects of myeloid *Cnr1* deficiency were also noted in female mice, but only at the late time point after 16 weeks of diet (i.e., smaller necrotic core size in aortic root plaques and smaller aortic arch plaque size). Thus, our previous study using 8 weeks WD and treatment during the last 4 weeks of diet was too short to observe effects on atherosclerotic plaque development in female mice. As to the underlying mechanisms that contribute to the differential stage-dependent effects in male versus female mice, we showed an involvement of estrogen signaling in the regulation of chemokine receptor surface expression on monocytes, which is relevant for arterial monocyte recruitment, a key mechanism of early atherogenesis. This may explain why *Cnr1* deficiency or CB1 antagonism during atherogenesis has more pronounced effects in males. Even though no difference in plaque size was observed in male mice after 16 weeks of an atherogenic diet and receiving JD5037 during the last 8 weeks of the diet, there was a significant decrease in adhesion molecule expression notable at this advanced stage of plaque development in both male and female mice. Furthermore, we also observed a tendency for lower CAV1 expression in aortic roots of male *Cnr1*^*EC-KO*^ mice (*p* = 0.0649) and significantly reduced LDL uptake by AM281 in male HAoECs. However, CAV1 was not significantly regulated by AM281 in male hAoECs. Therefore, we argue that our previous and the present study clearly highlight that the effect of genetic *Cnr1* deletion or pharmacological antagonism of the receptor shows distinct strength of effects in macrophages versus endothelial cells, which is dependent on the biological sex and may be partially explained by hormonal effects.

In summary, this indicates that sex differences in CB1 signaling are cell type- and disease stage-specific, at least in mice. Independent of the effect of *Cnr1* deficiency, female mice had larger plaque sizes compared to males at both time points (after 4 and 16 weeks WD), which is a common observation in mouse models of atherosclerosis in normal chow and atherogenic diet condition^68^.

Even though it is widely recognized that atherosclerotic vascular disease in humans does not manifest identically in both sexes, most published single-cell transcriptomic studies of human atherosclerosis did not consider sex differences and predominantly analyzed plaques of male patients. To fill this knowledge gap, a recent study performed deep single-cell sequencing of 7 female and 8 male carotid plaques, which revealed sex differences in the subcellular composition of smooth muscle cells, macrophages, and ECs^69^. The associated gene-regulatory networks that were identified include angiogenesis and T cell-mediated cytotoxicity in male ECs, and endothelial-to-mesenchymal transition in females. This new dataset will be a valuable resource for more in-depth investigations of sex differences in future studies.

A limitation of this study is the unavailability of a CB1-specific antibody for detection at the protein level^12^, which restricts the precise localization of CB1 within the endothelial cell membrane and potential interaction partners. Nevertheless, we have provided unprecedented insights into key regulatory functions of CB1 signaling in ECs. Genetic deficiency or pharmacological inhibition of endothelial CB1 signaling conferred an atheroprotective phenotype, which was more pronounced in female mice, with improved metabolic function in males and females, reduced vascular inflammation, and diminished LDL entry into the artery wall. Results from human aortic ECs reinforced the anti-inflammatory signaling associated with *CNR1* silencing or CB1 antagonist treatment, while CB1 activation induced a proinflammatory phenotype, monocyte adhesion, and EC LDL uptake. Lastly, in vitro experiments revealed that estrogen stimulates CB1 and CAV1 signaling in ECs. In conclusion, peripheral CB1 antagonists may hold promise as an effective therapeutic strategy for treating atherosclerosis and related metabolic disorders.

## Methods

### Animal model of atherosclerosis

To generate mice with endothelial *Cnr1* deficiency on an atherogenic background, *Cnr1*^*flox/flox*^ mice (kindly provided by Beat Lutz)^25^ were first crossed with *Apoe*^*-/-*^ mice to generate *Apoe*^*-/-*^*Cnr1*^*flox/flox*^ mice*. Apoe*^*-/-*^*Cnr1*^*flox/flox*^ mice were then crossed with *Bmx*^*CreERT*^ mice^26^ to obtain *Apoe*^*-/-*^*Bmx*^*Cre(+/-)*^*Cnr1*^*flox/flox*^ mice (referred to as *Cnr1*^*EC-KO*^). The deletion was induced by intraperitoneal injection (i.p.) of tamoxifen (1 mg per 20 g body weight, dissolved in corn oil) at 8 weeks of age, administered for 5 consecutive days to induce *Bmx*^*CreERT*^ transgene expression for selective Cre recombination in arterial ECs. Tamoxifen was also injected into *Apoe*^*-/-*^*Bmx*^*Cre(+/-)*^ and *Apoe*^*-/-*^*Cnr1*^*flox/flox*^ control mice. Following tamoxifen induction, the mice rested for 10 days before baseline harvest or starting a Western diet (WD) consisting of 21% fat and 0.2% cholesterol (Ssniff, TD88137) for either 4 or 16 weeks. In a separate set of experiments, 10-week-old *Ldlr*^*-/-*^ mice^70^ were first fed 8 weeks of WD to induce atherosclerotic plaque formation. Subsequently, the mice were randomly divided into two groups to receive daily i.p. injections of either JD5037 (3 mg/kg) or vehicle (10% DMSO, 40% PEG300, 5% Tween 80, 45% saline) with continuous WD feeding for a total duration of 16 weeks. All experiments included both male and female mice, with biological sex considered in the analysis. At the study endpoints, mice were anesthetized with ketamine/xylazine, and blood was obtained via cardiac puncture. Heart, aorta, adipose tissue, and liver were harvested after PBS perfusion. Animals were housed in ventilated cages, with 4 to 6 mice per cage. The environment was air-conditioned, with a 12-hour light-dark cycle and a temperature of 23 °C and 60% relative humidity. All animal procedures were approved by the local Ethics committee (District Government of Upper Bavaria; license number: 55.2-1-54-2532-111-13 and 55.2-2532.Vet_02-18-114) and conducted in accordance with the institutional and national guidelines and following the ARRIVE guidelines.

### Permeability assay

Evans blue solution (0.5 %) was prepared in saline and sterilized by filtering. Mice were injected into the tail vein with 200 µl of Evans blue solution and euthanized 30 min post-injection as described above. The entire aorta was collected, fixed with 4% paraformaldehyde (PFA) for 30 min, and positioned on slides for imaging (image settings for Evans blue excitation peaks: 470 nm and 540 nm, with an emission peak at 680 nm). Tilescan z-stacks of the whole aorta were taken with Leica DM6000B microscopes and images analyzed with Leica Application Suite LAS V4.3 software.

### Plasma and liver total cholesterol measurement

Total plasma cholesterol concentrations were measured with a colorimetric assay (CHOD-PAP; Roche) and microplate reader (Infinite F200 PRO, Tecan). Plasma from WD-fed mice was diluted at a 1:9 ratio with 0.9% saline for analysis. Liver tissue (50–70 mg) was homogenized in 500 μl of 0.1% NP-40 in PBS using a Tissue Lyser bead mill (Qiagen) and then centrifuged to remove insoluble material. The supernatant was collected and diluted in 0.1% NP-40 PBS. Liver total cholesterol was normalized to the protein concentration, which was determined via a bicinchoninic acid (BCA) assay (Bio-Rad, USA).

### Lipoprotein profile analysis

For lipoprotein separation, plasma samples from 8 mice per group were pooled (0.2 ml) and subjected to fast performance liquid chromatography (FPLC) gel filtration on two Superose 6 columns connected in series as described previously^71^.

### Endocannabinoid measurement

Lipid extraction from mouse plasma was performed on ice. Samples were thawed on ice and spiked with 10 μL internal standard mix. Subsequently, 100 μL ammonium acetate buffer (0.2 M, pH 4) were added. After extraction with 1 mL methyl tert-butyl ether (MTBE), tubes were thoroughly mixed for 4 min using a Bullet Blender Blue (Next Advance Inc., Averill Park, NY, USA) at speed 6, followed by a centrifugation step (16,000×g, 10 min, 4 °C). Next, 950 μL of the upper MTBE layer was transferred into a clean 1.5 mL Safe-Lock Eppendorf tube. Samples were dried in a SpeedVac (Eppendorf, 45 min, 30 °C) and reconstituted in acetonitrile/methanol (50 μL, 70:30, v/v). The samples were thoroughly mixed for 15 min, followed by a centrifugation step (16,000×g, 4 min, 4 °C) and transferred to an LC–MS vial (9 mm, 1.5 mL, amber screw vial, KG 090188, Screening Devices) with insert (0.1 mL, teardrop with plastic spring, ME 060232, Screening Devices). 5 μL was injected into the LC–MS/MS system. A targeted method covering endocannabinoids and related N-acylethanolamines (NAEs) with slight modifications was applied^72^. A QTRAP 6500+ (AB Sciex, Concord, ON, Canada) coupled to an Exion LC AD (AB Sciex, Concord, ON, Canada). MS/MS experiments were done with a Turbo V source (AB Sciex, Concord, ON, Canada) operated with ESI probe. The separation was performed in a BEH C8 column (50 mm × 2.1 mm, 1.7 μm) from Waters Technologies (Milford, MA, USA) maintained at 40 °C, with the flow rate at 0.4 mL/min. The mobile phase consisted of 2 mM HCOONH4, 10 mM formic acid in water (A), ACN (B), and IPA (C). The gradient was the following: starting conditions 20% B and 20% C; increase of B from 20% to 40% between 1 min and 2 min; maintaining B at 40% and C at 20% between 2 min and 7 min; increase of C from 20% to 50% between 7 min and 8 min; maintaining B at 40% and C at 50% between 8 min and 10 min; returning to initial conditions at 10.5 min and re-equilibration for 1.5 min. The triple quadrupole mass spectrometer operated in polarity switching mode, and all analytes were monitored in dMRM mode. Data were acquired using Sciex OS Software V2.0.0.45330 (AB Sciex). Assigned MRM peaks from the acquired data were integrated using SCIEX OS (version 2.1.6) Software, and signals were corrected using proper internal standards. Blank effects for each analyte were checked by comparing proc blank samples to quality control (QC) samples. The precision and reproducibility of the analytical process were checked using the relative standard deviations (RSDs) of the QCs.

### Glucose tolerance test

Mice underwent a 6-hour fasting period with unrestricted access to water prior to i.p. injection of glucose (2 g/kg). Blood samples were collected from the caudal vein to measure plasma glucose levels at specific time intervals (0, 15, 30, 60, and 120 min) using a glucometer (Accu-Chek, Mannheim, Germany).

### Serial echocardiographic assessment

Transthoracic echocardiography was performed with the Vevo® 3100 Imaging System (FUJIFILM VisualSonics; Toronto, Canada) using the MX550 transducer (25-55 MHz). Mice were initially anesthetized with 4% isoflurane supplemented with oxygen, which was reduced to 2-3% during image acquisition. Peak aortic velocities were obtained from the color Doppler-mode aortic arch view. Systolic and diastolic cardiac functions were analyzed in M-mode of the left ventricular parasternal long-axis view. Image analysis and calculations were done using the VevoLAB Version 5.7.0 (FUJIFILM VisualSonics, Toronto, Canada).

### Histology and immunofluorescence of atherosclerotic plaques

Atherosclerotic lesion sizes were analyzed in aortic root cryosections, aortic arch paraffin sections, and *en face* prepared aortas. Mouse hearts were isolated after perfusion with PBS and embedded in Tissue-Tek O.C.T. compound (Sakura) and frozen for cutting into 5 µm cross-sections. Lesion size within aortic roots was quantified after Oil-Red-O (ORO) staining using 8 sections per heart, separated by 50 μm from each other. Aortic arches were fixed overnight in 1% paraformaldehyde (PFA) and embedded in paraffin for longitudinal sectioning (4 µm). Lesion size was quantified after H&E staining, and the average plaque size was calculated from 3-4 sections per arch separated by 40 μm from each other. For *en face* analysis of plaques in the aortic arch and descending aortas, the vessels were fixed overnight in 1% PFA and carefully opened and pinned on black rubber plates for imaging.

Aortic root cryosections were also used for plaque composition analysis, using 3-4 sections per mouse heart for quantification. For assessing macrophage content, acetone-fixed sections were incubated overnight with an antibody against CD68 at 4 °C, followed by anti-rat-AF488 for 1 hour at room temperature (Supplementary Tables 1, 2). Lipid droplets were stained with Nile Red (N3013, Sigma-Aldrich) for 5 minutes, followed by nuclear -staining with Hoechst 33342 for 5 minutes. Plaque collagen and necrotic core content were assessed by Masson’s trichrome staining (stain kit Sigma HT15) in accordance with the guidelines for experimental atherosclerosis studies by the AHA^73^. For CAV1 detection, aortic root cryosections were fixed with 4% PFA, permeabilized with 0.1% Triton X-100 in PBS, blocked for 1 hour and then incubated overnight with anti-CAV1 and anti-CD31 antibodies at 4 °C, followed by anti-rat-AF488 and anti-rabbit-AF647 for 1 hour at room temperature. Corresponding isotypes were included as negative staining controls (Supplementary Table 3). Nuclei were stained with Hoechst 33342 for 5 min. Images for CAV1 detection were taken with a Leica SP8 3 X confocal microscope (Leica), and images were digitized with constant exposure time, gain, and offset. Results were expressed as positive staining area (µm^2^) normalized to the length of the endothelial cell layer (µm) analyzed with the Leica Application Suite LAS V4.3 software. Images of *en face* prepared vessels for plaque quantification were taken with a Leica M205 FCA microscope equipped with a 2.5x objective. All other images were taken with a fluorescence microscope (DM6000B) connected to a monochrome digital camera (DFC365FX, Leica) or connected to a bright-field digital camera (DMC6200, Leica) equipped with Thunder technology for computational clearing and analyzed with the Leica Application Suite LAS V4.3 software. All plaque data are expressed as average per section and mouse heart.

### ICAM1, VCAM1 and CAV1 staining in aortic arch

Aortic arches were fixed in 1% PFA overnight and embedded in paraffin for longitudinal sectioning (5 µm). The tissue sections were first deparaffinized and subjected to antigen retrieval. Tissue sections were then incubated overnight at 4 °C with primary antibodies targeting CAV1, ICAM1, and vWF, or VCAM1 and vWF (Supplementary Tables 1–3) after 30 min blocking at RT, followed by corresponding Alexa Fluor secondary antibodies for 30 min at RT. Slides were mounted with Vectashield mounting media with DAPI. Quantification was conducted on 4 sections per animal, with a 50 µm interval between each section imaged by a Leica Thunder DM6000B microscope at 20x magnification. To determine the percentage of ICAM1/VCAM1/CAV1-positive ECs, the number of ICAM1/VCAM1/CAV1-positive cells was calculated and normalized to endothelial cell numbers using LAS V4.3 software (Leica).

### Immunofluorescence staining of brown adipose tissue

Brown adipose tissue (BAT) was fixed overnight in 4% PFA and embedded in paraffin for longitudinal sectioning (4 µm). The paraffin sections were first deparaffinized and subjected to antigen retrieval. Tissue sections were then incubated overnight at 4 °C with primary antibody against GPIHBP1 and vWF after 30 min blocking at RT, followed by Cy3 donkey anti-rabbit and Cy5 anti-sheep secondary antibody staining for 30 min at RT (Supplementary Tables 1-3). Slides were mounted with Vectashield mounting media with DAPI. Images were taken with a Leica Thunder DM6000B microscope (Leica) and quantified using LAS V4.3 software (Leica). Five images were captured per section, and 3-4 sections per mouse were quantified, with the results presented as the mean value per animal.

### *En face* immunofluorescence staining of thoracic aortas

Prior to harvest, the thoracic aortas were perfused with 20 ml pre-cooled PBS containing 20% FBS and 4% PFA. Subsequently, the vessels were carefully opened and transferred to a 12-well plate containing 4% PFA for 20 min fixation. Whole-mount immunofluorescence staining was performed based on a published protocol^74^. The vessels were permeabilized with 0.1% Triton X-100 in PBS, washed, and blocked with PBS containing 1% horse serum and 1% BSA for 1 hour at room temperature. Aortas were incubated overnight with primary antibodies against CD144 and ICAM1 on a rocking platform at 4 °C, followed by 1 hour incubation with donkey anti-rat and goat anti-Armenian hamster Alexa Fluor secondary antibodies (Supplementary Tables 1-3). Aortas were mounted with Vectashield mounting media with DAPI and imaged using a confocal microscope (TCS-SP5, Leica) at 488 and 550 nm, respectively. Endothelial cells were identified as CD144 positive under the same settings. Atheroprone and atheroprotective regions were chosen for high-magnification imaging after low-magnification tile scanning of the entire aorta. The mean fluorescence intensity (MFI) of maximum projections of images was analyzed using LAS X Office image processing software and ImageJ.

### Aorta and arch *en face* preparation and lesion quantification

Aortic arch and abdominal aortas were excised following euthanasia and heart perfusion with 10 mL of pre-cooled PBS. The isolated vessels were placed in 1.5 mL Eppendorf tubes with 1% PFA solution overnight. After fixation, the vessels were transferred to a Petri dish containing PBS. Sequentially, the vessels were carefully opened under a dissecting microscope (Leica), with the removal of fat and connective tissue according to Pei-Yu Chen et al.^75^. Subsequently, the opened aorta and arch were fixed with minutiae pins on a rubber plate. Imaging of the vessels was subsequently performed with a Leica M205 FCA microscope equipped with a 2.5x objective. For lesion quantification, the ImageJ software was employed to delineate lesion areas using the “polygon selection” tool. Subsequently, the selected areas underwent analysis within the Region of Interest (ROI) management function through measurement and recording. The relative plaque percentage was then determined by normalizing the plaque area to the vessel area.

### Fluorescence in situ hybridization

Endothelial *Cnr1* detection was performed by in situ hybridization combined with immunostaining employing the viewRNA cell plus kit (Thermo Fisher)^76^. Arch and thoracic aortae *en face* prepared thoracic aortas of *Apoe*^*-/-*^ mice were fixed in Paxgene for 1 hour and 30 minutes and en face prepared for tissue stabilization in a stabilized solution overnight at 4 °C. The entire procedure was performed in RNase-free conditions and using an RNase inhibitor cocktail alongside the hybridization steps. Tissues were incubated with a custom probe designed for murine *Cnr1* (VB6-17606, Affymetrix) and incubated at 40 ± 1 °C for 2 hours for the target probe hybridization process. The hybridization probe was diluted in probe set diluent to achieve a final concentration of 5 μg/ml. Subsequently, the tissues were washed and incubated with the pre-amplifier mixture at 40 ± 1 °C for 90 min, and then exposed to the amplifier mixture for an additional 1 hour to enhance the signal. After the hybridization process, tissues were incubated with appropriate fluorescently labeled probes (Type 6) for 1 hour. Tissues underwent a thorough wash followed by 30 min of incubation with a fixation/permeabilization buffer, another wash, and a 1-h incubation with blocking buffer at room temperature. Subsequently, they were stained with anti-CD31 antibody overnight at 4 °C in a humid chamber, followed by a 1 hour incubation with AlexaFluor488 anti-rat secondary antibody at room temperature. Tissues were transferred to glass slides after final washing and mounted with Vectashield mounting media with DAPI.

Aortic root cryosections were collected on RNAse-free slides (pre-treated with RNAse ZAP). The cryosections were fixed in 4% PFA for 5 min, followed by treatment with pre-warmed 10 μg/ml proteinase K (diluted in PBS) for 5 min at RT. Subsequently, post-fixation was carried out with 100% ethanol for 1 min. The prepared slides were then mounted with SecureSeal™ hybridization chambers. The slides were then placed on the in-situ adapter of an Eppendorf Mastercycler machine and incubated at 40 ± 1 °C following the same hybridization procedure as described above. Afterwards, the chambers were removed, washed, and stained with anti-CD31 and Hoechst 33342 as described above. Images were taken using a confocal microscope TCS-SP5. An average of 5-10 images per section and per vessel were acquired and subsequently quantified using ImageJ software.

### Ex vivo imaging of lipid uptake in perfused carotid arteries

Endothelial Dil-LDL (3,3’-dioctadecylindocine-low density lipoprotein) uptake was assessed in murine carotid arteries of *Cnr1*^*EC-WT*^ and *Cnr1*^*EC-KO*^ mice (*n* = 5–7) mounted in perfusion chambers as previously described^34,77^. The vessels were first incubated with anti-CD31 (Supplementary Table 4) under a static pressure of 80 mmHg for 10 min at 37 °C. After washing, DiL-LDL was loaded onto the arteries and incubated for 90 min under 80 mmHg at 37 °C. Following the removal of unbound antibodies and DiL-LDL through artery flushing, tissues were imaged using a Leica SP5 IIMP two-photon laser scanning microscope coupled to a Ti:sapphire laser (Spectra Physics MaiTai DeepSee) tuned at 800 nm. A 20× NA1.00 (Leica) water dipping objective was utilized, and spectral detection employed internal Hybrid Diode detectors tuned for optimal contrast between various targets while maintaining sufficient fluorescence signal intensity from the arterial wall. Three-dimensional image processing and quantification of DiL-LDL distribution per endothelial cell were performed using Leica LASX 3.11 software, utilizing 3D analyser and lightning plugins.

### Transmission electron microscopy (TEM)

Mice were perfusion fixed in EM-grade 4% paraformaldehyde (Science Services), 2.5% glutaraldehyde (Science Services), 2 mM calcium chloride in 0.1 M sodium cacodylate buffer, pH 7.4 (Science Services), pre-warmed to 37 °C. After isolation of the aortic arch, it was immersion fixed in the same fixative overnight, and the aortic arch was prepared in buffer and post-fixed overnight. Employing a reduced osmium thiocarbohydrazide osmium (rOTO) en bloc staining protocol^78^, the samples underwent a sequential series of treatments. This process involved post-fixation in a solution containing 2% osmium tetroxide (EMSScience Services), 1.5% potassium ferricyanide (Sigma-Aldrich) in 0.1 M sodium cacodylate buffer (pH 7.4; Science Services). The staining was intensified through a 45 -min incubation at 40 °C with 1% thiocarbohydrazide (Sigma-Aldrich). Subsequently, the tissue underwent rinsing in water and immersion in a 2% aqueous solution of osmium tetroxide. After another wash, an additional level of contrast was achieved through an overnight incubation in a 1% aqueous solution of uranyl acetate at 4 °C, followed by an extra two hours' incubation at 50 °C. In preparation for subsequent processing steps, the samples underwent dehydration through a series of increasing ethanol concentrations and were infiltrated with LX112 (LADD). The aortic arch was embedded using a gelatine capsule and cured at 60 °C for two days. The region of interest was trimmed (TRIM2, Leica) and sections generated on an ultramicrotome (UC7, Leica) at a nominal thickness of 80 nm using a 35° ultra-diamond knife (Diatome). Ultrathin sections were deposited onto formvar-coated copper grids (Plano) without post-contrasting. TEM micrographs were acquired on a JEM 1400plus (JEOL) equipped with an XF416 camera (TVIPS) and the EM-Menu software (TVIPS). Ultrastructural image analysis of endothelial caveolae was performed using ImageJ by normalizing apical caveolae to the plasma membrane length.

### Flow cytometry analysis of blood leukocytes

Freshly collected whole blood (50 µl) was transferred to ice-cold FACS tubes and subjected to red blood cell lysis with ammonium-chloride-potassium (NH_4_Cl (8,024 mg/l), KHCO_3_ (1001 mg/l), EDTA.Na_2_·2H_2_O (3,722 mg/l)) buffer for 10 min at RT. Cell suspensions were incubated with an antibody mix (Supplementary Table 5) for 30 minutes at 4 °C in the dark. Then, the cell suspensions were washed with 1 ml of FACS buffer and centrifuged into 300–500 µl FACS buffer based on the cell count. The cells were washed and acquired with a BD FACSCanto II flow cytometer (BD Biosciences) and analyzed with FlowJo v10.2 software (Tree Star, Inc). Cells were gated as singlets, live, and CD45^+^CD11b^+^ myeloid subsets and further gated as CD115^+^Ly6G^-^ (monocytes) and CD115^-^Ly6G^+^ (neutrophils).

### Flow cytometry sorting of aortic and adipose tissue endothelial cells

Murine aortas spanning from the aortic arch to the iliac bifurcation were isolated after perfusion with PBS and digested with collagenase IV and DNase I (Supplementary Table 6) at 37 °C for 40 min. The interscapular brown adipose tissue (BAT) was collected, cut into small pieces and digested with collagenase I, collagenase XI, DNase I, and hyaluronuclease (Supplementary Table 7) at 37 °C for 30 min. Subsequently, the digested tissues were washed and filtered through a 30-μm cell strainer (Cell-Trics, Partec). The resulting single-cell suspensions were stained with antibody cocktails (Supplementary Table 5) and sorted using a BD FACS Aria III Cell Sorter (BD Biosciences), gated as live CD45^low^CD31^high^CD107a^high^ aortic endothelial cells or live CD45^-^CD31^+^ as BAT endothelial cells, respectively. The sorted cells were deep-frozen in 2× TCL buffer (Qiagen) plus 1% β-mercaptoethanol at -80 °C until subsequent RNA extraction and library preparation.

### RNA sequencing

RNA sequencing of 10,000 sorted aortic and BAT ECs isolated from *Cnr1*^*EC-WT*^ or *Cnr1*^*EC-KO*^ mice after 4 weeks WD (*n* = 6) was performed using the prime-seq protocol that can be found on protocols.io (dx.doi.org/10.17504/protocols.io.s9veh66)^79^. For differential gene expression analysis, DESeq2 (Version 1.37.4), a Bioconductor package implemented in R version 4.2.0 (2022-04-22), was utilized. The analysis was executed on an Ubuntu 20.04.3 LTS system, employing the negative binomial distribution for the necessary computations. Initially, size factors and sample dispersion were estimated, followed by utilizing Negative Binomial GLM fitting and computing Wald statistics using DESeq2. DESeq2 was applied to identify differentially expressed genes (DEGs) based on the criterion of an adjusted *P*-value below 0.10^80,81^. The volcano plot was created using the ggplot2 package (Version 3.4.0). Gene Ontology (GO) enrichment analysis was conducted using the enrichplot (Version 1.17.2) Bioconductor package^82^, employing DEGs filtered by an adjusted *P* value < 0.10. The CHEA3 web server was employed to predict the top 15 transcription factors (TFs) regulating DEGs^83^. Gene Set Enrichment Analysis (GSEA) was performed using GSEA software (version 4.3.2), which was tailored for the Windows operating system. The analysis utilized curated gene sets (M2), ontology gene sets (M5) and mouse-ortholog hallmark gene sets sourced from mouse collections^84^. Pathways were considered significant according to predefined criteria, encompassing a normalized enrichment score below -1 or above 1, a false discovery rate below 0.25, and a nominal *P* value less than 0.05^85^. The bulk RNA sequencing dataset is accessible under GEO accession number GSE260826.

### Analysis of human single-cell RNA sequencing data

Single-cell expression data were retrieved from the datasets generated as part of Bashore et al., available via Gene Expression Omnibus (GEO, accession code: GSE253904)^20^, and analyzed as previously^20,86^. Briefly, we identified and removed doublets using Doublet Detection (v4.2), which was run separately on each sample (1468 doublets in total). We then jointly analyzed the samples using scanpy (v1.9.5). Out of 80,474 cells, we filtered out (a) genes expressed in <3 cells, (b) cells with <200 or >40,000 total counts, (c) cells with >20% counts from mitochondrial genes, and (d) cells with >6000 genes. Filtered counts were normalized and log1p transformed, and the 3137 genes identified as highly variable genes (HVGs) were used for the principal component analysis (PCA). The first 30 principal components (PCs) were used to construct the neighborhood graph and visualize the data in the uniform manifold approximation and projection (UMAP). The retained 73,833 cells were grouped into 21 clusters using the Leiden algorithm with a 0.5 resolution. A cluster of 33 cells expressing platelet markers was excluded from downstream analysis. Cluster annotation was based on established cell markers in the literature and summarized in Supplementary Fig 1A. To enhance sensitivity, we applied a zero-preserving imputation method (Adaptively thresholded Low-Rank Approximation, ALRA)^87^ through SeuratWrappers (v0.3.5, R v4.4.1).

### Quantitative real-time PCR

Total isolated RNA from cells (RNeasy Plus Mini Kit, Qiagen) or tissues (peqGOLD, 13-6834-02, VWR Life Science) was reverse transcribed (PrimeScript RT reagent kit, TaKaRa) to cDNA. Real-time qPCR was performed with the QuantStudio™ 6 Pro Real-Time PCR System (ThermoFisher) using the GoTaq Probe qPCR Master Mix (Promega). Primers and probes were purchased from Life Technologies (Supplementary Tables 8-9). Hypoxanthine-guanine-phosphoribosyltransferase (*Hprt*) was used as the endogenous control. For BAT samples, ubiquitin C (*Ubc*), was utilized as an endogenous reference. Target gene expression was normalized to the endogenous control and presented as a fold change relative to the control group.

### Droplet digital PCR

The QX200 Droplet Digital PCR (ddPCR™) system (BioRad) was used for ddPCR analysis. The master mix contained ddPCR supermix (900 nM), primers and probes mix (250 nM), (Supplementary Table 9; IDT Integrated DNA Technologies) for a final 20 μl volume of reaction mix. Droplets were generated by combining 20 µl of the reaction mix with 70 µl of droplet generation oil for probes in the QX200 droplet generator (BioRad). The resulting droplet solution was transferred to a 96-well PCR plate, the cycling process was carried out, and analyzed with QuantaSoft software (BioRad).

### Human primary cell culture and transfection

Human Primary Aortic Endothelial Cells (HAoECs) from a 61-year-old female donor (458Z035.1; C-12271; PromoCell) or a 50-year-old male donor (434Z005.1; C-12271; PromoCell) were seeded on 0.2% gelatin-coated plates or ibidi chambers in Endothelial Cell Growth Medium (ECGM; C-22010, PromoCell) supplemented with 1% Penicillin-Streptomycin at 37 °C in a humidified atmosphere containing 5% CO2. Cells were used at passages 4 to 6 for experiments. Off-target-plus siRNAs (Smartpool) against human *CNR1* (*siCNR1*), *ACVRL1 (siALK1)*, and scrambled siRNA (*siScr*) were obtained from Dharmacon (Supplementary Table 10; Horizon, UK). HAoECs were transfected with 20 nM siRNA utilizing RNAiMax (Invitrogen) and analyzed 24 hours or 48 hours after transfection. For silencing experiments, HAoECs were exposed to flow 24 h after transfection. Subsequently, in vitro LDL uptake was performed.

### Shear stress assays

An ibidi pump system was used to impose laminar (LSS) or oscillatory shear stress (OSS) on confluent monolayers of HAoECs seeded into flow chambers as described^88,89^. HAoECs were exposed to LSS (10 dyne/cm²) or OSS (3 dyne/cm²) in ECGM for 24 hours.

### Monocyte adhesion assay

HAoECs were incubated with CB1 agonist ACEA (1 µM) or vehicle control under LSS at 10 dyne/cm² in ibidi chambers for 24 hours. Subsequently, the cells were stimulated with 5 ng/ml TNF-α (R&D) overnight without the application of shear stress. Then, monocytes (1 × 10^5^ THP-1 /ml) labeled with 0.5 µM calcein (Invitrogen) were added to the flow system. THP-1 and HAoECs were co-cultured under LSS at 37 °C for 3 hours. Afterwards, the chambers were disconnected from the perfusion system, washed with PBS, and imaged with an inverted microscope Leica DMi8 S Platform with the Tilescan function) at 20x magnification. At least 10–15 different random views per condition were selected to quantify the number of adherent THP-1 cells using ImageJ software.

### In vitro DiI-LDL uptake

HAoECs were first incubated with CB1 agonist ACEA (1 µM) or vehicle control under LSS for 24 hours. Afterwards, 1 µg/ml Dil-LDL was added in medium without supplements and incubated under static conditions at 37 C° for 1.5 hours. Cells were washed, fixed with 2% PFA, and permeabilized with 0.1% Triton X-100 for 10 min at room temperature. The chambers were mounted with ibidi Mounting Medium containing DAPI and imaged using an inverted microscope (DMi8 S Platform, Leica) with a 20x objective. The mean fluorescence intensity of Dil-LDL (550 nm/564 nm) was quantified using LAS V4.3 software (Leica).

In other experiments, AM281 was added 20 min before exposing HAoECs to OSS. The PKA inhibitor KT5720 (1 µM) was added 1 hour prior to AM281 treatment. After 24 hours of culture in OSS conditions, the cells were incubated with 1 µg/ml Dil-LDL for 30 min at 37 °C in static conditions. Dil-LDL uptake by cells transfected with *siScr* or *siCNR1* was performed 24 hours after transfection and 12 hours treatment with 10 nM estradiol (E2) in static conditions. The cells were subsequently fixed and permeabilized with 0.1% Triton x-100, followed by incubation with a primary antibody against CAV1 after 1 hour of blocking at room temperature. The next day, the cells were washed and incubated with Alexa Fluor 647 anti-rabbit secondary antibody for 1 hour. The cells were mounted with ibidi Mounting Medium containing DAPI and imaged using an inverted microscope (DMi8 S Platform, Leica) with a 63x objective. Mean Dil-LDL and CAV1 fluorescence intensity were quantified using LAS V4.3 software (Leica) using 5-10 images per condition.

### ROS and nuclear phospho-c-Jun detection

HAoECs were transfected with either 20 nM *CNR1* or scrambled siRNA as described above. After 24 hours of transfection, the cells were stimulated with 10 ng/ml TNFα for 30 min. Subsequently, the HAoECs were washed and incubated with 1 µM DHR-123 (Dihydrorhodamine) for 20 min at 37 °C to detect ROS production. The cells were then collected after trypsinization, centrifuged, and resuspended in FACS buffer for flow cytometry analysis with a BD FACSCanto II flow cytometer (BD Biosciences). Data were analyzed using FlowJo v10.2 software. For detection of nuclear phospho-c-Jun (Cell Signaling), the cells were grown on chamber coverslips (ibidi), treated for 30 min with ACEA, AM281 or vehicle, fixed with 4% PFA and finally immunostained with primary antibody against phospho-c-Jun and secondary with anti-rabbit-AF488. The cells were mounted with ibidi Mounting Medium containing DAPI, and images were taken with a fluorescence microscope (DM6000B) connected to a monochrome digital camera (DFC365FX, Leica) equipped with Thunder technology for computational clearing. At least 5 different area views of images per condition were selected for quantification with the Leica Application Suite LAS V4.3 software.

### Cyclic AMP (cAMP) measurement

To measure intracellular cyclic adenosine monophosphate (cAMP) concentrations, HAoECs seeded in 12-well plates were pretreated with 3-isobutyl-1-methylxanthine (0.5 mM, IBMX, Sigma-Aldrich) for 30 min and then stimulated with CB1 antagonist AM281 or CB1 agonist ACEA for 20 min. Forskolin (3 µM, Sigma) was used as a positive control for assessing maximum cAMP levels. Cells were lysed with 0.1 M HCl and intracellular cAMP levels measured with the Cyclic AMP Select ELISA kit (Cayman Chemical). In other experiments, intracellular cAMP was measured in Flp-In TREx-293 (HEK293) cells (Invitrogen) stably transfected with the human *CNR1* cDNA (Missouri S&T cDNA Resource Center) as well as a luciferase-cAMP reporter plasmid (pGloSensor-20F-vector; Promega). F20 Flp-InT-Rex 293 cells were cultured with DMEM supplemented with 10% fetal calf serum and 1% Pen/Strep and passaged using Trypsin-EDTA (0.05%). After incubation with luciferin-EF (2.5 mM, Promega) at room temperature for 1 hour, cells were stimulated with ACEA or AM281, followed by the addition of forskolin (1 µM). The luminescence signal as a readout for intracellular cAMP levels was recorded in real time using the Tecan Infinite F200 PRO microplate reader.

### Statistics

Statistical analyses were conducted using GraphPad Prism version 10.5 (GraphPad Software, Inc.). Data distribution was assessed using the D’Agostino-Pearson omnibus and the Shapiro-Wilk tests. Data violating the assumption of Gaussian distribution were analyzed by the Mann-Whitney *U* test (two-group comparisons) or the Kruskal-Wallis *H* test with Dunn’s *post hoc* test. For normally distributed data, homogeneity of variance was tested using Levene’s test, and outliers were identified by Tukey’s method. Then, unpaired Student’s *t*-test with Welch correction when appropriate (two-group comparisons), or univariate ANOVA with Tukey *post hoc* test for pairwise comparisons (three or more groups) was performed. In case of multiple comparisons, a false discovery rate (FDR) approach according to Benjamini-Hochberg was used, with a threshold of 5%. In analyses involving two fixed factors, two-way ANOVA with Tukey *post hoc* test for pairwise comparisons was applied. For models involving dependency of observation, we fitted a mixed-effect model using a Restricted Maximum Likelihood method as implemented in Prism to test the difference between genotypes (fixed effect). The assumption of sphericity was verified with Mauchly’s *W* test, and the Greenhouse-Geisser correction was applied in case of violation. Data are presented as means and standard error of means (s.e.m.) unless otherwise stated. Differences were considered statistically significant for a two-tailed *P* value < 0.05.

### Reporting summary

Further information on research design is available in the Nature Portfolio Reporting Summary linked to this article.

## Supplementary information

Supplementary Information

Reporting summary

Transparent Peer Review file

## Source data

Source data

## Data Availability

The RNA sequencing data generated in this study have been deposited in the GEO database under accession code GSE260826. All other data generated in this study are provided in the Source Data file. Source data are provided with this paper.
