# Supplementary material for: Endothelial cannabinoid CB1 receptor deficiency reduces shear stress-induced arterial inflammation and lipid uptake
Source: Nat Commun. 2026 Jul 7;17:5939. doi: 10.1038/s41467-026-75214-2 (PMC13342577; doi:10.1038/s41467-026-75214-2)
Supplement: Supplementary file 1 — Supplementary Information [file 41467_2026_75214_MOESM1_ESM.pdf]

Supplementary Information

Endothelial cannabinoid CB1 receptor deficiency reduces shear stress-induced arterial inflammation and lipid uptake (Chen, Prabhu et al.)

Content:

-Supplementary Figures

-Supplementary Tables

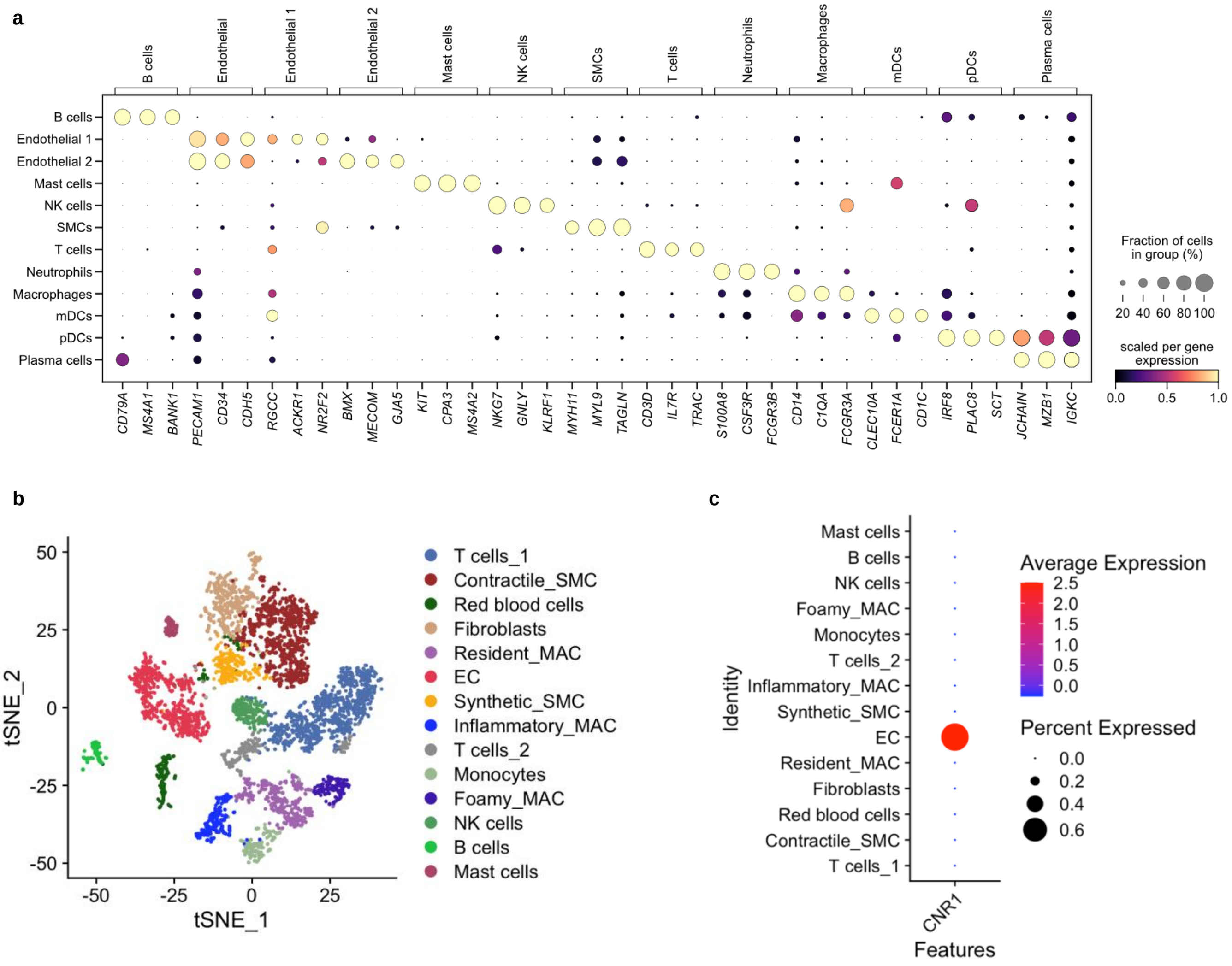

Supplementary Fig. 1. *CNR1* expression in human atherosclerotic carotid plaques. a Dot plot depicting the average expression (color scale) and the proportion of expressing cells (dot size) of cell-type specific marker genes used to define the distinct clusters visualized in Fig. 1a. Single-cell expression data are from human carotid plaque specimens (GEO accession code: GSE253904). **b** t-distributed stochastic neighbor embedding (t-SNE) reveals cell clusters in an independent human carotid plaque single-cell RNA sequencing data set (GSE247238). **c** The dot plot displays the *CNR1* expression within each cell cluster, with the colour gradient indicating the average expression level and the point size reflecting the percentage of expression within each cluster (SMC, smooth muscle cells; MAC, macrophage; EC, endothelial cells; NK cell, natural killer cells).

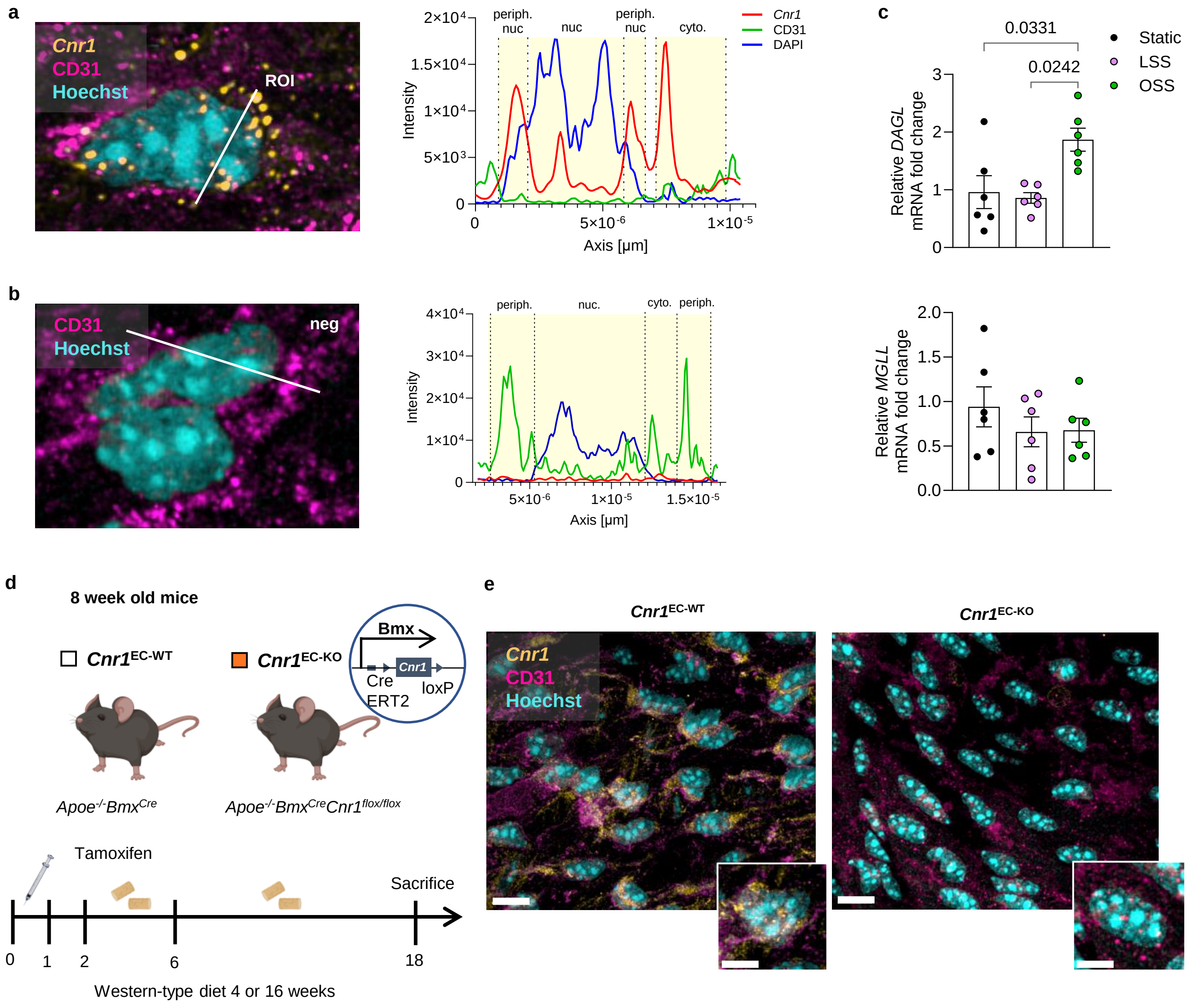

Supplementary Fig. 2. Detection of *Cnr1* and endocannabinoid-related gene expression. a-b *En face in situ* hybridization was performed for *Cnr1* and corresponding negative control in thoracic aortas of 8-week-old *Apoe*$^{-/-}$ mice. The intensity of *Cnr1* (Red), CD31 (Green) and DAPI (Grey) within endothelial cells in the region of interest (ROI) was quantified and using IMARIS, as presented on the right (scale bar, 5 µm). **c** Endocannabinoid 2-arachidonoylglycerol biosynthesis and degradation enzyme *DAGL* and *MGLL* mRNA levels in HAoECs (n=6) after 24 h exposure to static, LSS (10 dyn/cm2) or OSS (3 dyn/cm2) conditions, determined by RT-qPCR. **d** Experimental scheme (Created in BioRender. Prabhu, A. (2026) https://BioRender.com/esv7wyx) 8 week old *Cnr1*$^{EC\text{-}WT}$ and *Cnr1*$^{EC\text{-}KO}$ mice received daily intraperitoneal injections of tamoxifen for 5 days and were subsequently fed with Western diet (WD) for a duration of 4 or 16 weeks. **e** Representative images of in situ hybridization for *Cnr1* (red) combined with CD31 (green) immunostaining for ECs and Hoechst 33342 staining (blue) for nuclei using *en face* prepared aortas of *Cnr1*$^{EC\text{-}WT}$ and *Cnr1*$^{EC\text{-}KO}$ mice after 4 weeks WD. Scale bar: 10 µm (overview) and 2 µm (insert). Data are shown as mean ± s.e.m.; Kruskal-Wallis *H* test with Dunn's *post hoc* (**c**) was applied. Each data point represents one individual human or mouse sample (biological replicate), collected in at least 2 independent experiments.

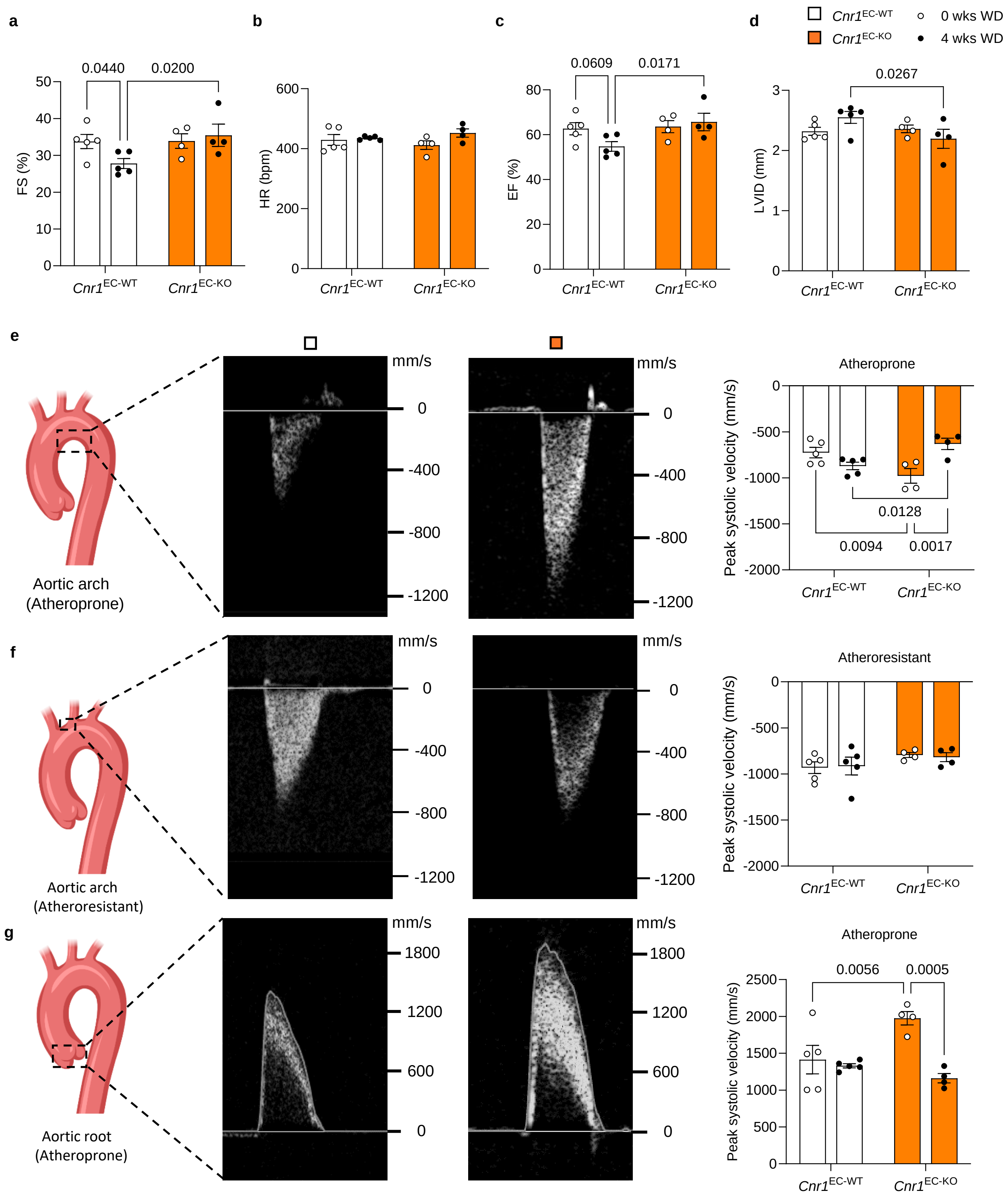

Supplementary Fig. 3. Echocardiographic assessment of cardiac function and aortic flow velocity in *Cnr1*$^{EC\text{-}WT}$ and *Cnr1*$^{EC\text{-}KO}$ female mice. Age-matched female *Cnr1*$^{EC\text{-}WT}$ mice (n=5) and *Cnr1*$^{EC\text{-}KO}$ mice (n=4) were used to assess the cardiac function and blood flow dynamics by serial echocardiography. **a-d** Heart rate (HR), ejection fraction (EF), fractional shortening (FS) and end-diastolic left ventricular internal diameter (LVID) in *Cnr1*$^{EC\text{-}WT}$ and *Cnr1*$^{EC\text{-}KO}$ mice at baseline (0 wks WD) or after 4 weeks of WD. **e-g** Schematic of scan location (Created in BioRender. Prabhu, A. (2026) https://BioRender.com/esv7wyx). Quantification of peak systolic velocity at atheroprone and atheroresistant sites of aortas at baseline or after 4 weeks of WD**.** Data are shown as mean ± s.e.m.; Mixed effect analysis with Fisher's test **a-g** were applied. Each data point represents one individual human or mouse sample (biological replicate), collected in at least 2 independent experiments.

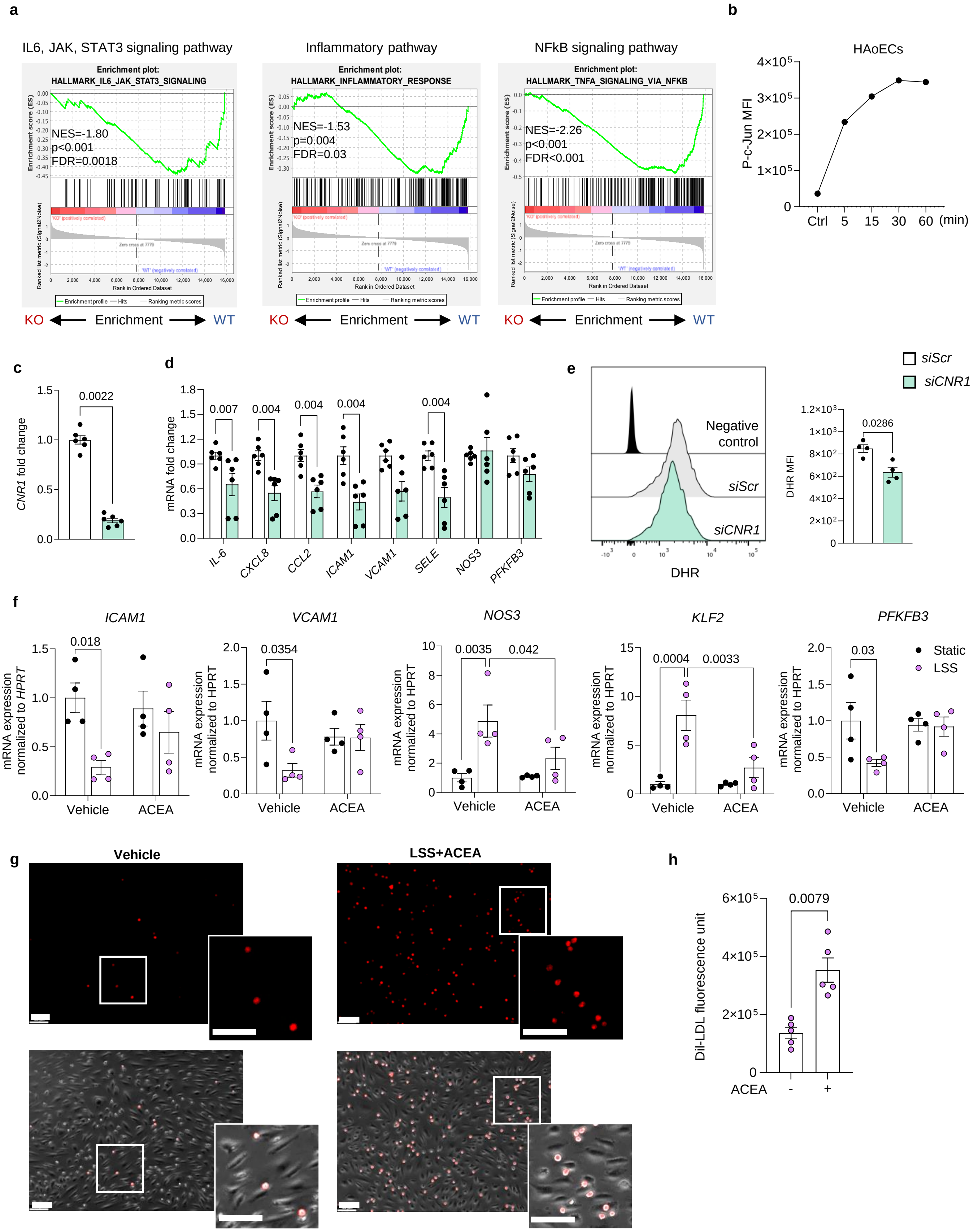

Supplementary Fig. 4. Role of endothelial CB1 in vascular inflammation and monocyte adhesion. a Pathways associated with endothelial CB1-regulated genes (GSEA), based on RNA sequencing data of sorted ECs from female *Cnr1*$^{\text{EC-WT}}$ and *Cnr1*$^{\text{EC-KO}}$ mouse aortas (n=6) after 4 weeks of WD, shown in Fig. 3 a-b. **b** Time course of phospho-c-Jun mean fluorescence intensity (MFI, immunostaining) in TNFα-treated HAoECs from a female donor. (n=1) **c,d** Knockdown efficiency of *CNR1* and pro-inflammatory gene expression 24h after transfection with 20 nM *CNR1* (*siCNR1*) or scrambled siRNA (*siScr*) in female HAoECs (n=4). **e** Flow cytometric analysis of ROS determined by DHR1123 in female HAoECs, pretreated with TNFα (gMFI, geometric MFI; n=4). **f** Expression levels of shear stress related genes assessed by RT-qPCR in HAoECs treated with 1 µM ACEA or vehicle under static or LSS (10 dye/cm^2) for 24 h (n=4). **g** Representative images of THP-1 monocyte adhesion (red) in vehicle- or ACEA- (1 µM) treated HAoECs under LSS. Scale bar, 200 µm (overview) and 100 µm (insert). **h** Adherent THP-1 cells were counted in 10-15 random fields per experiment (n=6). Data are shown as mean ± s.e.m.; Multiple Mann-Whitney *U* (**c, d**), Mann-Whitney *U* (**e, h**) or two-way ANOVA with Sidak *post hoc* (**f**) test were applied. Each data point represents one individual human sample (biological replicate), collected in at least 2 independent experiments.

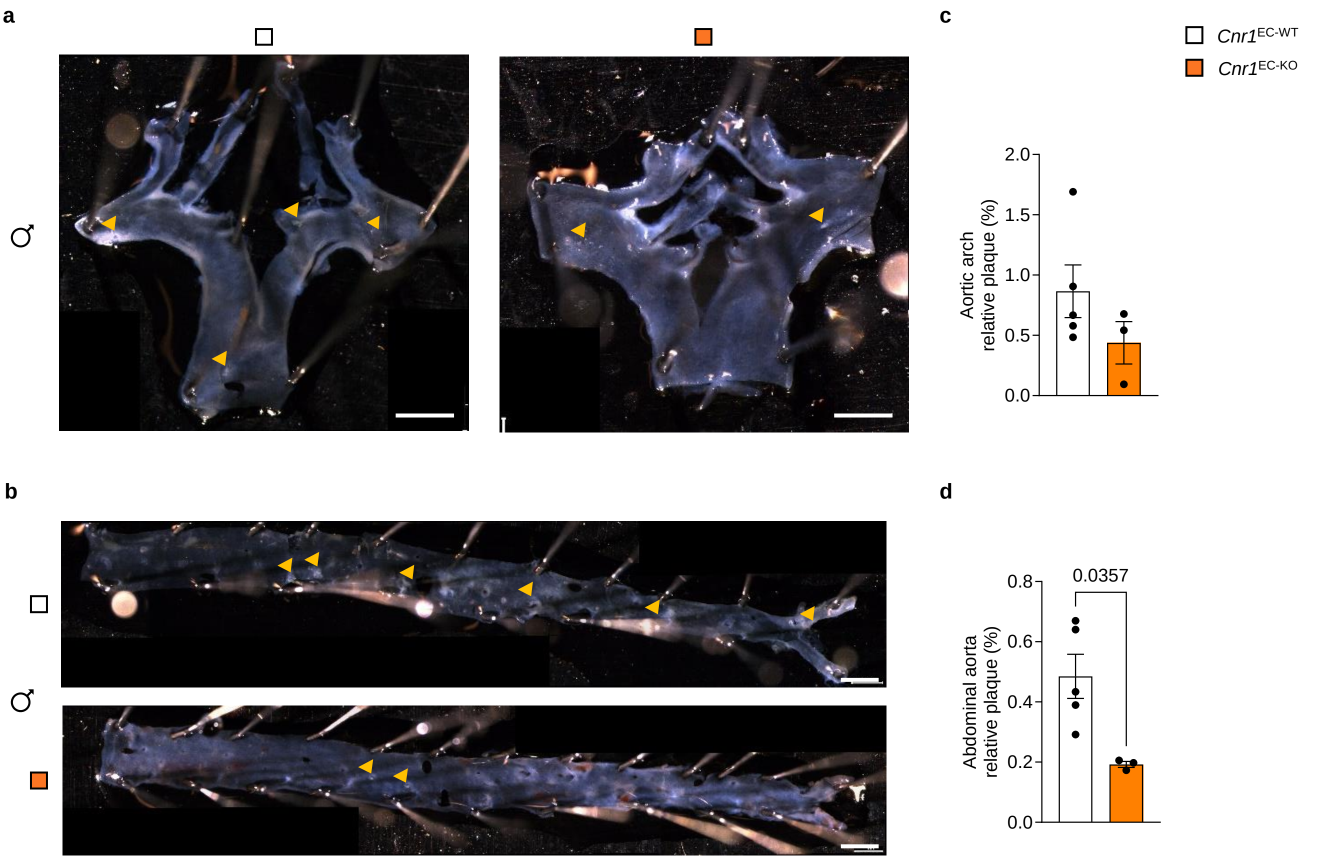

Supplementary Fig. 5. Effect of endothelial *Cnr1* depletion on atherosclerotic plaque in males. a-b Representative images and analysis of arch **a** and descending aorta lesion area **b** normalized to vessel area from male *Cnr1^EC-WT^* (n=5) and *Cnr1^EC-KO^* mice (n=3) after 4 weeks of WD. Scale bar, 1 mm. **c-d** Quantification of aortic arch **c** and descending aorta **d** relative lesion size. Data are shown as mean ± s.e.m.; Mann-Whitney *U* (**c, d**) test was applied. Each data point represents one individual mouse sample (biological replicate), collected in at least 2 independent experiments.

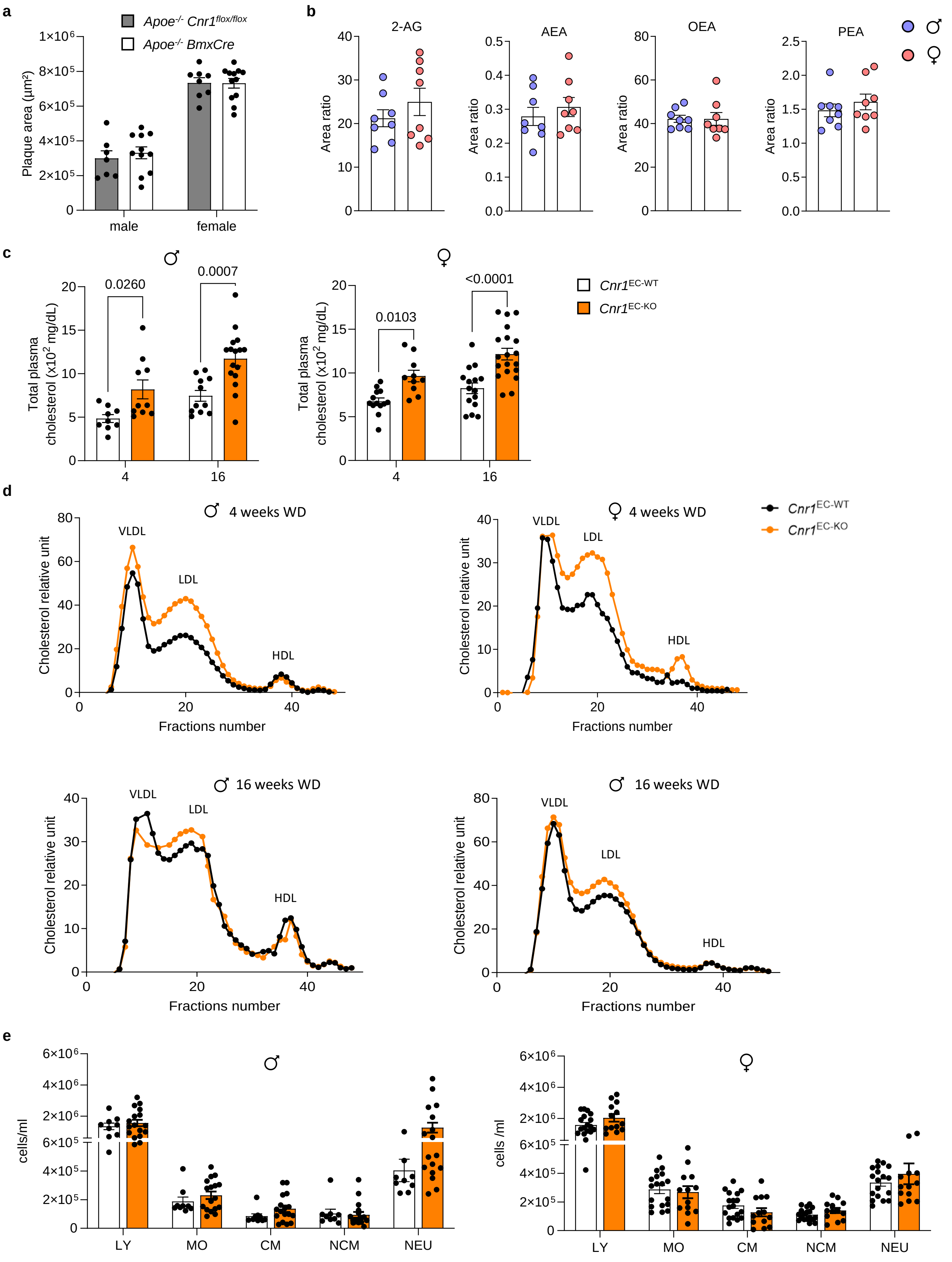

Supplementary Fig. 6. Plasma parameters and blood leukocytes in male and female *Cnr1*$^{EC\text{-}KO}$ mice. a Quantification of absolute lesion area in the aortic root of male and female *Apoe*$^{-/-}$*Bmx*^{Cre} and *Apoe*$^{-/-}$*Cnr1*$^{flox/flox}$ mice after 16 weeks of WD (n=7-14). b Plasma endocannabinoid levels, 2-arachidonoylglycerol (2-AG), anandamide (AEA), oleoylethanolamide (OEA), and palmitoylethanolamide (PEA) in male and female Apoe$^{-/-}$ mice after 4 weeks of WD. **c** Plasma total cholesterol and **d** lipoprotein profiles in male and female *Cnr1*$^{EC\text{-}WT}$ and *Cnr1*$^{EC\text{-}KO}$ mice after 4 or 16 weeks WD **e** Flow cytometry analysis of lymphocytes (LY), monocytes (MO), classical (CM), non-classical monocytes (NCM), and neutrophils (NEU) in peripheral blood of *Cnr1*$^{EC\text{-}WT}$ and *Cnr1*$^{EC\text{-}KO}$ male and female mice after 4 weeks WD. Data are shown as mean ± s.e.m.; Two-way ANOVA with Sidak *post hoc* (**a**, **c**), Mann-Whitney *U* (**b**), Multiple Mann-Whitney *U* (**e**) tests were applied. Each data point represents one individual human or mouse sample (biological replicate), collected in at least 2 independent experiments.

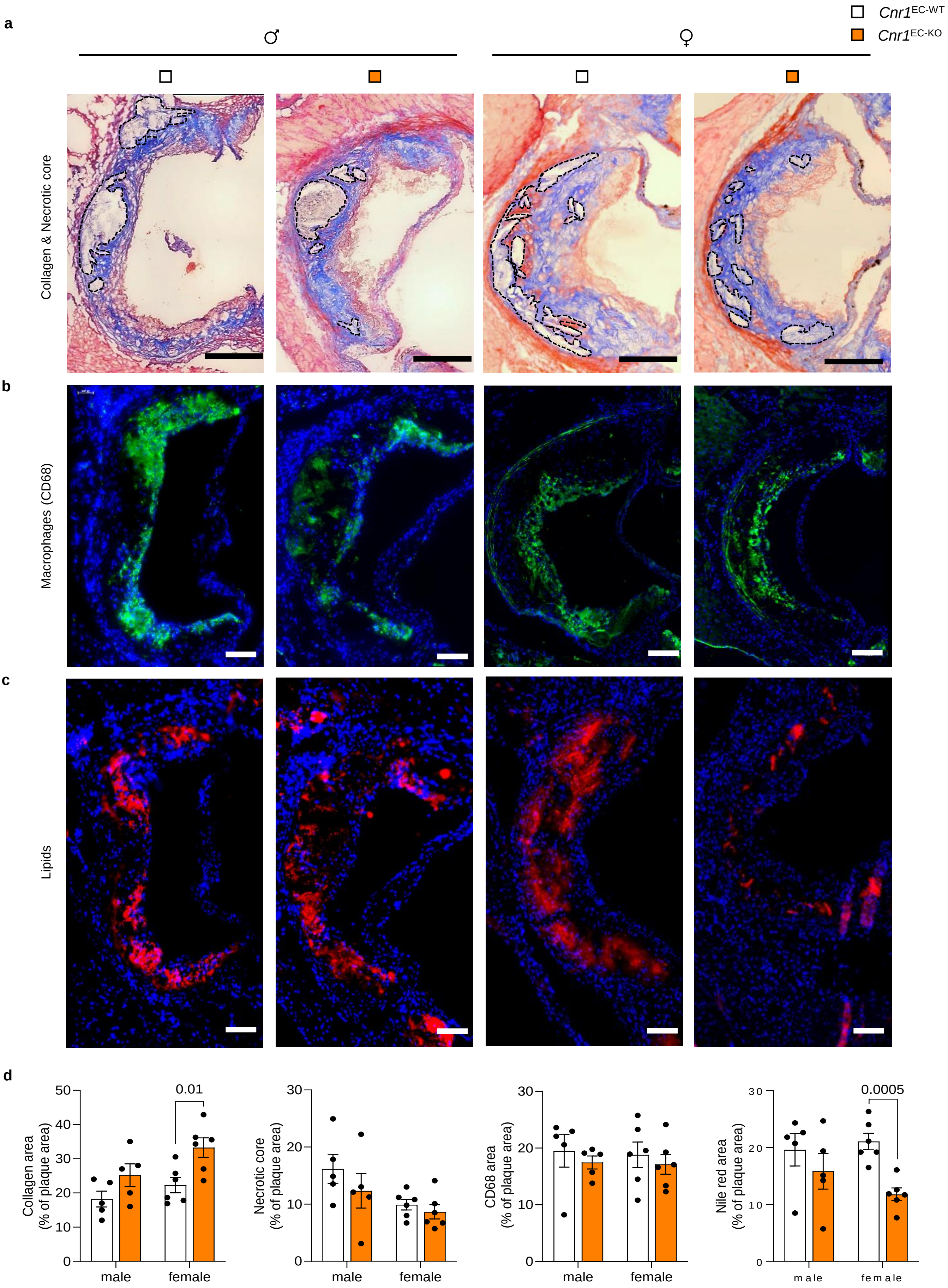

Supplementary Fig. 7. Effect of *Cnr1* deficiency on advanced plaque composition. Representative aortic root plaque images from $Cnr1^{EC\text{-}WT}$ and $Cnr1^{EC\text{-}KO}$ mice after 16 weeks WD (n=5-6 biological replicates) stained with **a** Masson's trichrome staining for collagen and necrotic core area (encircled by dotted lines). Scale bar, 200 µm. **b** CD68 immunostaining for macrophages and **c** Nile red staining for intracellular lipids. Scale bar, 100 µm. **d** Quantification of relative areas per plaque area. Data are shown as mean ± s.e.m.; unpaired Student's *t*-test was applied separately for males and females. Each data point represents one individual mouse sample (biological replicate), collected in at least 2 independent experiments.

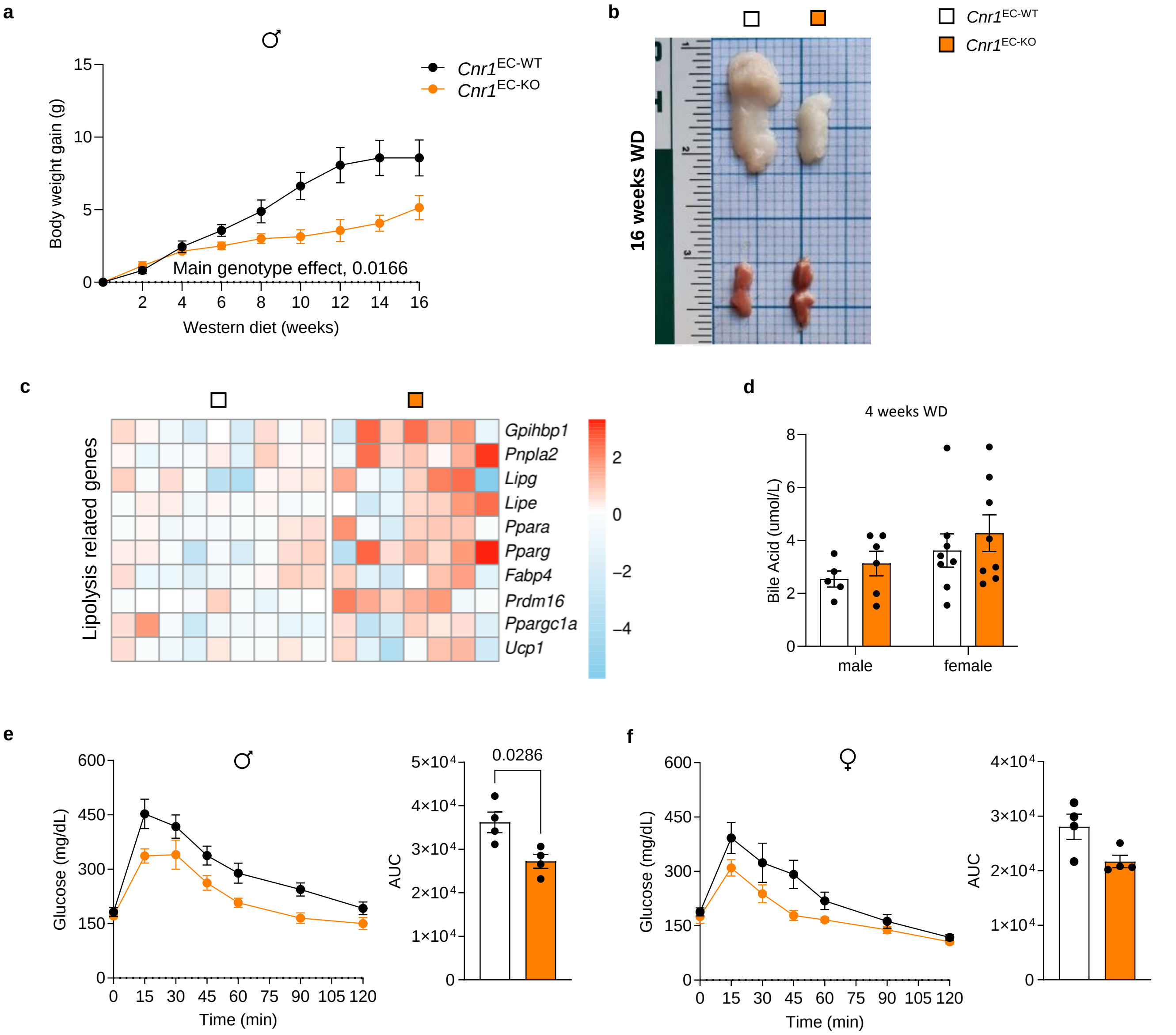

Supplementary Fig. 8. Impact of endothelial *Cnr1* deficiency on metabolic parameters. a Body weight gain over 16 weeks WD in male $Cnr1^{EC\text{-}WT}$ and $Cnr1^{EC\text{-}KO}$ mice (n=7-8). **b** Representative images of epididymal white (eWAT) and brown adipose tissue (BAT) from male mice after 16 weeks of WD. **c** Gene expression analysis (RT-qPCR) in BAT of male $Cnr1^{EC\text{-}WT}$ and $Cnr1^{EC\text{-}KO}$ mice after 4 weeks of WD (n = 9). Log2 transformed values visualized in heatmap plotted by using pheatmap in R. T test with FDR (Benjamini Hochberg) performed. **d** Plasma bile acid levels in male (n=5-6) and female (n=8) $Cnr1^{EC\text{-}WT}$ and $Cnr1^{EC\text{-}KO}$ mice after 4 weeks of WD. **e-f** Levels of plasma glucose during intraperitoneal glucose tolerance test and AUC in male (**e**) and female (**f**) $Cnr1^{EC\text{-}WT}$ and $Cnr1^{EC\text{-}KO}$ after 4 weeks of WD (n=4). Data are shown as mean ± s.e.m.; mixed-effect model with analysis of the main fixed effect- genotype (**a**), *t* test with FDR-Benjamini-Hochberg (**c**), unpaired Student's *t* test (**d**), or Mann Whitney *U* test to compare AUCs (**e**, **f**) were applied. Each data point represents one individual mouse sample (biological replicate), collected in at least 2 independent experiments.

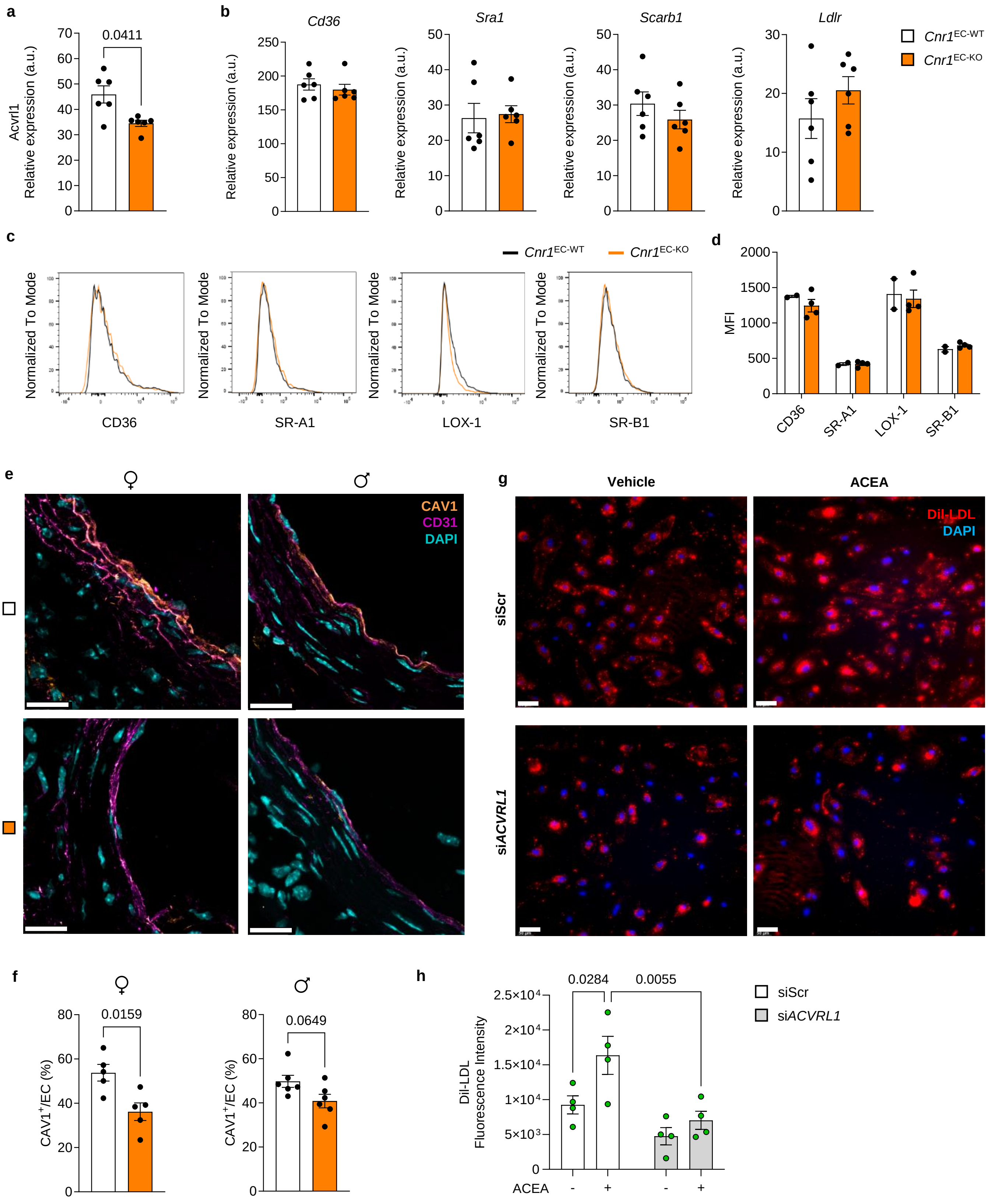

Supplementary Fig. 9. Impact of endothelial *Cnr1* deficiency on endothelial lipid receptors. a Endothelial ALK1 encoding gene expression retrieved from the RNA sequencing data of sorted female aortic *Cnr1*$^{EC\text{-}WT}$ and *Cnr1*$^{EC\text{-}KO}$ ECs (n=6). **b** Expression of lipid receptors retrieved from RNA sequencing data of sorted aortic ECs from female *Cnr1*$^{EC\text{-}WT}$ and *Cnr1*$^{EC\text{-}KO}$ mice (n=6). **c**, **d** Flow cytometric analysis of lipid receptor surface expression on aortic endothelial cells of female *Cnr1*$^{EC\text{-}WT}$ (n=2) and *Cnr1*$^{EC\text{-}KO}$ (n=4) mice after 4 weeks WD; gated as live CD45^{-}CD31^{+}CD107a^{+}. **e** Representative images of caveolin-1 (CAV1, orange) and CD31 (magenta) staining of consecutive aortic root sections of *Cnr1*$^{EC\text{-}WT}$ and *Cnr1*$^{EC\text{-}KO}$ male and female mice after 4 weeks WD (scale bar, 20 µm). **f** Quantification of endothelial CAV1 staining normalized to EC percentage from e (n=5). **g** Representative images of female HoAECs (n=4) pre- treated with siScr (Scramble) or si*ACVRL1* exposed to OSS with Vehicle or ACEA stained with Dil-LDL (red) and nuclei (blue) (scale bar, 20 µm). **h** Quantification of Dil-LDL from g (n=4). Data are shown as mean ± s.e.m. and Mann Whitney *U* test **(a, b, d, f)** or two-way ANOVA with Sidak *post hoc* was applied (**h**). Each data point represents one individual mouse or human sample (biological replicate), collected in at least 2 independent experiments.

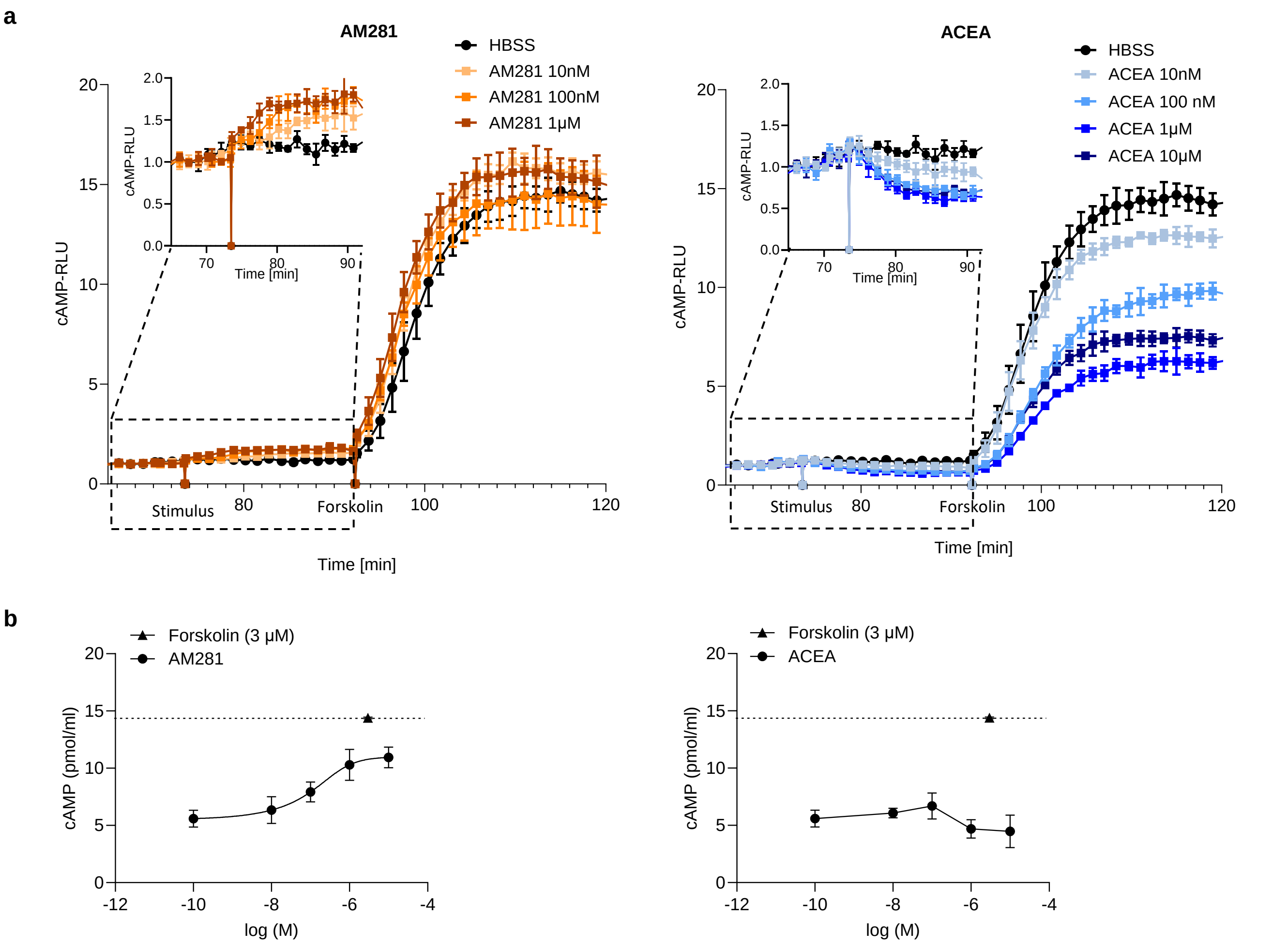

Supplementary Fig. 10. Impact of endothelial *Cnr1* deficiency on CB1-dependent regulation of cAMP. a Representative Glosensor cAMP reporter assay in *CNR1* and cAMP-luciferase expressing HEK293 cells, treated with buffer (HBSS) alone or CB1 antagonist AM281 or agonist ACEA, as well as the adenylate cyclase activator forskolin. **b** cAMP levels (ELISA) in HAoECs of female donors (n=2) 20 min after treatment with AM281, ACEA, or forskolin. Data are shown as mean ± s.e.m. Each data point represents one individual human sample (biological replicate), collected in at least 2 independent experiments.

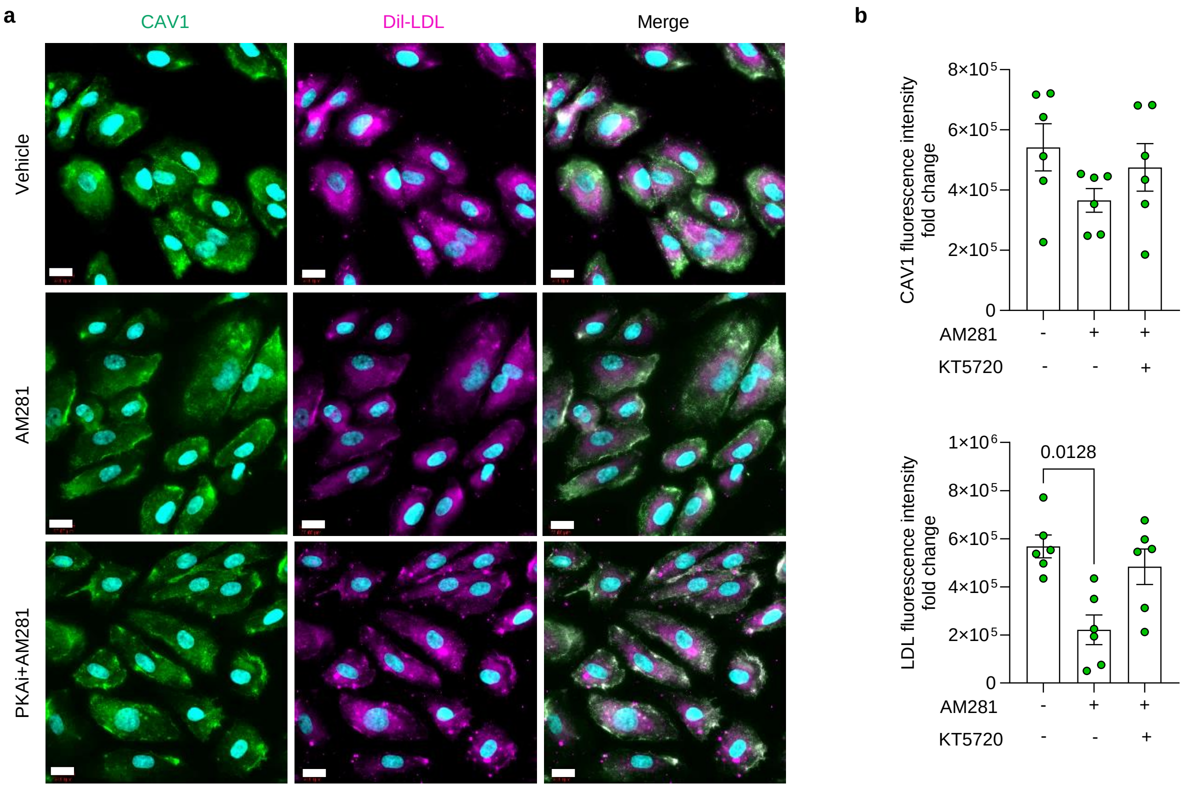

Supplementary Fig. 11. DiI-LDL uptake in HAoECs of male donors. a Representative immunofluorescence analysis of CAV1 expression and DiI-LDL uptake in HAoECs of male donors (n= 6) treated with 1 µM AM281 alone or in the presence of 1 µM PKA inhibitor (KT5720) or vehicle (DMSO) under OSS for 24 h. Scale bar, 20 µm. **b** Quantification of images shown in **a**. Data are shown as mean ± s.e.m.; Kruskal-Wallis *H* with Dunn´s *post hoc* test was applied. Each data point represents one individual human sample (biological replicate), collected in at least 2 independent experiments.

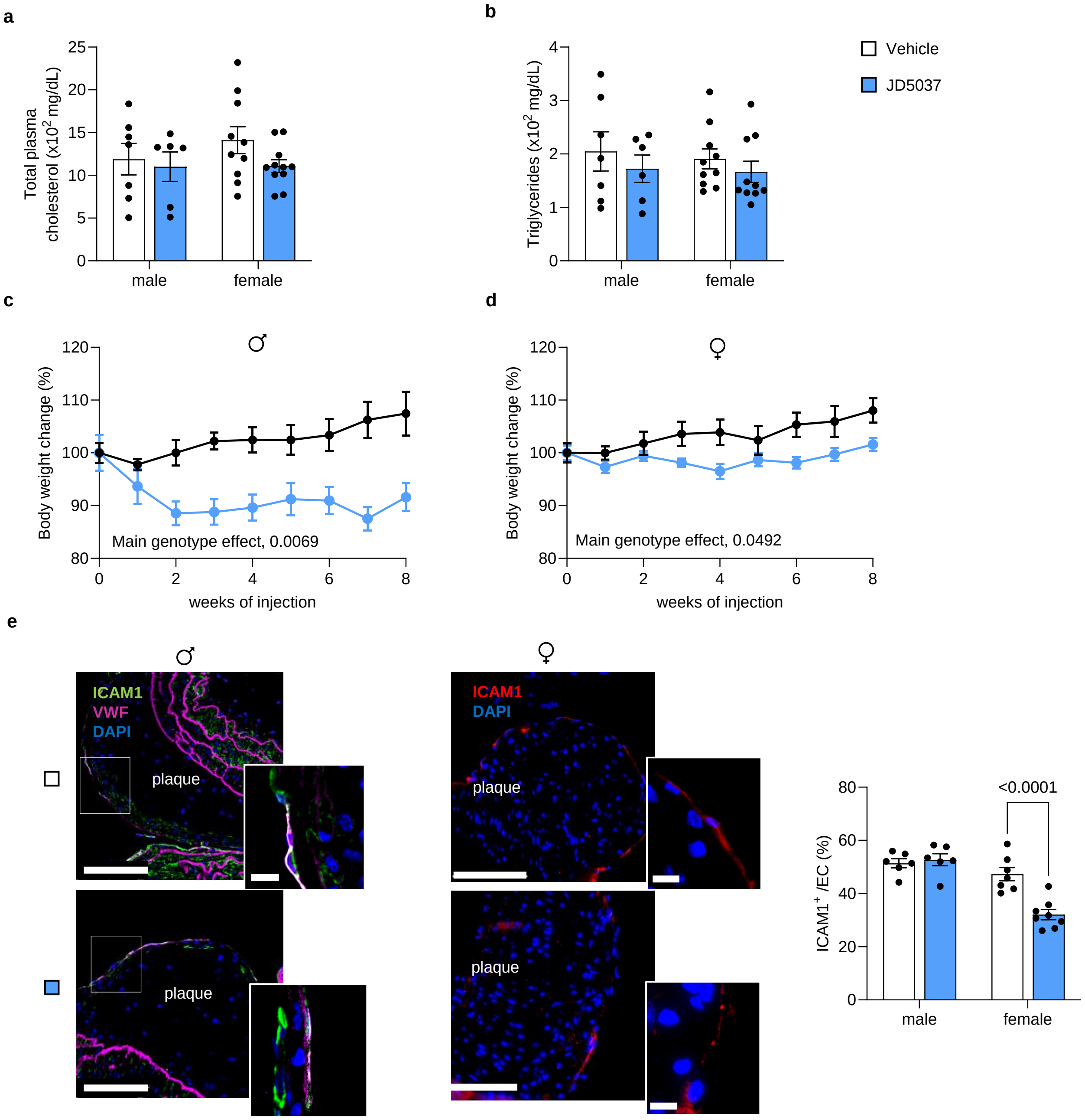

Supplementary Fig. 12. Impact of peripheral CB1 antagonist JD5037 on metabolic parameters and plaque endothelial ICAM1 expression.
a Plasma cholesterol and **b** triglyceride levels in male and female *Ldlr*$^{-/-}$ mice (n=6-8) after 16 weeks WD and daily JD or vehicle injections for the last 8 weeks. Body weights changes in male **c** and female **d** *Ldlr*$^{-/-}$ mice (n=6-8) during the 8 weeks of JD5037 or vehicle treatment was measured. **e** Representative images and quantification of ICAM1 (red) positive endothelial cells in aortic arch lesions of male and female *Ldlr*$^{-/-}$ mice (n=6-8) treated with vehicle or JD5037. Nuclei were stained with DAPI (blue). Scale bar, 100 μm (overview) and 10 μm (insert). Each data point represents a mouse, and all data are expressed as mean ± s.e.m.; mixed-effect model with analysis of the main fixed effect- genotype (**c**) or two-way ANOVA with Sidak correction (**e**) was applied. Each data point represents one individual mouse sample (biological replicate), collected in at least 2 independent experiments.

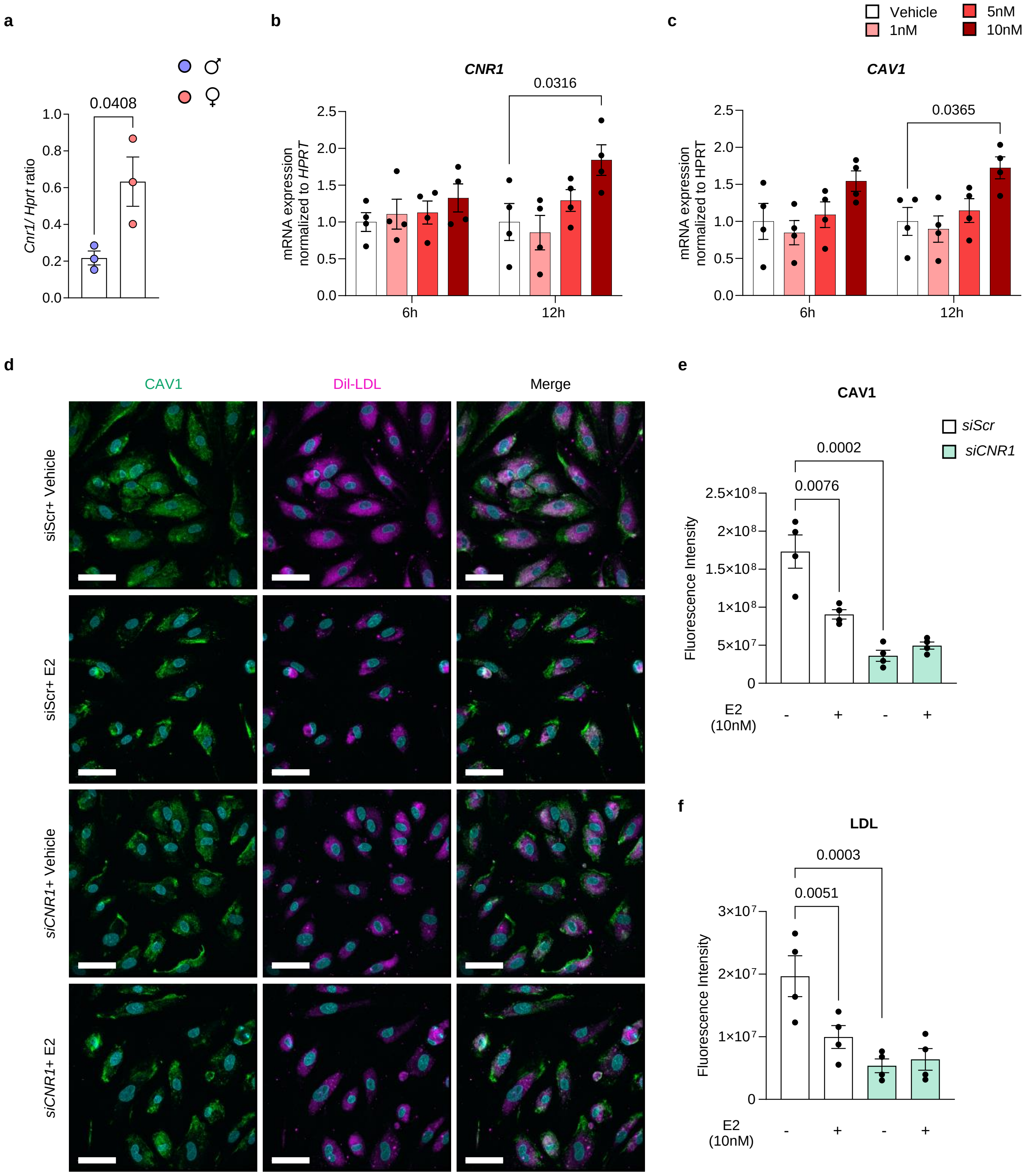

Supplementary Fig. 13. Effect of oestrogen signalling on *CNR1* and *CAV1* in endothelial cells. **a** Levels of *Cnr1* in comparison to *Hprt* from sorted female murine endothelial cells using ddPCR. **b, c** *CNR1* and *CAV1* mRNA expression (RT-qPCR) in HAoECs of female donors (n= 4 biological replicates) treated with estrogen agonist estradiol (E2; 1nM, 5nM, 10nM) or vehicle for 6h and 12h. **d** Analysis of CAV1 expression and Dil-LDL uptake in HAoECs from female donors was conducted after transfection with 20 nM scrambled siRNA (siScr) or CNR1 (siCNR1) and a 24-hour incubation period, followed by treatment with 10 nM estrogen agonist (E2) or vehicle (ethanol) for 12 h. Scale bar, 50 µm. Quantification of CAV1 expression (**e**) and Dil-LDL uptake (**f**) shown in d (n= 4 biological replicates). Data are shown as mean ± s.e.m.; unpaired Student's *t* test (**a**) or two-way ANOVA with Tukey correction (**b**, **c, e, f**) was applied. Each data point represents one individual mouse sample (biological replicate), collected in at least 2 independent experiments.

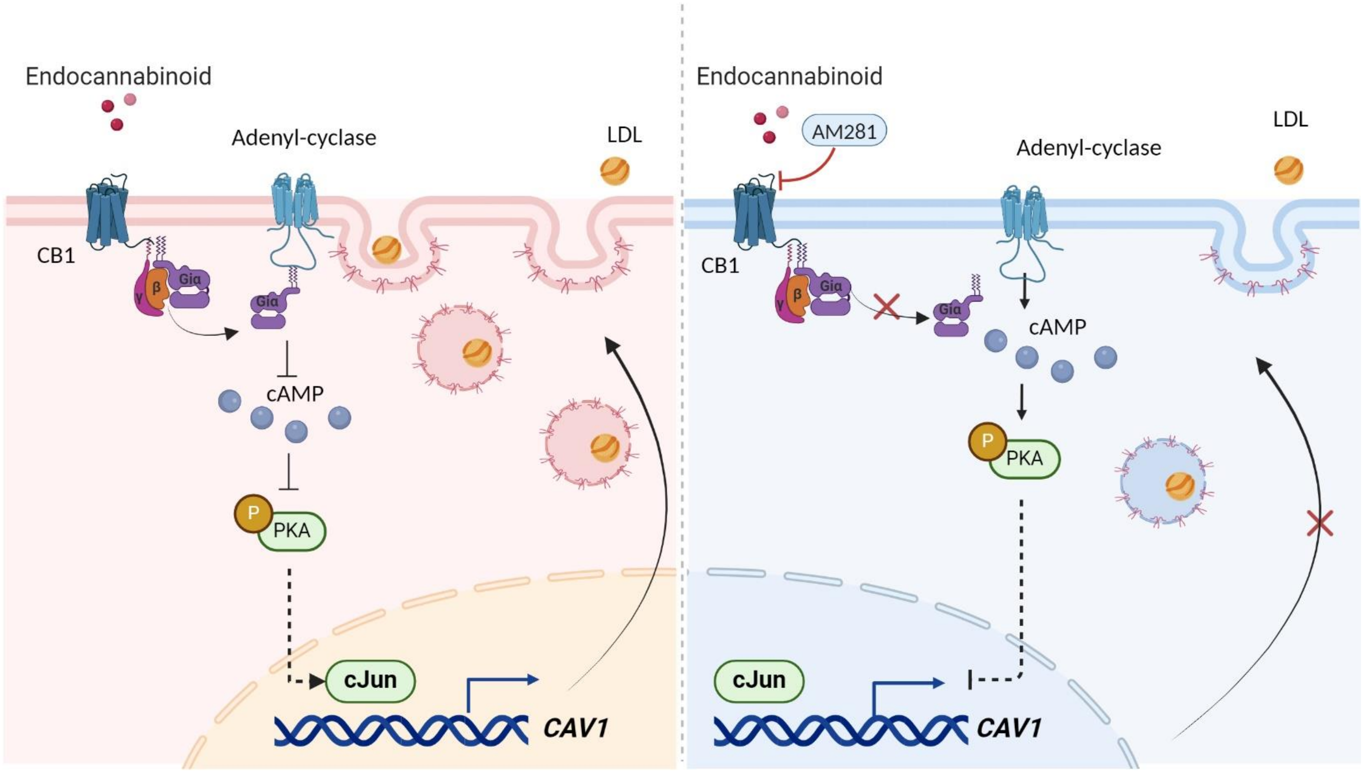

Supplementary Fig. 14. Proposed mechanism how CB1 regulates endothelial CAV1 expression and LDL uptake- Created in BioRender: Steffens, S. (2026) https://BioRender.com/dhemg9j**.** (**Left**) Endocannabinoid-CB1 signaling leads to a G_i-dependent inhibition of adenylate cyclase and consequently to an inhibition of intracellular cAMP production. Reduced cAMP-dependent PKA activity prevents PKA-dependent inhibition of *CAV1* gene expression and LDL uptake. (**Right**) Antagonism of CB1 prevents the depletion of intracellular cAMP and thereby enables PKA activation, which inhibits *CAV1* gene expression and caveolae-dependent LDL uptake.

Supplementary Table 1: Antibodies used for immunostaining

Antigen	Reference	Host	Dilution	Provider
Caveolin-1	PA5-17447	Rabbit	1:100	Invitrogen
CD31 (PECAM-1)	553370	Rat	1:50	BD
CD54 (ICAM1)	553250	Hamster	1:100	BD
CD68	MCA1957GA	Rat	1:400	Bio-Rad
CD106 (VCAM1)	553329	Rat	1:100	BD
CD144 (VE-Cadherin)	555289	Rat	1:100	BD
GPIHBP1	PA5-98598	Rabbit	1:100	Invitrogen
Hoechst	H1399		1:1000	Invitrogen
LPL	AF7197-SP	Goat	1:100	R&D system
Phospho-c-Jun	3270T	Rabbit	1:100	Cell Signaling
vWF (Von Willebrand Factor)	Ab11713	Sheep	1:300	Abcam

Blocking buffer: 6 ml PBS, 600 µl 10 % BSA (1 %), 3 drops horse serum (S-2000, Vector Laboratories). Antigen Retrieval buffer: 630 ml Aqua dest, 12,6 ml Solution A (21,01 g Citric Acid, 1 L Aqua dest), 57.4 ml Solution B (29,4 g Sodium Citrate, 1 L Aqua dest), 350 µl Tween20.

Supplementary Table 2: Secondary antibodies

Antigen	Source	Conjugation	Dilution	Reference	Provider
Anti-goat	Donkey	AlexaFluor594	1:100	705-585-003	JIR
Anti-hamster	Goat	Cy3	1:300	127-165-160	JIR
Anti-rabbit IgG	Donkey	AlexaFluor647	1:600	711-605-152	JIR
Anti-rabbit IgG	Donkey	Cy3	1:300	711-165-152	JIR
Anti-rat	Donkey	AlexaFluor488	1:300	A21208	Invitrogen
Anti-rat	Donkey	Cy3	1:300	712-165-153	JIR
Anti-sheep	Donkey	DyLight® 488	1:300	ab96939	Abcam
Anti-sheep IgG	Donkey	Cy5	1:600	713-175-147	JIR
Anti-sheep IgG	Donkey	Cy3	1:600	713-165-003	JIR

Supplementary Table 3: Isotype controls

Immunoglobulin	Reference	Provider
Armenian hamster IgG	553969	BD
Normal Goat IgG	ab-108-c	R&D system
Normal rabbit IgG	315-005-003	JIR
Normal rat IgG	6-001-A	R&D system
Normal Sheep IgG	515-005-003	JIR

The working concentration of normal IgG isotype control was equal to the corresponding primary antibody. IgG = Immunoglobulin G; JIR = Jackson ImmunoResearch.

Supplementary Table 4: Antibodies used for TPLSM

Antigen	Reference	Host	Dilution	Provider
CD31/eFluor450 (PECAM-1)	48-0311-82	Rat	1:100	eBioscience^{TM}
Dil-LDL	770230-9	human	1:10	KalenBiomedica

Supplementary Table 5: Murine flow cytometry antibodies

Antigen	Conjugation	Dilution	Reference	Provider
CD11b	PerCP	1:500	101230	Biolegend
CD16/32	purified	1:1000	553142	BD
CD31	PE-Cy7	1:100	102417	Biolegend
CD36	APC	1:1000	Ab133625	Abcam
CD45	Alex eFluoro 780	1:400	47-0451-82	Invitrogen
CD45.2	FITC	1:500	553772	BD
CD54 (ICAM-1)	APC	1:500	116119	Biolegend
CD106 (VCAM-1)	PerCP	1:500	105715	Biolegend
CD107a	BV421	1:400	121617	Biolegend
CD115	APC	1:500	17-115-282	eBioscience
Live/dead	Zombie Green	1:800	77476	Biolegend
LOX1	AF647	1:100	FAB1564R	R&D system
Ly6C	PE- Cy7	1:500	560593	BD
Ly6G	APC-Cy7	1:500	127623	Biolegend
SRA1	FITC	1:500	ab151707	Abcam
SRB1	FITC	1:500	NB400-104F	NOVUS

APC = Allophycocyanin; BV = Brilliant violet; Cy = Cyanine; FC = Flow Cytometry; FITC = Fluorescein isothiocyanate; IF=Immunofluorescence; PB = Pacific Blue; PE = Phycoerythrin; PerCP = Peridinin chlorophyll.

Supplementary Table 6: Enzymes for aorta digestion

Enzyme	Final concentration	Company
Collagenase IV	10 mg/ml	Worthington Biochemical Corp, Lakewood,
DNAse I	20 U/ml	Roche, Basel, Switzerland

Supplementary Table 7: Enzymes for BAT digestion

Enzyme	Final concentration	Company
Collagenase I	450 U/ml	Worthington Biochemical Corp, Lakewood,
CollagenaseXI	125 U/ml	Worthington Biochemical Corp, Lakewood,
DNAse I	60 U/ml	Roche, Basel, Switzerland
Hyaluronuclease	60 U/ml	Sigma-Aldrich Chemie GmbH, Munich,

Supplementary Table 8: Primers for qPCR analysis (Human)

Gene	Assay ID/5’ to 3’ primer sequence
HPRT	Fw: 5’- TGG TCA GGC AGT ATA ATC CAA AGA-3’
	Rev: 5’- TCA AAT CCA ACA AAG TCT GGC TTA-3’
	Probe: 5’-AGC TTG CGA CCT TGAC-3’ TAMRA
CNR1	Fw:5’- CTG GCA TCT ATC TGG TGA TTT-3’
	Rev: 5’- CTT AGA GCG TGA ACC GTA AG-3’
	Probe: 5’- CGA GAT ACC CAA GCA GCC TGA TGG -3’ TAMRA
ICAM1	Hs00164932_m1
VCAM1	Hs01003372_m1
KLF2	Hs00360439_g1
IL6	Hs00174131_m1
CXCL8	Hs00174103_m1
CCL2	Hs00234140_m1
SELE	Hs00174057_m1
CAV1	Hs00971716_m1
ACVRL1	Hs00953798_m1
NOS3	Hs01574665_m1
PFKFB3	Hs00998698_m1
CD14	Hs02621496_s1

Self-designed primers and probes for qPCR were purchased from MWG-Biotech AG and TaqMan Gene Expression Arrays were obtained from Life Technologies. ddPCR = droplet digital PCR; Fw = Forward; Rev = Reverse.

Supplementary Table 9: Primers for qPCR analysis (Murine)

Gene	Assay ID/5' to 3' primer sequence
Hprt	Fw: 5'- GACCGGTCCCGTCATGC-3'
	Rev: 5'- TCATAACCTGGTTCATCATCGC-3'
	Probe: VIC-ACCCGCAGTCCCAGCGTCGTG-TAMRA
Hprt	Fw: GACCGGTCCCGTCATGC
(ddPCR)	Rev: TCATAACCTGGTTCATCATCGC
	Probe: 5HEX-ACCCGCAGT/ZEN/CCCAGCGTCGTG-3IABkFQ
Cnr1	Fw: 5'-ATGCGAAGGGGTTCCCTC-3'
	Rev: ATGGTACGGAAGGTGGTATCT
	Probe: FAM-TGGCACCTCTTTCTCAGTCACGTTGAGC-TAMRA
Cnr1	Fw: 5'-ATGCGAAGGGGTTCCCTC-3'
(ddPCR)	Rev: ATGGTACGGAAGGTGGTATCT
	Probe: FAM-TGGCACCTCTTTCTCAGTCACGTTGAGC-TAMRA
Gpihbp1	Mm01205849_g1
Pnpla2	Mm00503040_m1
Lipg	Mm00495368_m1
Lipe	Mm00495359_m1
Ppara	Mm00440939_m1
Pparg	Mm00440940_m1
Fabp4	Mm00445878_m1
Prdm16	Mm00712556_m1
Ppargc1a	Mm00447181_m1
Ucp1	Mm01244861_m1
Acadm	Mm01323360_g1
Cd36	Mm01135198_m1
Cpt1a	Mm01231183_m1
Cpt2	*Mm00487205_m1*

Self-designed primers and probes for qPCR were purchased from MWG-Biotech AG and TaqMan Gene Expression Arrays were obtained from Life Technologies. ddPCR = droplet digital PCR; Fw = Forward; Rev = Reverse.

Supplementary Table 10: siRNA SMARTpool Target Sequence

Organism	Gene	siRNA SMARTpool Target Sequence
Human	Scramble	UGGUUUACAUGUCGACUAA
		UGGUUUACAUGUUGUGUGA
		UGGUUUACAUGUUUUCUGA
		UGGUUUACAUGUUUUCCUA
	CNR1	GCGAGAAACUGCAAUCUGU
		GACCAUAGCCAUUGUGAUC
		GGACAUAGAGUGUUUCAUG
		CAAGAGCACGGUCAAGAUU
	CAV1	CUAAACACCUCAACGAUGA
		GCAAAUACGUAGACUCGGA
		GCAGUUGUACCAUGCAUUA
		GCAUCAACUUGCAGAAAGA
