# Supplementary material for: Endothelial cannabinoid CB1 receptor deficiency reduces shear stress-induced arterial inflammation and lipid uptake
Source: Nat Commun. 2026 Jul 7;17:5939. doi: 10.1038/s41467-026-75214-2 (PMC13342577; doi:10.1038/s41467-026-75214-2)
Supplement: Supplementary file 2 — Reporting summary [file 41467_2026_75214_MOESM2_ESM.pdf]

Corresponding author(s): Sabine Steffens
Last updated by author(s): Jun 7, 2026

Reporting Summary

Nature Portfolio wishes to improve the reproducibility of the work that we publish. This form provides structure for consistency and transparency in reporting. For further information on Nature Portfolio policies, see our Editorial Policies and the Editorial Policy Checklist.

Statistics

For all statistical analyses, confirm that the following items are present in the figure legend, table legend, main text, or Methods section.

n/a	Confirmed	
☐	☒	The exact sample size (*n*) for each experimental group/condition, given as a discrete number and unit of measurement
☐	☒	A statement on whether measurements were taken from distinct samples or whether the same sample was measured repeatedly
☐	☒	The statistical test(s) used AND whether they are one- or two-sided<br>*Only common tests should be described solely by name; describe more complex techniques in the Methods section.*
☐	☒	A description of all covariates tested
☐	☒	A description of any assumptions or corrections, such as tests of normality and adjustment for multiple comparisons
☐	☒	A full description of the statistical parameters including central tendency (e.g. means) or other basic estimates (e.g. regression coefficient) AND variation (e.g. standard deviation) or associated estimates of uncertainty (e.g. confidence intervals)
☐	☒	For null hypothesis testing, the test statistic (e.g. *F*, *t*, *r*) with confidence intervals, effect sizes, degrees of freedom and *P* value noted<br>*Give P values as exact values whenever suitable.*
☒	☐	For Bayesian analysis, information on the choice of priors and Markov chain Monte Carlo settings
☒	☐	For hierarchical and complex designs, identification of the appropriate level for tests and full reporting of outcomes
☐	☒	Estimates of effect sizes (e.g. Cohen's *d*, Pearson's *r*), indicating how they were calculated

Our web collection on statistics for biologists contains articles on many of the points above.

Software and code

Policy information about availability of computer code

Data collection	Any specific software was used for data collection.
Data analysis	R (4.2.0), SeuratWrappers (v0.3.5, R v4.4.1), Flow Jo (v10.2), Graph Pad (10.5), Image J (1.54), and Leica LASX (V4.3) software were used for data analysis. BioRender was used for experimental schemes and a graphical summary.

For manuscripts utilizing custom algorithms or software that are central to the research but not yet described in published literature, software must be made available to editors and reviewers. We strongly encourage code deposition in a community repository (e.g. GitHub). See the Nature Portfolio guidelines for submitting code & software for further information.

Data

Policy information about availability of data

All manuscripts must include a data availability statement. This statement should provide the following information, where applicable:

- Accession codes, unique identifiers, or web links for publicly available datasets
- A description of any restrictions on data availability
- For clinical datasets or third party data, please ensure that the statement adheres to our policy

The RNA sequencing data generated in this study have been deposited in the GEO database under accession code GSE260826. All other data generated in this study are provided in the Source Data file.

Research involving human participants, their data, or biological material

Policy information about studies with human participants or human data. See also policy information about sex, gender (identity/presentation), and sexual orientation and race, ethnicity and racism.

Reporting on sex and gender	From the GSE data that we have analysed, on average a 84.62% of the cases were males. The demographic and clinical characteristics of the patients used in the scRNAseq analysis is summarized in detail in the Supplementary Table 1 of the following paper (reference 20 in our manuscript): Bashore AC, Yan H, Xue C, Zhu LY, Kim E, Mawson T, Coronel J, Chung A, Sachs N, Ho S, Ross LS, Kissner M, Passegué E, Bauer RC, Maegdefessel L, Li M, Reilly MP. High-Dimensional Single-Cell Multimodal Landscape of Human Carotid Atherosclerosis. Arterioscler Thromb Vasc Biol. 2024 Apr;44(4):930-945. doi: 10.1161/ATVBAHA.123.320524. Epub 2024 Feb 22. PMID: 38385291; PMCID: PMC10978277.
Reporting on race, ethnicity, or other socially relevant groupings	Reported ethnicity: 15% of hispanic, 77% of bon-hispanic and 8% non reported.<br>Reported race: 61.5% whit, 7.7% african amercian, 0% asian, 23,1% more than one race or not declared and 7,7% was not reported.<br>All demographic and clinical characteristics of the patients used in the scRNAseq analysis is summarized in detail in the Supplementary Table 1 of the following paper (reference 20 in our manuscript): Bashore AC, Yan H, Xue C, Zhu LY, Kim E, Mawson T, Coronel J, Chung A, Sachs N, Ho S, Ross LS, Kissner M, Passegué E, Bauer RC, Maegdefessel L, Li M, Reilly MP. High-Dimensional Single-Cell Multimodal Landscape of Human Carotid Atherosclerosis. Arterioscler Thromb Vasc Biol. 2024 Apr;44(4):930-945. doi: 10.1161/ATVBAHA.123.320524. Epub 2024 Feb 22. PMID: 38385291; PMCID: PMC10978277.
Population characteristics	Single-cell expression data were retrieved from the datasets generated as part of Bashore et al. available via Gene Expression Omnibus (GEO, accession code: GSE253904
Recruitment	We used data from a public database. The following is reported in ref. 20: Human carotid atherosclerotic plaques were collected from twenty-one patients undergoing carotid endarterectomy surgery. Exclusion criteria include current infection, known immune system disorder, and active or recent (within last three months) radiation, chemotherapy, hormone-based, and/or immunotherapy treatment for cancer.
Ethics oversight	We used data from a public database. Ihe following is reported in ref. 20: These human subject studies were performed with approval (protocol number AAAJ2765) of the local Institutional Review Board (IRB) of Columbia University Irving Medical Center, and written informed consent was obtained from all participants.

Note that full information on the approval of the study protocol must also be provided in the manuscript.

Field-specific reporting

Please select the one below that is the best fit for your research. If you are not sure, read the appropriate sections before making your selection.

☒ Life sciences ☐ Behavioural & social sciences ☐ Ecological, evolutionary & environmental sciences

For a reference copy of the document with all sections, see nature.com/documents/nr-reporting-summary-flat.pdf

Life sciences study design

All studies must disclose on these points even when the disclosure is negative.

Sample size	Sample size for the experiments was selected to achieve an a priori 85% statistical power for biologically significant difference (d=0.8).
Data exclusions	To test for Gaussian distribution, D'Agostino Pearson omnibus or Shapiro–Wilk normality test was applied. Outliers were determined by Grubbs' test (alpha 0.05).
Replication	Experiments were replicated at least two times, except for RNA-seq analysis to avoid batch effects (Fig. 3a & 5g), transmission electron microscopy (Fig. 6c-d), the pilot time course experiment for phospho-c-Jun (Suppl. Fig4b), cAMP assays (Suppl. Fig. 10), ddPCR of sorted mouse aortic ECs (Suppl. Fig. 13a)
Randomization	When necessary, a number was assigned to the mice and they were randomized in the experimental groups using http://www.randomizer.org.
Blinding	Investigators were blinded during sample acquisition and data analysis.

Reporting for specific materials, systems and methods

We require information from authors about some types of materials, experimental systems and methods used in many studies. Here, indicate whether each material, system or method listed is relevant to your study. If you are not sure if a list item applies to your research, read the appropriate section before selecting a response.

Materials & experimental systems

n/a	Involved in the study
☐	☒ Antibodies
☐	☒ Eukaryotic cell lines
☒	☐ Palaeontology and archaeology
☐	☒ Animals and other organisms
☒	☐ Clinical data
☒	☐ Dual use research of concern
☒	☐ Plants

Methods

n/a	Involved in the study
☒	☐ ChIP-seq
☐	☒ Flow cytometry
☒	☐ MRI-based neuroimaging

Antibodies

Antibodies used	We provide a supplementary table with all the antibodies that we used, specifying the supplier and reference to indicate the antibody clone.
Validation	We followed manufacturer's recommendation; for flow cytometry analyses the best antibody concentration was determined by calculating the stain index.

Eukaryotic cell lines

Policy information about cell lines and Sex and Gender in Research

Cell line source(s)	Human Primary Aortic Endothelial Cells (HAoECs) from a 61-year-old female donor (458Z035.1; C-12271; PromoCell) or a 50-year-old male donor (434Z005.1; C-12271; PromoCell).
Authentication	PromoCell autentified the cell lines, they were bought and directly used for the reported experiments in this manuscript.
Mycoplasma contamination	All cell lines were tested negative for mycoplamsa analysis.
Commonly misidentified lines (See ICLAC register)	No misidentified cell lines were used; only well identified commercial cell lines were used.

Animals and other research organisms

Policy information about studies involving animals; ARRIVE guidelines recommended for reporting animal research, and Sex and Gender in Research

Laboratory animals	ApoE−/−; strain # 002052 (The Jackson Laboratory) and Cnr1flox/flox mice were kindly provided by Beat Lutz. We generated Apoe-/-Cnr1flox/flox mice that were then crossed with BmxCreERT2 mice (Ralf Adams, PMID: 23785053) to obtain Apoe-/-BmxCre(+/-)Cnr1flox/flox mice (referred to as Cnr1EC-KO). Ldlr-/- mice (strain #002207) was obrained from The Jackson Laboratory.
Wild animals	The study did not involved wild animals.
Reporting on sex	We performed all our experiments in male and female mice/cell lines. In each figure is specified the sex of the data presented in the manuscript.
Field-collected samples	Animals were housed in ventilated cages, with 4 to 6 mice per cage. The environment was air-conditioned, with a 12-hour light-dark cycle and a temperature of 23°C and 60% relative humidity
Ethics oversight	All animal procedures were approved by the local Ethics committee (District Government of Upper Bavaria; license number: 55.2-1-54-2532-111-13 and 55.2-2532.Vet_02-18-114) and conducted in accordance with the institutional and national guidelines and following the ARRIVE guidelines.

Note that full information on the approval of the study protocol must also be provided in the manuscript.

Plants

Seed stocks	No plants were used in our study.
Novel plant genotypes	No plants were used in our study.
Authentication	No plants were used in our study.

Flow Cytometry

Plots

Confirm that:

☒ The axis labels state the marker and fluorochrome used (e.g. CD4-FITC).

☒ The axis scales are clearly visible. Include numbers along axes only for bottom left plot of group (a 'group' is an analysis of identical markers).

☒ All plots are contour plots with outliers or pseudocolor plots.

☒ A numerical value for number of cells or percentage (with statistics) is provided.

Methodology

Sample preparation	Freshly collected whole blood (50 µl) was transferred to ice-cold FACS tubes and subjected to red blood cell lysis with incubated with an antibody mix, washed and acquired by the flow cytometers.<br>Murine aortas spanning from the aortic arch to the iliac bifurcation were isolated after perfusion with PBS and digested with collagenase IV and DNase I at 37°C for 40 min. The interscapular brown adipose tissue (BAT) was collected, cut into small pieces and digested with collagenase I, collagenase XI, DNase I, and hyaluronuclease at 37°C for 30 min. The digested tissues were washed and filtered through a 30-µm cell straine, and the cell suspensions were stained with the antibody cocktails and measured in the flow cytometers.
Instrument	For flow cytometry:BD FACSCanto II flow cytometer (BD Biosciences) or Fortessa LSR (BD Biosciences).
Software	FlowJo v10.2 software (Tree Star, Inc).
Cell population abundance	Sorted fractions that were send for sequencing were 100% positive for the markers that we stated in the methodology section.
Gating strategy	Aortic endothelial cells were sorted as: gated as live and CD45lowCD31highCD107ahigh.<br>Brown adipose endothelial cells were gated as live and CD45negativeCD31positive.<br>In blood Cells were gated as singlets, live and CD45+CD11b+ myeloid subsets and further gated as CD115+Ly6G- (monocytes) and CD115-Ly6G+ (neutrophils).

☐ Tick this box to confirm that a figure exemplifying the gating strategy is provided in the Supplementary Information.
