# Supplementary material for: Endothelial cannabinoid CB1 receptor deficiency reduces shear stress-induced arterial inflammation and lipid uptake
Source: Nat Commun. 2026 Jul 7;17:5939. doi: 10.1038/s41467-026-75214-2 (PMC13342577; doi:10.1038/s41467-026-75214-2)
Supplement: Supplementary file 3 — Transparent Peer Review file [file 41467_2026_75214_MOESM3_ESM.pdf]

nature portfolio

Peer Review File

Endothelial cannabinoid CB1 receptor deficiency reduces shear stress-induced arterial inflammation and lipid uptake

Corresponding Author: Professor Sabine Steffens

This file contains all reviewer reports in order by version, followed by all author rebuttals in order by version.

Version 0:

Reviewer comments:

Reviewer #3

(Remarks to the Author)
Major concerns:
1. Figure 6: The authors show that CAV1 and ALK1, both implicated in caveolae-mediated LDL transport, are downregulated in Cnr1EC-KO mice. Is CB1's effect on endothelial LDL uptake ALK1-dependent?

2. Evidence for LDL transcytosis remains indirect. The current support relies on GO enrichment for caveolae/membrane rafts, reduced CAV1, and decreased ex vivo DiI-LDL entry, without direct quantification of transcytotic events. Nevertheless, the mechanistic schematic (Figure S13) uses the term "LDL transcytosis." If this term is to be retained, please provide direct evidence of transcytosis (total internal fluorescence microscopy (TIRF) or transendothelial LDL flux assays) to substantiate the claim.

3. Quantitative TEM analysis of endothelial caveolae should be provided to directly visualize their involvement.

4. Increased plasma cholesterol is observed only in Apoe-/- EC-Cnr1 deficiency, not in Ldlr-/- under peripheral CB1 blockade. Please be cautious in concluding that there are "without undesired side effects on plasma cholesterol levels", the outcome appears model and intervention dependent and should be discussed.

5. The Discussion briefly links stronger female effects to estrogen-driven upregulation of endothelial CNR1/CAV1. Given that estrogen is broadly atheroprotective (lipids and anti-inflammation), please explicitly relate this local CB1–CAV1 pathway to systemic benefits, delineating levels of action and context-dependence to reconcile the two.

6. Figure S6C: the Cnr1EC-WT and Cnr1EC-KO panels appear to overlap, please correct the layout. Also, ensure that font sizes are consistent.

Reviewer #4

(Remarks to the Author)
The Authors clearly proved the involvement of endothelial cannabinoid CB1 receptor in atherogenesis by providing multiple lines of evidence obtained both from human aortic endothelial cells (HAoECs) and from specially generated mice with endothelial Cnr1 deficiency on an atherogenic background (referred to as Cnr1EC-KO), as well as in Apoe–/– mice. Moreover, they demonstrated the expression of the CB1-encoding gene CNR1 in arterial endothelial cells of human plaques.
The work is very interesting, well-organized, and the manuscript is overall well-written and easy to read. The noteworthy results are properly summarized in the Abstract. However, the following comments should be addressed to further enhance the quality of the article:
1. In the Introduction, two important publications should be mentioned (by Sugamura et al., 2009; doi: 10.1161/CIRCULATIONAHA.108.811992 and the Authors` own study by Wang et al., 2024; doi: 10.1093/cvr/cvae125), in which the presence of CNR1 in human coronary atherectomy samples was demonstrated. In the current manuscript, the Authors expanded on their previous observations by showing that CNR1 was detectable mainly in an endothelial cluster.

2. Importantly, the Authors followed the SAGER guidelines. However, in the current manuscript they obtained results opposite to those previously published (Wang et al., 2024) regarding the effects of the peripheral CB1 receptor antagonists JD5037 given at a dose of 3 mg/kg i.p. to 8-week-old Ldlr–/– mice. The treatment protected against atherosclerosis either only in male or only in female mice (previous vs. current study). The only difference in the protocol was the moment (4 and 8 weeks after beginning of western diet [WD], respectively) and the time (4 and 8 weeks, respectively) of JD5037 administration. Could the Authors better justify their explanation than simply stating that "it is disease stage-specific", as written in the Discussion? Moreover, some sex-dependent differences could result from the small sample size in male mice [i.e., n=3 in males (Fig. S10) and n=6 in females (Fig. 7)]. Significant difference was obtained in female mice only (Fig. 7), but a strong tendency in the same direction in male mice was noticed (Fig. S10). Why did the Authors include in the manuscript such a small group (n=3)?
3. The Authors clearly explained, through numerous experiments, the interesting mechanism responsible for the enhancement of cholesterol/LDL levels in Cnr1EC-KO (a decrease in caveolae-mediated LDL uptake). However, as appropriately mentioned in the Discussion, "the increase in plasma cholesterol is indeed an endothelial-specific effect of Cnr1 deficiency"; e.g. it was not observed chronic administration of JD5037. Please justify the significance of these observations.
4. The vasorelaxant effect of the CB1 receptor should be discussed, especially in light of the most recent publications from the Hungarian group:
Vass Z, Shenker-Horváth K, Bányai B, Vető KN, Török V, Gém JB, Nádasy GL, Kovács KB, Horváth EM, Jakus Z, Hunyady L, Szekeres M, Dörnyei G. Investigating the Role of Cannabinoid Type 1 Receptors in Vascular Function and Remodeling in a Hypercholesterolemic Mouse Model with Low-Density Lipoprotein-Cannabinoid Type 1 Receptor Double Knockout Animals. Int J Mol Sci. 2024 Sep 2;25(17):9537. doi: 10.3390/ijms25179537.
Bányai B, Vass Z, Kiss S, Balogh A, Brandhuber D, Karvaly G, Kovács K, Nádasy GL, Hunyady L, Dörnyei G, Horváth EM, Szekeres M. Role of CB1 Cannabinoid Receptors in Vascular Responses and Vascular Remodeling of the Aorta in Female Mice. Int J Mol Sci. 2023 Nov 17;24(22):16429. doi: 10.3390/ijms242216429.

5. The Authors stated in the Discussion "we found that the absence of endothelial CB1 increases flow velocity in atheroprone sites of the aorta, ….This resulted in a preserved ejection fraction and fractional shortening after atherogenic diet feeding." However, increased flow velocity was measured at baseline, while cardiac parameters were assessed both at baseline and after 4 weeks of WD. Thus, one cannot compare baseline conditions with those induced by WD.
6. The figures should be described in more detail. I suggest clearly indicating in each panel (e.g., A–G) whether it refers to HaoECs, female mice, or male mice, treated or not treated with WD. Moreover, all abbreviations in figure legends should be explain (e.g., OSS in Fig. 2). In addition, please clarify what is meant by "endothelial 1" and "endothelial 2". Finally, carefully check and ensure scale bars uniformity between comparable results in female and male mice (both in the main text and in the Supplementary Materials).
7. The Authors' final conclusion is: "peripheral CB1 antagonists may hold promise as an effective therapeutic strategy for treating atherosclerosis and related metabolic disorders". In this context, all potential targets of peripheral CB1 antagonists should be briefly listed in the Discussion.
8. Page 6, description of Fig. 2. The Authors state: "Significantly less vascular leakage was observed in the aortas of Cnr1EC-KO mice, particularly at atheroprone sites such as the inner curvature of the aortic arch, indicating preserved endothelial integrity in Cnr1EC-KO mice (Figure 2E-F)." This statement overstates the data. The statistical analysis does not show a significant difference between the aortic arch and the descending aorta. Moreover, values in the aortic arch of Cnr1EC-WT mice were approximately 4 times higher than in the descending aorta. Please revise the text so that only statistically significant changes are described.
9. Legend for supplemental Figure S8. Impact of endothelial Cnr1 deficiency on metabolic parameters and plaque in males. This is incorrect, as Figure S8 actually includes results obtained in female mice.
10. Page 5. The Authors state: "HAoECs had a more elongated …… (Figure 1E)." However, Fig.1 presents results obtained from mice, not from human cells.
11. Why did the Authors cite their own publication by Wang et al. (2024) from bioRxiv rather than the peer-reviewed version in Cardiovasc Res (doi: 10.1093/cvr/cvae125)?

Version 1:

Reviewer comments:

Reviewer #3

(Remarks to the Author)
1. The mechanism between systemic estrogen and endothelial CB1 is still primarily based on literature-derived inferences rather than direct evidence. It is suggested that the authors include an additional discussion by integrating the results of their bioinformatics predictions.
2. Lipid uptake is only the initial stage of subendothelial lipid deposition and plaque formation. It is recommended that the authors add a section in the discussion to explore the potential correlation between 'uptake' and 'transcytosis,' and acknowledge that transcytosis kinetics were not directly measured in this study. This would help readers achieve a more comprehensive understanding of the role of CB1 in lipid deposition during atherosclerosis.

Reviewer #4

(Remarks to the Author)
The authors have significantly modified their manuscript from the original version previously submitted and have effectively responded to this reviewer's criticisms and suggestions.

Version 2:

Reviewer comments:

Reviewer #3

(Remarks to the Author)
None

Open Access This Peer Review File is licensed under a Creative Commons Attribution 4.0 International License, which permits use, sharing, adaptation, distribution and reproduction in any medium or format, as long as you give appropriate credit to the original author(s) and the source, provide a link to the Creative Commons license, and indicate if changes were made.
In cases where reviewers are anonymous, credit should be given to 'Anonymous Referee' and the source.
The images or other third party material in this Peer Review File are included in the article's Creative Commons license, unless indicated otherwise in a credit line to the material. If material is not included in the article's Creative Commons license and your intended use is not permitted by statutory regulation or exceeds the permitted use, you will need to obtain permission directly from the copyright holder.
To view a copy of this license, visit https://creativecommons.org/licenses/by/4.0/

Point-by-point response to referees' comments

We would like to thank the reviewers for the thoughtful assessment of our manuscript as well as for the constructive and helpful comments; all of which were carefully considered. Here is a detailed point-by-point reply to all the comments raised by the referees. All changes in the manuscript are highlighted in red.

Reviewer #3:

Major concerns:

1. Figure 6: The authors show that CAV1 and ALK1, both implicated in caveolae-mediated LDL transport, are downregulated in Cnr1EC-KO mice. Is CB1's effect on endothelial LDL uptake ALK1-dependent?

Thank you for raising this question, which prompted us to carry out an additional mechanistic in vitro experiment. We found that siRNA-mediated ALK1 silencing in primary human aortic endothelial cells (HAoECs) blunted the effect of the CB1 agonist ACEA in inducing Dil-LDL uptake. These new data have been added to the revised manuscript (**Supplementary Fig. 9h,** Results p. 12, Discussion p. 16).

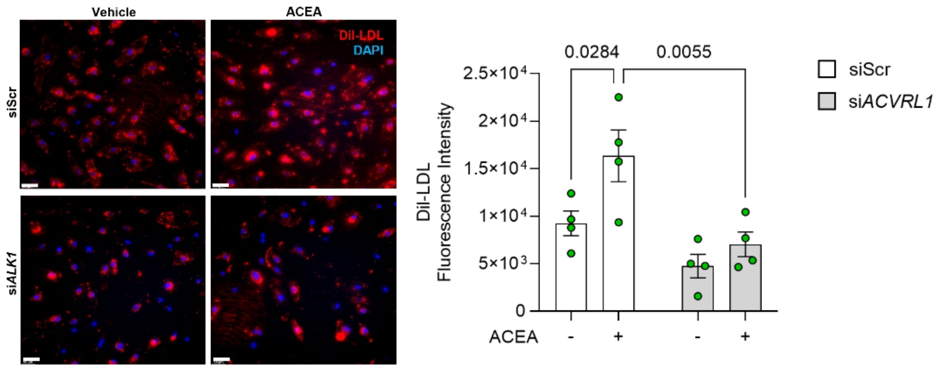

Figure, left: Representative images of female HoAECs pre-treated with siScr (Scramble) or siACVRL1 exposed to OSS with Vehicle or ACEA stained with Dil-LDL (red) and nuclei (blue) (scale bar, 20 µm). **Right**: Quantification of Dil-LDL from g. Data are shown as mean ± s.e.m. and two-way ANOVA with Sidak post hoc was applied. Each data point represents one individual human sample, collected in at least 2 independent experiments.

2. Evidence for LDL transcytosis remains indirect. The current support relies on GO enrichment for caveolae/membrane rafts, reduced CAV1, and decreased ex vivo DiI-LDL entry, without direct quantification of transcytotic events. Nevertheless, the mechanistic schematic (Figure S13) uses the term "LDL transcytosis." If this term is to be retained, please provide direct evidence of transcytosis (total internal fluorescence microscopy (TIRF) or transendothelial LDL flux assays) to substantiate the claim.

We apologize for the inaccurate use of the term LDL transcytosis in the final mechanistic summary figure, which is not supported by experimental evidence. In our study, we focused on LDL uptake, which we found to be regulated by CB1. Hence, we removed the term LDL transcytosis from the Figure and in the manuscript text.

3. Quantitative TEM analysis of endothelial caveolae should be provided to directly visualize their involvement.

Thank you for this valuable suggestion. We performed TEM to visualize endothelial caveolae. The quantification of apical caveolae in aortic arches supports our hypothesis that deficiency of *Cnr1* reduces their number on the endothelial luminal site. These important findings have been added to the revised **Fig. 6c-d**.

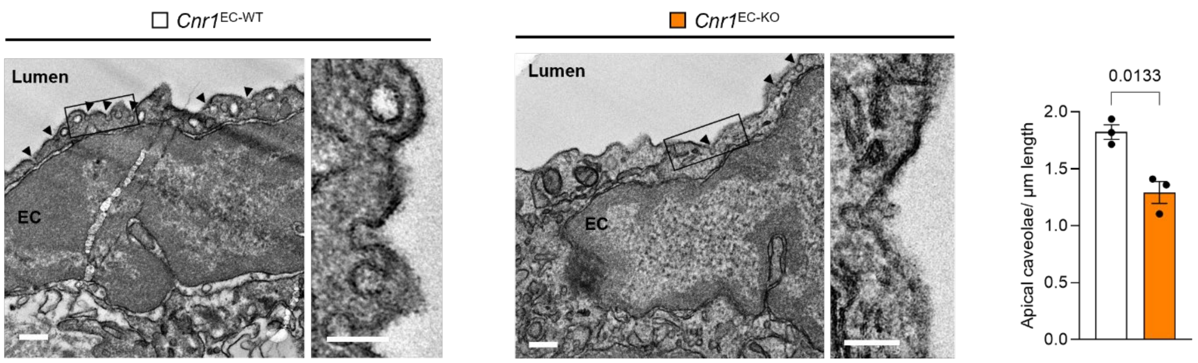

Figure, left: TEM images visualizing endothelial caveolae from aortic arches of *Cnr1*EC-WT and *Cnr1*EC-KO mice after 4 weeks WD (scale bar: overview- 200 nm, zoom in- 100nm) EC- endothelial cell. **Right:** Quantification of apical caveolae (n= 3 mice per group, each dot represents an average of 20 analysed images).

4. Increased plasma cholesterol is observed only in Apoe-/- EC-Cnr1 deficiency, not in Ldlr-/- under peripheral CB1 blockade. Please be cautious in concluding that there are "without undesired side effects on plasma cholesterol levels", the outcome appears model and intervention dependent and should be discussed.

We thank the reviewer for raising this critical point and added some discussion on p. 17 of the revised manuscript:

*"The increased plasma cholesterol levels were only observed in Apoe$^{-/-}$ mice with endothelial Cnr1 deficiency, but not in Ldlr$^{-/-}$ mice chronically treated with the peripheral CB1 antagonist, indicating a potential clinical benefit without undesired side effects on plasma cholesterol levels. The differential effects on cholesterol levels in the two atherosclerosis knockout strains hint to a model and intervention dependent effect. A previous study in Apoe$^{-/-}$ mice treated with the CB1 antagonist rimonabant also reported comparable serum cholesterol levels in vehicle and antagonist treated mice,[14] which suggests that the increase in plasma cholesterol might be indeed an endothelial-specific effect of Cnr1 deficiency. Other studies even reported lower plasma cholesterol levels in Ldlr$^{-/-}$ mice or APOE*3-Leiden.CETP mice receiving higher doses of rimonabant.[13, 59] At lower dose, rimonabant reduced atherosclerosis development in Ldlr$^{-/-}$ without affecting cholesterol levels.[13] Nevertheless, systemic cholesterol levels should be carefully monitored in future studies further exploiting the therapeutic benefits of peripheral CB1 antagonism."*

5. The Discussion briefly links stronger female effects to estrogen-driven upregulation of endothelial CNR1/CAV1. Given that estrogen is broadly atheroprotective (lipids and anti-inflammation), please explicitly relate this local CB1–CAV1 pathway to systemic benefits, delineating levels of action and context-dependence to reconcile the two.

We have revised the statement in the discussion on p. 17 accordingly:

"In the present study, the effects of endothelial Cnr1 deficiency on atherosclerotic plaque size and endothelial CAV1 expression were more pronounced in female mice compared to males, which might be at least partially explained by stimulatory effects of estrogens on CNR1 and CAV1 expression. However, this local effect on the endothelium must be viewed in a systemic context, as estrogen has an overall atheroprotective effect.[62] Of note, a recent study reported increased vasorelaxant responses to acetylcholine and estradiol in aortic ring segments of Cnr1$^{-/-}$ mice compared to corresponding WT mice,[51] which supports a vasculoprotective effect in absence of CB1 signaling."

6. Figure S6C: the Cnr1EC-WT and Cnr1EC-KO panels appear to overlap, please correct the layout. Also, ensure that font sizes are consistent.

We have carefully revised the Figure panels and adjusted the font size.

Reviewer #4:

The Authors clearly proved the involvement of endothelial cannabinoid CB1 receptor in atherogenesis by providing multiple lines of evidence obtained both from human aortic endothelial cells (HAoECs) and from specially generated mice with endothelial Cnr1 deficiency on an atherogenic background (referred to as Cnr1EC-KO), as well as in Apoe−/− mice. Moreover, they demonstrated the expression of the CB1-encoding gene CNR1 in arterial endothelial cells of human plaques.
The work is very interesting, well-organized, and the manuscript is overall well-written and easy to read. The noteworthy results are properly summarized in the Abstract. However, the following comments should be addressed to further enhance the quality of the article:

1. In the Introduction, two important publications should be mentioned (by Sugamura et al., 2009; doi: 10.1161/CIRCULATIONAHA.108.811992 and the Authors` own study by Wang et al., 2024; doi: 10.1093/cvr/cvae125), in which the presence of CNR1 in human coronary atherectomy samples was demonstrated. In the current manuscript, the Authors expanded on their previous observations by showing that CNR1 was detectable mainly in an endothelial cluster.

We agree that these 2 studies are relevant, and already cited our own study in the introduction (ref. #18), while the study of Sugamura et al (2009) was only mentioned in the context of the human plaque expression data the results section. As suggested by the reviewer, we now also refer to Sugamura et al (2009) in the introduction (p. 5):

"We have recently shown that myeloid-specific Cnr1 deficiency limits atherosclerosis by inhibiting the recruitment and proliferation of arterial macrophages.[18] Sugamura and colleagues previously reported that the systemically acting CB1 antagonist rimonabant exerted anti-inflammatory effects in macrophages,[19] which was corroborated by another study.[13]"

2. Importantly, the Authors followed the SAGER guidelines. However, in the current manuscript they obtained results opposite to those previously published (Wang et al., 2024) regarding the effects of the peripheral CB1 receptor antagonists JD5037 given at a dose of 3 mg/kg i.p. to 8-week-old Ldlr−/− mice. The treatment protected against atherosclerosis either only in male or only in female mice (previous vs. current study). The only difference in the protocol was the moment (4 and 8 weeks after beginning of western diet [WD], respectively) and the time (4 and 8 weeks, respectively) of JD5037 administration. Could the Authors better justify their explanation than simply stating that "it is disease stage-specific", as written in the Discussion? Moreover, some sex-dependent differences could result from the small sample size in male mice [i.e., n=3 in males (Fig. S10) and n=6 in females (Fig. 7)]. Significant difference was obtained in female mice only (Fig. 7), but a strong tendency in the same direction in male mice was noticed (Fig. S10). Why did the Authors include in the manuscript such a small group (n=3)?

Thank you for highlighting this limitation. We have performed additional experiments to increase the sample size of the male data in **Supplemental Fig. 10** to n=6).

To better explain the sex- and time point dependent differences are puzzling, we have extended the discussion accordingly (p. 17-18):

“Our previous study focused on the effects of myeloid Cnr1 deficiency, which were already evident at earlier time points in male mice. For this reason, we tested the effect of 4 weeks treatment with the peripheral antagonist JD5037 in the previous study. Effects of myeloid Cnr1 deficiency were also noted in female mice, but only at the late time point after 16 weeks diet (i.e. smaller necrotic core size in aortic root plaques and smaller aortic arch plaque size). Thus, our previous study using 8 weeks WD and treatment during the last 4 weeks of diet was too short to observe effects on atherosclerotic plaque development in female mice. As to the underlying mechanisms that contribute to the differential stage-dependent effects in male versus female mice, we showed an involvement of estrogen signaling in the regulation of chemokine receptor surface expression on monocytes, which is relevant for arterial monocyte recruitment, a key mechanism of early atherogenesis. This may explain why Cnr1 deficiency or CB1 antagonism during atherogenesis has more pronounced effects in males. Even though no difference on plaque size was observed in male mice any more after 16 weeks of atherogenic diet and receiving JD5037 during the last 8 weeks of diet, there was a significant decrease in adhesion molecule expression notable at this advanced stage of plaque development in both male and female mice. Furthermore, we also observed a tendency for lower CAV1 expression in aortic roots of male Cnr1EC-KO mice ($p = 0.0649$) and significantly reduced LDL uptake by AM281 in male HAoECs. However, CAV1 was not significantly regulated by AM281 in male hAoECs. Therefore, we argue that our previous and the present study clearly highlight that the effect of genetic Cnr1 deletion or pharmacological antagonism of the receptor shows distinct strength of effects in macrophages versus endothelial cells, which is dependent of the biological sex and may be partially explained by sex hormonal effects.”

3. The Authors clearly explained, through numerous experiments, the interesting mechanism responsible for the enhancement of cholesterol/LDL levels in Cnr1EC-KO (a decrease in caveolae-mediated LDL uptake). However, as appropriately mentioned in the Discussion, “the increase in plasma cholesterol is indeed an endothelial-specific effect of Cnr1 deficiency”; e.g. it was not observed chronic administration of JD5037. Please justify the significance of these observations.

We have discussed the potential significance of these observations in the revised manuscript (p. 17):

*“The increased plasma cholesterol levels were only observed in Apoe$^{-/-}$ mice with endothelial Cnr1 deficiency, but not in Ldlr$^{-/-}$ mice chronically treated with the peripheral CB1 antagonist, indicating a potential clinical benefit without undesired side effects on plasma cholesterol levels. The differential effects on cholesterol levels in the two atherosclerosis knockout strains hint to a model-dependent effect. However, a previous study in Apoe$^{-/-}$ mice treated with the CB1 antagonist rimonabant also reported comparable serum cholesterol levels in vehicle and antagonist treated mice,[14] which suggests that the increase in plasma cholesterol might be indeed an endothelial-specific effect of Cnr1 deficiency. Other studies even reported lower plasma cholesterol levels in Ldlr$^{-/-}$ mice or APOE*3-Leiden.CETP mice receiving higher doses of rimonabant. [13,61] At lower dose, rimonabant reduced atherosclerosis development in Ldlr$^{-/-}$ without affecting cholesterol levels. [13] Nevertheless, systemic cholesterol levels should be carefully monitored in future studies further exploiting the therapeutic benefits of peripheral CB1 antagonism.”*

4. The vasorelaxant effect of the CB1 receptor should be discussed, especially in light of the most recent publications from the Hungarian group:
Vass Z, Shenker-Horváth K, Bányai B, Vető KN, Török V, Gém JB, Nádasy GL, Kovács KB, Horváth EM, Jakus Z, Hunyady L, Szekeres M, Dörnyei G. Investigating the Role of Cannabinoid Type 1 Receptors in Vascular Function and Remodeling in a Hypercholesterolemic Mouse Model with Low-Density Lipoprotein-Cannabinoid Type 1 Receptor Double Knockout Animals. Int J Mol Sci. 2024 Sep 2;25(17):9537. doi: 10.3390/ijms25179537.

Bányai B, Vass Z, Kiss S, Balogh A, Brandhuber D, Karvaly G, Kovács K, Nádasy GL, Hunyady L, Dörnyei G, Horváth EM, Szekeres M. Role of CB1 Cannabinoid Receptors in Vascular Responses and Vascular Remodeling of the Aorta in Female Mice. Int J Mol Sci. 2023 Nov 17;24(22):16429. doi: 10.3390/ijms242216429.

Thank you for highlighting these important recent studies. We have discussed the vasorelaxant effect of the CB1 receptor in the revised manuscript, referring to the two publications mentioned by the reviewer (p. 15):

More recently, increased vasorelaxant responses were shown in Cnr1$^{-/-}$ mice compared to corresponding WT controls,[51] and In hypercholesterolemic mice with Ldlr$^{-/-}$ background and subjected to WD, vascular dysfunction was attenuated in Ldlr$^{-/-}$Cnr1$^{-/-}$ double deficient mice.[52] In particular, Ldlr$^{-/-}$Cnr1$^{-/-}$ mice had a reduced systolic and diastolic blood pressure, and were protected against WD-induced blood pressure increases and reductions in endothelium-dependent vasorelaxation.

5. The Authors stated in the Discussion "we found that the absence of endothelial CB1 increases flow velocity in atheroprone sites of the aorta, ….This resulted in a preserved ejection fraction and fractional shortening after atherogenic diet feeding." However, increased flow velocity was measured at baseline, while cardiac parameters were assessed both at baseline and after 4 weeks of WD. Thus, one cannot compare baseline conditions with those induced by WD.

The reviewer raises an important point. We have also measured the arterial flow velocity in mice after 4 weeks WD, and added and discussed these data in the revised manuscript (**Supplementary Fig. 3e-g**; Results, p. 7, Discussion p. 15). In baseline conditions, we observed a higher peak flow velocity in atheroprone regions of the aorta in *Cnr1$^{EC\text{-}KO}$* mice compared to *Cnr1$^{EC\text{-}WT}$* mice (Supplementary Fig. 3e-g), which is in agreement with previous reports assessing flow dynamics in 2-months old *Ldlr$^{-/-}$* and *Cav1$^{-/-}$Ldlr$^{-/-}$* mice. Under WD, the peak flow velocity dropped in atheroprone regions of the aortic arch and aortic roots of *Cnr1$^{EC\text{-}KO}$* mice, but remained unchanged in *Cnr1$^{EC\text{-}WT}$* mice. Together, these findings suggest that endothelial *Cnr1* deficiency affects the regulation of blood flow dynamics, which is likely related to the downregulation of caveolae, known to play an important role in sensing and transducing hemodynamic changes into biochemical signals to regulate vascular function (Yu et al JCI 2006; PMID: 16670769).

6. The figures should be described in more detail. I suggest clearly indicating in each panel (e.g., A–G) whether it refers to HaoECs, female mice, or male mice, treated or not treated with WD. Moreover, all abbreviations in figure legends should be explain (e.g., OSS in Fig. 2). In addition, please clarify what is meant by "endothelial 1" and "endothelial 2". Finally, carefully check and ensure scale bars uniformity between comparable results in female and male mice (both in the main text and in the Supplementary Materials).

Thanks for highlighting these issues. We have carefully revised the Figures and legends for consistence and clarity.

To answer the question regarding endothelial 1 and endothelial 2: these are well defined endothelial cell clusters identified in single cell RNA sequencing datasets based on a set of distinct markers. endothelial cluster 1 (expressing *ACKR1*, *RGCC*, *NR2F2*) is of venular origin and considered as pro-angiogenic subset, thought to contribute to leaky neovessels and immune cell trafficking. Endothelial cluster 2 is characterized by the expression of arterial endothelial cell markers (expressing *BMX*, *MECOM*, *GJA5*); PMID: 40931012). To clarify this, we have rephrased the data description accordingly (p. 6).

7. The Authors' final conclusion is: "peripheral CB1 antagonists may hold promise as an effective therapeutic strategy for treating atherosclerosis and related metabolic disorders". In this context, all potential targets of peripheral CB1 antagonists should be briefly listed in the Discussion.

We have extended the discussion by adding potential peripheral CB1 antagonist targets (p. 14):

"Thus, antagonizing peripheral CB1 signaling may reduce cholesterol uptake in the arterial wall, thereby contributing to pleiotropic beneficial effects of CB1 blocking in atherosclerosis and related metabolic alterations. In particular, previous experimental studies using peripheral CB1 antagonists have shown reductions in body weight, improved glucose tolerance, decreased hepatic steatosis, and enhanced thermogenesis.[15, 16, 17, 48, 49] In this context, a recent study has highlighted the relevance of CB1 signaling in gut sensory neurons in regulating energy homeostasis.[50]"

8. Page 6, description of Fig. 2. The Authors state: "Significantly less vascular leakage was observed in the aortas of Cnr1EC-KO mice, particularly at atheroprone sites such as the inner curvature of the aortic arch, indicating preserved endothelial integrity in Cnr1EC-KO mice (Figure 2E-F)." This statement overstates the data. The statistical analysis does not show a significant difference between the aortic arch and the descending aorta. Moreover, values in the aortic arch of Cnr1EC-WT mice were approximately 4 times higher than in the descending aorta. Please revise the text so that only statistically significant changes are described.

We have rephrased the statement in the revised manuscript (p. 7) accordingly:

"Significantly less vascular leakage was observed in the aortic arch of $Cnr1^{EC}$-KO compared to $Cnr1^{EC\text{-}WT}$ mice, indicating preserved endothelial integrity in absence of Cnr1 (Fig. 2e-f)."

9. Legend for supplemental Figure S8. Impact of endothelial Cnr1 deficiency on metabolic parameters and plaque in males. This is incorrect, as Figure S8 actually includes results obtained in female mice.

Thanks for reading through this carefully. We have corrected the **Supplementary Fig. 8** legend title as following: *"Impact of endothelial Cnr1 deficiency on metabolic parameters."*

10. Page 5. The Authors state: "HAoECs had a more elongated …… (Figure 1E)." However, Fig.1 presents results obtained from mice, not from human cells.

We thank the reviewer for spotting this mistake. This sentence refers to panel **Fig. 1f**, which we have corrected accordingly (see p. 6).

11. Why did the Authors cite their own publication by Wang et al. (2024) from bioRxiv rather than the peer-reviewed version in Cardiovasc Res (doi: 10.1093/cvr/cvae125)?

Thank you for carefully reading through our work. At the time of writing the first draft of the current manuscript, our other study in CVR was still in revision process. When updating this manuscript for submission, we overlooked updating the reference.

Point-by-point response to referees' comments

We would like to thank the reviewers for the thoughtful assessment of our manuscript as well as for the constructive and helpful comments; all of which were carefully considered. Here is a detailed point-by-point reply to the remaining comments raised by Reviewer 3. All changes in the manuscript are highlighted in red.

Reviewer #3:

1. The mechanism between systemic estrogen and endothelial CB1 is still primarily based on literature-derived inferences rather than direct evidence. It is suggested that the authors include an additional discussion by integrating the results of their bioinformatics predictions.

We extended the discussion of the link between estrogen and endothelial CB1 receptors and integrated additional bioinformatic predictions. Given that the literature supports an interaction between estrogen receptors ERα and ERβ and caveolin in various cell types,[1] we performed a more in-depth bioinformatic analysis of our murine aortic endothelial RNA-seq dataset to assess a possible regulation of estrogen signaling by CB1. However, the expression levels of estrogen receptor ERα and Erβ-encoding genes (Esr1, Esr2) were comparable between WT and *Cnr1*-deficient endothelial cells. Using GSEA, we identified the gene set "Reactome Estrogen Biosynthesis" with a normalized enrichment score (NES) of -1.41, a false discovery rate (FDR)-adjusted q-value of 0.23, and a nominal p-value of 0.084, which is above our defined cut-off for significance (P<0.05). When comparing the expression levels of the genes linked to this gene set, we found a significantly lower expression of *Hsd17b14* (p=0.0359; unpaired T-Test), which encodes an enzyme involved in steroid metabolism. Thus, CB1 might exhibit a moderate effect on estrogen signaling.

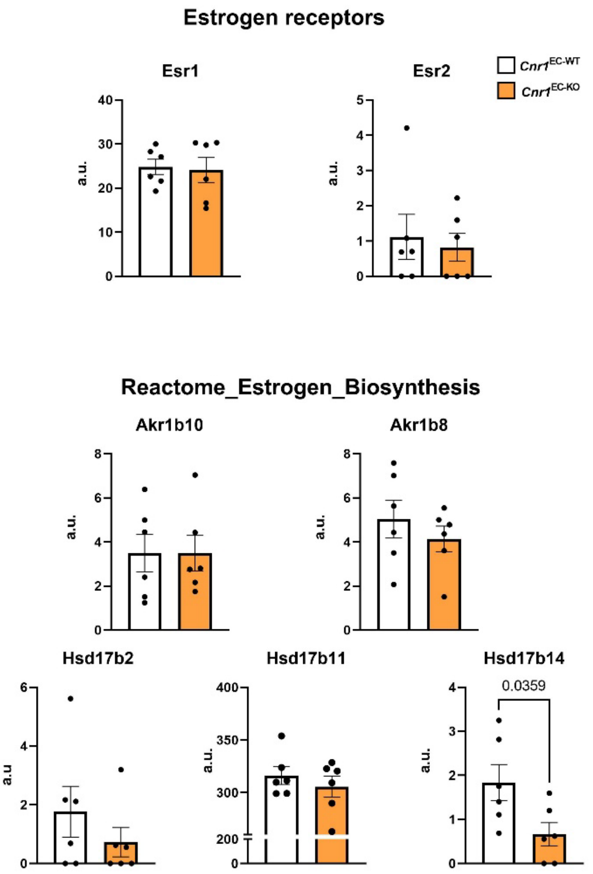

To better understand the interaction between CB1 and oestrogen signalling, we conducted an additional in vitro experiment. We assessed the impact of E2 on CAV1 protein expression and LDL uptake in human female aortic endothelial cells transfected with scrambled (siScr) or *CNR1* silencing RNA (*siCNR1*), respectively. In *siScr*-treated HAECs with preserved CB1 expression, E2 significantly reduced CAV1 and LDL uptake, which was also observed when silencing *CNR1*, compared to the siScr-vehicle group. However, no further decrease in CAV1 expression and LDL uptake was observed in response to E2 after silencing *CNR1* (see below, and added as new **Supplementary Fig. 13d-f**). Together, this suggests that estrogen and endothelial CB1 signaling interfere with the same cellular pathway in endothelial cells, with estrogen playing an atheroprotective role, and CB1 a proatherogenic one.

We have added these novel data to the revised manuscript and also revised and extended the discussion on the complex regulatory interplay between CB1-dependent regulation of LDL uptake and estrogen signaling (p.16):

"In the present study, the effects of endothelial Cnr1 deficiency on atherosclerotic plaque size and endothelial CAV1 expression were more pronounced in female mice than in males, which might be at least partially explained by the stimulatory effects of estrogens on CNR1 ~~and CAV1~~ expression. However, this local effect on the endothelium must be viewed in a systemic context, as estrogen has an overall atheroprotective effect.66 In support of this, a previous in vitro study reported higher LDL transcytosis in human coronary artery ECs from males and postmenopausal females compared to ECs from younger females.67 Adding estrogen reduced transcytosis, which was due to G-protein-coupled estrogen receptor-dependent downregulation of SR-BI. In agreement with this, our in vitro experiments showed reduced endothelial LDL uptake upon estrogen treatment, associated with decreased CAV1 protein expression. The finding that no further decrease in CAV1 expression and LDL uptake was observed in response to E2 after silencing CNR1 suggests that estrogen and endothelial CB1 signaling interfere with the same cellular pathway in endothelial cells, but with opposing effects. Literature data further support an interaction between estrogen receptors ERα and ERβ and caveolin in various cell types,68 suggesting a complex regulatory interplay between CB1-dependent regulation of caveolae and estrogen signaling."

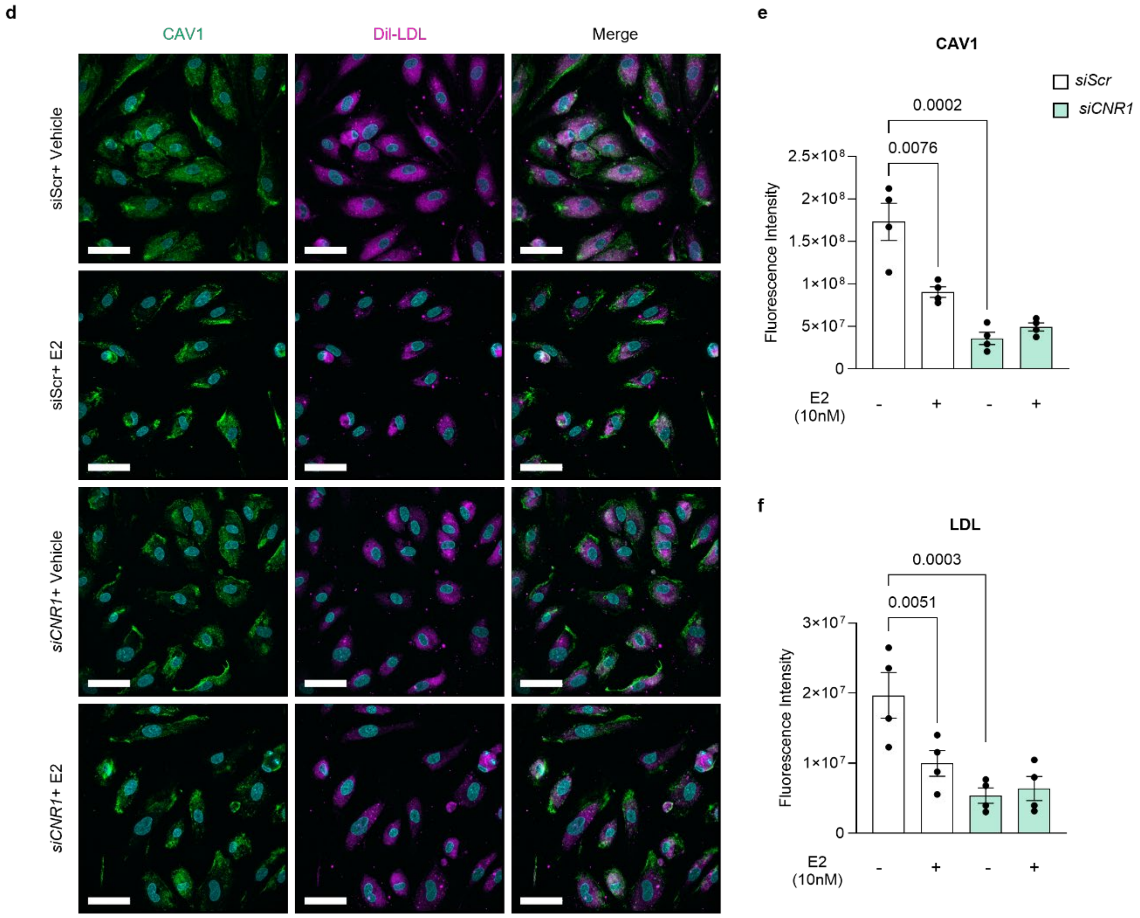

2. Lipid uptake is only the initial stage of subendothelial lipid deposition and plaque formation. It is recommended that the authors add a section in the discussion to explore the potential correlation between 'uptake' and 'transcytosis,' and acknowledge that transcytosis kinetics were not directly measured in this study. This would help readers achieve a more comprehensive understanding of the role of CB1 in lipid deposition during atherosclerosis.

Following the reviewer´s suggestion, we have extended the discussion on lipid uptake and transcytosis (p. 15):

“It should be acknowledged that only the initial step of subendothelial lipid deposition was assessed in our study, while we did not directly measure lipid transcytosis. Thus, the observed changes in caveolae-dependent LDL uptake and plaque lipid content are merely correlational, even though LDL transcytosis is thought to be rate-limiting in atherosclerosis development.[2] Multiple steps are involved in transcellular transport, and cumulative evidence suggests that caveolar transcytosis across endothelial cells is the dominant pathway, which includes caveolae formation, undocking, trafficking, and docking.”

References

1. Luoma JI, Boulware MI, Mermelstein PG. Caveolin proteins and estrogen signaling in the brain. *Molecular and Cellular Endocrinology* **290**, 8-13 (2008).

2. Bolanle IO, de Liedekerke Beaufort GC, Weinberg PD. Transcytosis of LDL Across Arterial Endothelium: Mechanisms and Therapeutic Targets. *Arterioscler Thromb Vasc Biol* **45**, 468-480 (2025).

Point-by-point response to referees' comments

We would like to thank the reviewers for the thoughtful assessment of our manuscript. No further comments were raised by the reviewers.

We have addressed all remaining editorial revisions in accordance with the author's instructions.
